# Rational construction of genome-reduced Burkholderiales chassis facilitates efficient heterologous production of natural products from proteobacteria

**DOI:** 10.1038/s41467-021-24645-0

**Published:** 2021-07-23

**Authors:** Jiaqi Liu, Haibo Zhou, Zhiyu Yang, Xue Wang, Hanna Chen, Lin Zhong, Wentao Zheng, Weijing Niu, Sen Wang, Xiangmei Ren, Guannan Zhong, Yan Wang, Xiaoming Ding, Rolf Müller, Youming Zhang, Xiaoying Bian

**Affiliations:** 1 Helmholtz International Lab for Anti-Infectives, Shandong University-Helmholtz Institute of Biotechnology, State Key Laboratory of Microbial Technology, Shandong University, Qingdao, Shandong China; 2 Core Facilities for Life and Environmental Sciences, Shandong University, Qingdao, Shandong China; 3 College of Marine Life Sciences, and Institute of Evolution & Marine Biodiversity, Ocean University of China, Qingdao, China; 4 Collaborative Innovation Center for Genetics and Development, State Key Laboratory of Genetic Engineering, Department of Microbiology, School of Life Sciences, Fudan University, Shanghai, China; 5 Helmholtz International Lab for Anti-Infectives, Department of Microbial Natural Products, Helmholtz Institute for Pharmaceutical Research, Helmholtz Centre for Infection Research and Department of Pharmacy, Saarland University, Saarbrücken, Germany

**Keywords:** Metabolic engineering, Natural products, Bacterial synthetic biology, Genomic engineering

## Abstract

Heterologous expression of biosynthetic gene clusters (BGCs) avails yield improvements and mining of natural products, but it is limited by lacking of more efficient Gram-negative chassis. The proteobacterium *Schlegelella brevitalea* DSM 7029 exhibits potential for heterologous BGC expression, but its cells undergo early autolysis, hindering further applications. Herein, we rationally construct DC and DT series genome-reduced *S. brevitalea* mutants by sequential deletions of endogenous BGCs and the nonessential genomic regions, respectively. The DC5 to DC7 mutants affect growth, while the DT series mutants show improved growth characteristics with alleviated cell autolysis. The yield improvements of six proteobacterial natural products and successful identification of chitinimides from *Chitinimonas koreensis* via heterologous expression in DT mutants demonstrate their superiority to wild-type DSM 7029 and two commonly used Gram-negative chassis *Escherichia coli* and *Pseudomonas putida*. Our study expands the panel of Gram-negative chassis and facilitates the discovery of natural products by heterologous expression.

## Abstract (web summary)

An efficient chassis for heterologous expression of biosynthetic gene clusters (BGCs) from Gram-negative bacteria is still unavailable. Here, the authors report rational construction of genome-reduced Burkholderials chassis to facilitate production of a class of new compounds by expressing BGC from *Chitinimonas koreensis*.

## Introduction

Bacteria are important sources of therapeutic agents and eco-friendly biopesticides as well as bioactive compounds with biotechnological applications^1,2^. Bacterial genomes still contain a large number of cryptic or “silent” natural product biosynthetic gene clusters (BGCs), and the encoded products are an underexplored source of bioactive metabolites^3–6^. The heterologous expression of BGCs is not only an effective approach to optimize the production yields of targeted compounds but also a prevalent strategy to mine cryptic secondary metabolites from genome sequences^7–9^, which benefits from crucial advances in cloning and assembly techniques of large BGCs, such as Linear plus Linear Homologous Recombination (LLHR), Transformation-associated Recombination (TAR) cloning, Cas9-Assisted Targeting of CHromosome segments (CATCH)^10–13^. However, the lack of more efficient hosts is still one of the limits for robust heterologous expression, especially for BGCs from bacterial species that have received relatively little attention.

The biosynthesis and heterologous expression of natural products from Gram-positive actinobacteria have been extensively investigated for many decades, and a handful of robust chassis, especially genome-reduced derivatives of *Streptomyces* spp.^14–19^, have been established and widely used for efficient heterologous expression of actinobacterial BGCs. Thus, rational genome reduction is expected to serve as a feasible strategy to construct ideal chassis hosts. In the last two decades, natural products from Gram-negative proteobacteria, such as pseudomonads, myxobacteria, and Burkholderiales, have become increasingly accepted as an emerging, prolific and underexplored source of compounds with bioactive potential^20–23^. To access the untapped biosynthetic potential of previously overlooked proteobacterial species, an ideal chassis strain with robust growth characteristics, fluent genetic manipulation, a low secondary metabolite background, and high production yields is required. Recognizing wild-type (WT) bacterial species as ideal hosts is still far from reality, so rational genome streamlining is important for the realization of this goal.

Proteobacteria undergoing genome reduction via elimination of nonessential genes tend to exhibit improved properties, such as the increased electroporation efficiency and genetic stability of recombinant plasmids in genome-reduced *Escherichia coli* with 14.3% of the entire genome deleted^24^. The endogenous BGCs and genes encoding enzymes that mediate transposition and horizontal gene transfer, e.g., transposases, insertion sequence (IS) elements, and phage-related proteins, are considered dispensable for cellular processes, as the former compete for precursor supply and the latter may result in instability^25–27^. Rational reduction of most prophages, flagellar-related genes and some mobile elements in a *Pseudomonas putida* chromosome was found to lead to a very significant improvement in heterologous gene expression and synthesis of bioplastics while physiological robustness was maintained^25,28–31^. Genome-reduced *Pseudomonas chlororaphis* with deletion of five secondary metabolic BGCs and 17 strain-specific regions showed improved production of native phenazines^32^. Thus, elimination of nonessential parasitic elements, mobile DNA segments and useless surface structures in a bioreactor to improve biotechnological performance, together with appropriate deletion of native BGCs to decrease metabolite background, could be two reasonable routes of chassis optimization for heterologous production of natural products.

The β-proteobacterial strain *Schlegelella brevitalea* DSM 7029, formerly known as *Polyangium brachysporum* K481-B101 or Burkholderiales strain DSM 7029, was previously utilized as a heterologous host to produce proteobacterial nonribosomal peptide/polyketide (NRP/PK) natural products with relatively high yields, such as epothilone and vioprolide from myxobacteria (δ-proteobacteria) and rhizomide from Burkholderiales (β-proteobacteria)^33–35^. This strain possesses essential biosynthetic elements, e.g., 4′-phosphopantetheinyl transferase, and generates the important PK extender unit methylmalonyl-CoA that is not produced at a detectable level in another commonly used Gram-negative chassis, *Pseudomonas putida*^33,36^, for the efficient biosynthesis of PKs and NRPs^37^. The DSM 7029 strain can form single colonies on solid agar plates, and exhibits a faster growth cycle, with a doubling time of 1 h in normal CYMG medium, than many myxobacteria species (δ-proteobacteria, a prolific source of bioactive natural products), such as the frequently used chassis for myxobacteria *Myxococcus xanthus* (~5 h) or the native producer of anticancer epothilones *Sorangium cellulosum* (~16 h)^33^. Thus, the DSM 7029 strain shows potential as a candidate chassis for production of natural products from Gram-negative proteobacteria. The comparison of other widely used chassis with the DSM 7029 strain is also summarized here (Supplementary Table 1). However, during our deep investigation of this strain to improve the production of epothilones, we found that the early autolysis of DSM 7029 led to most cell death after 48 h of fermentation, which also has been recently reported to occur in many Gram-negative bacteria^38–40^, severely affecting the biomass of DSM 7029 and ultimately restricting the heterologous production yields of desired compounds^41^. Sucrose addition to the medium delayed the cell death and extended the fermentation cycle^41^, circumventing this important negative factor in industrial fermentation production to some degree. We intended to perform rational genome reduction to defer the autolysis and/or to increase biomass for robust biotechnological performance of DSM 7029 because the existence and expression of putative regulatory genes and/or some unknown autolytic genes are capable of causing cell lysis^39,42–45^. We recently established an electroporation procedure and efficient genetic engineering system based on the bacteriophage recombinase pair in this strain, dramatically facilitating precise insertion of potent promoters to drive cryptic BGCs for discovery of natural products produced by strain DSM 7029 and those produced by other Burkholderiales species and providing a fluent technological stage for further optimization^46–48^. We also discovered and characterized several strong constitutive promoters in strain DSM 7029, which are beneficial for the optimization of the yield of important metabolites in this chassis^34^.

In this study, guided by bioinformatic, genomic and transcriptomic analyses, we rationally construct a series of genome-reduced *S. brevitalea* chassis strains for improved heterologous production of Gram-negative proteobacterial NRP/PK natural products. The obtained mutants show improved growth characteristics regarding alleviated autolysis based on deletion of dispensable genes related to transposases, IS elements and prophages and decreased secondary metabolite background by inactivation of native BGCs. The yields of six natural products from Burkholderiales and myxobacteria whose BGCs are heterologously expressed in the genome-reduced *S. brevitalea* chassis are higher than those in the parental DSM 7029 strain. Furthermore, one cryptic BGC from *Chitinimonas koreensis* DSM 17726 is characterized in the chassis strains, with a significant increase in production, leading to the identification of chitinimides, a class of detoxin-like compounds featuring an ureido linkage between two amino acid residues. The constructed chassis strains expand the panel of Gram-negative surrogate hosts and facilitate yield improvements and natural products discovery via heterologous expression of BGCs from Gram-negative proteobacteria, in particular, Burkholderiales and myxobacteria.

## Results

### Determination of deletion targets and genome reduction routes

**Fig. 1:**
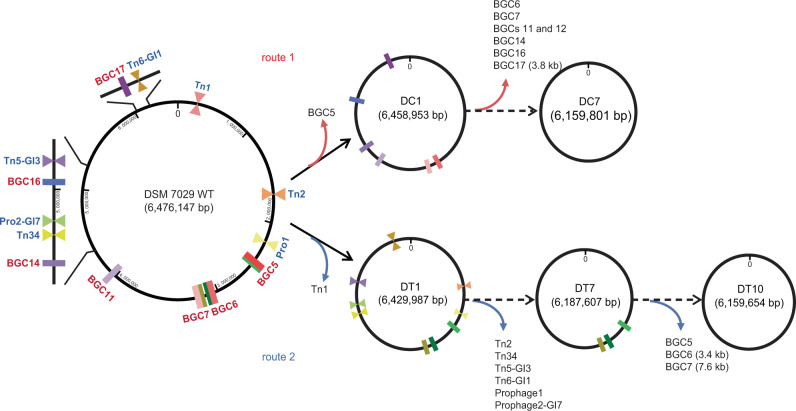
The construction procedure of the genome-reduced mutants of DSM 7029. Circular genome map of the DSM 7029 wild type (WT), targeted deletion regions of biosynthetic gene clusters (BGCs) were marked with bold stubs of different colors and targeted deletion regions of transposases, GIs, prophages were marked with two opposite triangles of different colors on the physical localizations of the chromosome. The route 1 represents the sequential deletions of eight endogenous BGCs, resulting in the seven genome-reduced DSM 7029 strains DC1–DC7. The route 2 represents sequential removals of several transposases, GIs, prophages, and vicinal nonessential genes, leading to the ten genome-reduced DSM 7029 strains DT1–DT10.

The nonessential genes for cellular processes, including endogenous BGCs and genes encoding transposases, IS elements, and phage-related proteins, are targets for deletion, but they are scattered in many regions of the chromosome. We analyzed the genome of the DSM 7029 strain by computational prediction, using antiSMASH to determine BGCs^49^, DEG 10 (Database of Essential Genes) to predict the essential genes and PHAST to annotate prophage sequences^50–52^. There were 17 predicted BGCs in the genome, most of which are silent under laboratory conditions except for that for synthesis of glidobactin (the product of BGC5) and the product of BGC7^46,53^. Large nonribosomal peptide synthetase/polyketide synthase (NRPS/PKS) BGCs were chosen as deletion targets; only the core NRPS/PKS genes were deleted to disrupt the biosynthetic capability, while the precursor biosynthetic genes were retained to maintain the precursor supply. Thus, the first route is to remove the endogenous BGCs (Fig. 1) with the intention of diminishing the native metabolite background and competition for precursors and energy (Supplementary Data 1).

Genome annotation and prediction indicated 44 transposases, 2 prophage-like regions, and 7 genomic islands (GIs) (Supplementary Data 1). Transcriptome analysis shows that genes near transposases or GIs are transcribed at relatively low levels, and they have been predicted to encode hypothetical proteins and be nonessential genes by the DEG 10 database^46,50^. The flagellar machinery is a major metabolic energy consumer, but this kind of surface structure is generally useless in a bioreactor^54^. Most of the transposons, prophages, GIs, flagellar-related regions and their vicinal nonessential genes are focused in seven regions of the chromosome (Fig. 1, Supplementary Data 2). Thus, the second genome reduction route was stepwise deletions of the seven regions, attempting to improve the robustness of engineered strains and, if possible, to alleviate autolysis. To rationally construct the genome-reduced mutants with improved robustness and diminished metabolic backgrounds, the most desired properties from the two deletion routes will be combined to create optimized chassis strains.

### Method and construction of rational genome-reduced DSM 7029 mutants

The selected regions were subjected to markerless deletion by our established Redαβ7029 recombineering combined with the Cre/lox site-specific recombination system^46^. The markerless deletion of the glidobactin BGC (BGC5) was considered the starting point of genome reduction, and the NRPS/PKS encoded genes *glbC-F* (2,677,295–2,694,602, 17.3 kb) were removed by markerless deletion as described in detail in the *Methods* section (Supplementary Fig. 1), leading to the successful construction of the genome-reduced strain designated DC1 with BGC5 eliminated (DC means deletions of clusters, Table 1).

**Table 1:** The detailed information of deleted genomic regions and the constructed genome-reduced DSM 7029 mutant strains via two parallel routes.

Mutants	Genome size (bp)	Deletion regions	Coding product	Deletion size (bp)	Deletion region (start-end)
Wild type	6,476,147 (100%)	–	–	–	–
*route 1*	*Sequential deletions of biosynthetic gene clusters (DC)*				
DC1	6,458,953 (99.7%)	BGC5	NRPS/Type1 PKS (glidobactin)	17,308	2,677,295–2,694,602
DC2	6,383,273 (98.6%)	BGC6	NRPS/trans-AT PKS	75,794	3,068,290–3,144,083
DC3	6,309,096 (97.4%)	BGC7	NRPS	74,291	3,144,084–3,218,374
DC4	6,214,376 (95.9%)	BGCs 11 and 12	NRPS	94,834	3,959,099–4,053,932
DC5	6,189,124 (95.6%)	BGC14	arylpolyene	25,366	4,492,695–4,518,060
DC6	6,163,542 (95.2%)	BGC16	Type1 PKS/NRPS	25,696	5,022,468–5,048,163
DC7	6,159,801 (95.1%)	BGC17 (3.8 kb)	PKS/NRPS	3855	6,065,980–6,069,834
*route 2*	*Sequential deletions of transposons, prophages, genomic islands (DT)*				
DT1	6,429,987 (99.3%)	Tn1	Transposases, hypothetical proteins	46,274	399,555–445,828
DT2	6,399,122 (98.8%)	Tn2	Transposases, hypothetical proteins	30,979	1,869,872–1,900,850
DT3	6,334,697 (97.8%)	Tn34	Transposases, hypothetical proteins	64,539	4,789,725–4,854,263
DT4	6,307,491 (97.4%)	Tn5-GI3	Transposases, flagellar-related proteins	27,328	5,111,402–5,138,729
DT5	6,271,463 (96.8%)	Tn6-GI1	Transposases, hypothetical proteins	36,134	6,161,822–6,197,955
DT6	6,247,380 (96.5%)	Prophage 1	bacteriophage-related proteins	24,197	2,396,164–2,420,360
DT7	6,187,607 (95.5%)	Prophage 2-GI7	bacteriophage-related genes, genomic island region	59,887	4,682,041–4,741,927
DT8	6,170,413 (95.3%)	BGC 5	NRPS/Type1 PKS	17,308	2,677,295–2,694,602
DT9	6,167,154 (95.2%)	BGC 6 (3.4 kb)	NRPS/trans-AT PKS	3373	3,093,895–3,097,267
DT10	6,159,654 (95.1%)	BGC 7 (7.6 kb)	NRPS	7588	3,195,049–3,202,636

**Fig. 2:**
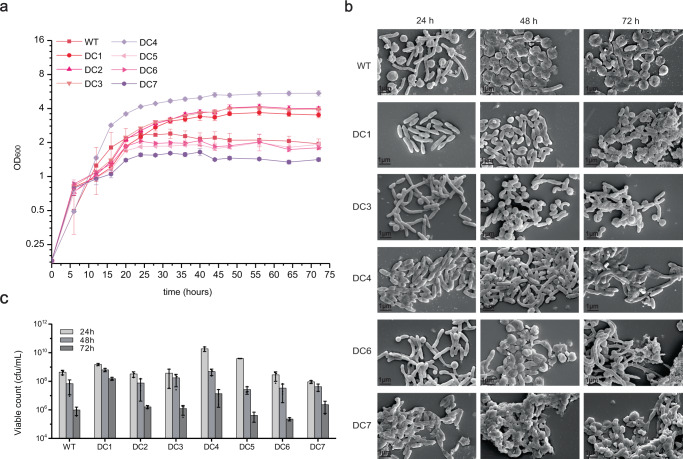
Characterization of the DC series genome-reduced mutants. **a** The growth profiles of the DSM 7029 wild type (WT) and the genome-reduced mutants DC1 to DC7 were measured by culturing in CYMG normal medium after uniform starting OD_600_ values. Data are presented as mean values ± SD. *n* = 3 biologically independent samples. **b** Cell morphology determination by using the field emission scanning electron microscopy (FESEM) of the WT, DC1, DC3, DC4, DC6, and DC7, at 24 h, 48 h, and 72 h, respectively. The experiment was repeated twice independently with similar results. **c** The viable counts of the DSM 7029 and DC1 to DC7 at 24 h, 48 h, and 72 h, respectively. Data are presented as mean values ± SD. *n* = 3 biologically independent samples. cfu, colony-forming number. Source data underlying (**a**, **c**), are provided as a  Source Data file.

Applying multiple deletion cycles, BGCs 6 (glidopeptin, 75.8 kb)^46^, 7 (74.3 kb), 11 and 12 (94.8 kb), 14 (25.4 kb), 16 (25.7 kb), and 17 (3.8 kb, a part of this BGC) were consecutively deleted on the chromosome of the *S. brevitalea* DC1 strain, resulting in the corresponding strains *S. brevitalea* DC2 to DC7, respectively (Table 1, Fig. 1, Supplementary Fig. 2). The DC7 genome was reduced by 317 kb (4.9% of the entire genome) compared with that of the parental *S. brevitalea* DSM 7029 strain. However, DC7 exhibited attenuation in growth features and cellular morphology in some degree (Fig. 2a, b), preventing further genome reduction including from deletion of the remaining BGCs. The combinatorial deletions of multiple BGCs caused some degree of fragility in this strain; thus, we abandoned the first route for further genome reduction.

**Fig. 3:**
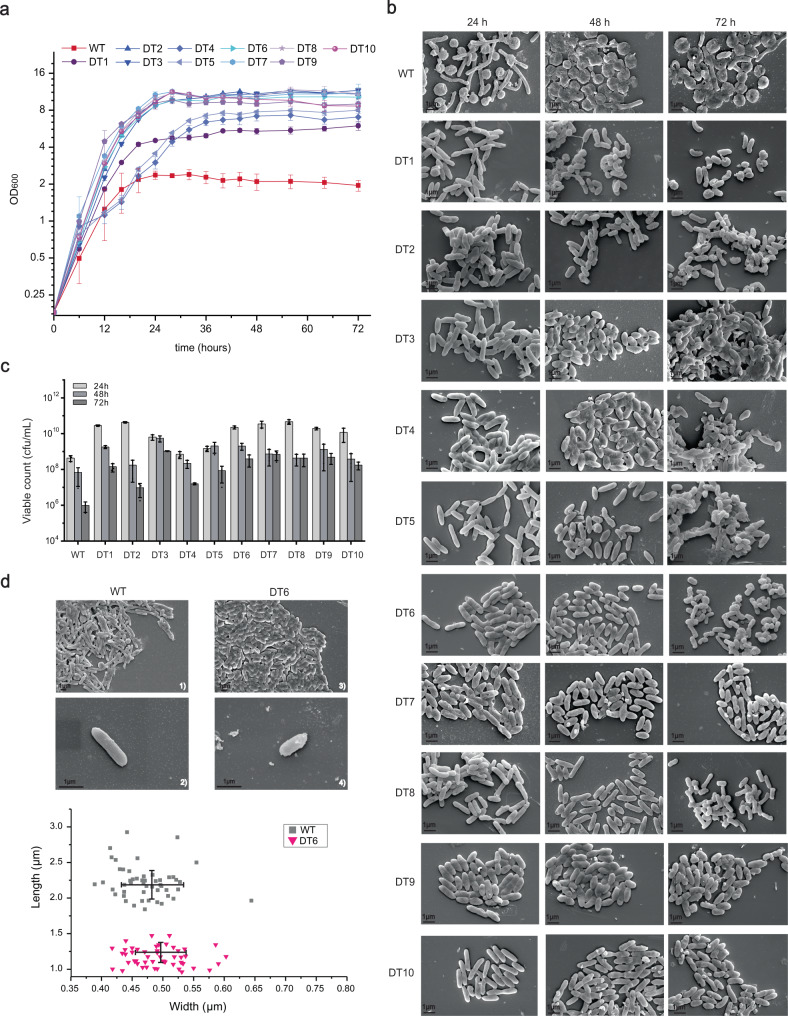
Characterization of the DT series genome-reduced mutants. **a** The growth profiles of the DSM 7029 wild type (WT) and the genome-reduced mutants DT1 to DT10 were measured by culturing in CYMG normal medium after uniform starting OD_600_ values. Data are presented as mean values ± SD. *n* = 3 biologically independent samples. **b** Cell morphology determination by using FESEM of WT, DT1 to DT10, at 24 h, 48 h, and 72 h, respectively. The experiment was repeated twice independently with similar results. **c** The viable counts of the WT and the DT1 to DT10 mutants at 24 h, 48 h, and 72 h, respectively. Data are presented as mean values ± SD. *n* = 3 biologically independent samples. **d** Cell morphology determination of WT and DT6 at 16 h using the FESEM, and the cell-size of WT and DT6 mutant were illustrated in the scatter diagram below by randomly measuring length and width of 50 single cells. Data are presented as mean values ± SD. *n* = 50 biologically independent samples. cfu colony-forming number. Source data underlying (**a**, **c**, **d**) are provided as a Source Data file.

In the second route, we sequentially deleted the seven regions that were rich in transposons, prophages and GIs to obtain strains *S. brevitalea* DT1 to DT7 (DT means deletions of transposons, etc.), respectively (Table 1, Fig. 1, Supplementary Fig. 2). In DT7, a total of 287 kb of genomic regions harboring 23 transposases, 2 prophage-like regions, 3 GIs, and 103 other dispensable genes without predicted function were deleted, reducing the entire genome by 4.5%. All the DT series strains showed improved growth characteristics, especially DT2 to DT7, which exhibited significantly optimized growth properties (Fig. 3a). Subsequently, BGC 5 (17.3 kb), partial BGC 6 (3.4 kb) and BGC 7 (7.6 kb) were successively deleted from DT7 to lessen the secondary metabolite background because BGC5 and BGC7 are constitutively expressed in DSM 7029^46^, creating DT8 to DT10, respectively (Table 1, Fig. 1). Small-sized deletion of BGCs prevents the development of fragile cells, in contrast with the large deletion of BGCs in the DC series strains. Ultimately, we obtained the DT10 with a 4.9% deletion of the whole genome, enhanced properties and a relatively clean metabolic background (Table 1, Fig. 1).

To remove the recombinase expression plasmid to generate plasmid-free genome-reduced mutants (DC1–DC3, DC6, and DT6–DT10) for tests of absolute electroporation efficiency, the plasmid was substituted with the plasmid pBBR1-SacB-apra by means of competitive replication of identical replicons. Then the competitive plasmid was cured via SacB counterselection as described in the “Methods” section.

### Characterization of genome-reduced DSM 7029 strains

The growth profiles of all constructed genome-reduced strains and WT *S. brevitalea* DSM 7029 determined in defined CYMG medium were different, and the DC series mutants (DC1 to DC7) exhibited unusual growth curves without typical exponential and stationary phases. DC1 to DC4 showed slightly better cell growth than the WT strain DSM 7029, while DC5 to DC7 showed a cumulatively defective growth due to the sequential deletions of BGC14, BGC16, and BGC17 (Fig. 2a). The combinatorial deletions of BGCs simplified the metabolic profile but caused some fragility and autolysis, as shown in the cell morphology assays of DC1, DC3, DC4, DC6, and DC7 conducted by using field emission scanning electron microscopy (FESEM), which indicated that the actual cell fragility of the DC6 and DC7 mutants occurred before the first 24 h of cultivation compared with the representative healthy and viable cells of DC1 at 24 h (Fig. 2b). Consistent with the growth characteristics, the live cells number of the DC6 after 72 h of cultivation was decreased compared to those of parental DSM 7029 and there was no increase in the number of viable cells of DC7 than WT strain, affecting their biotechnological performance (Fig. 2c).

The growth profiles of the DT series strain (DT1 to DT10) revealed improved growth with the maximum OD_600_ reaching 10–12, while DT1, DT4, and DT5 had slower growth rates and lower biomass than the other DT mutants (Fig. 3a). In view of the evident discrepancy in cell growth between the DT mutants and the parental strain, we compared their cell morphologies at 24 h, 48 h, and 72 h by using FESEM, respectively (Fig. 3b). DT1 exhibited slightly better growth than the WT, while DT2 to DT5 harbored exhibited relatively delayed autolysis but severely defective cell morphology at 72 h. Intriguingly, in contrast with the cell autolysis of the WT that occurred from 24 h, the DT6 to DT10 mutants exhibited intact cell shapes without detectable lysis (up to 72-h cultivation) after removals of prophage-related genes, which was further demonstrated by viable counts, with almost two orders of magnitude more live cells in DT6 to DT10 than in the DSM 7029 WT after 72 h of batch cultivation (Fig. 3c).

Moreover, cell-size variation in genome-reduced DT mutants was observed using FESEM (Fig. 3d). We quantitatively measured the cell size of DT6 and DSM 7029 WT after 16 h of cultivation because the WT cells at 16 h appeared relatively intact. The cell shape of DT6 became shorter, at ~1.19 μm long and ~0.49 μm wide on average, and the cell length was nearly the half that of the WT (~2.22 μm), while the width was slightly changed (~0.47 μm). Previous studies have shown that a smaller cell size of microbes enabled accelerated exchange of nutrients and metabolites inside and outside the cells, resulting in faster cell growth^55^. Thus, we assumed that the improved growth rate in the DT series emerged along with the smaller cell size.

The reason for autolysis in DSM 7029 is possibly the tight regulation and/or expression of lytic enzymes. The quorum sensing (QS) system and related regulators can modulate several cellular processes in diverse bacteria, such as cell growth and autolysis, in a cell density-dependent manner^38,56–59^. To explore the potential correlation between autolysis and the removed genes, we conducted single inactivation of sixteen hypothetical regulatory genes and putative lytic genes located in the deletion regions (Supplementary Fig. 3a), which have relatively high transcriptional levels, to investigate specific genes responsible for regulating cell growth and cell autolysis. Notably, the DSM 7029 WT harboring the recombinase expression plasmid exhibited faster growth and better cell activity than the original WT strain DSM 7029, with a maximum OD_600_ of ~8 (Supplementary Fig. 3b). The probable reason is that the multicopy replicable plasmid carrying this recombinase pair was implicated in the replication of genomic DNA, which to some extent optimized cell growth at the onset of liquid culturing^60^. However, this strain also showed similar cell autolysis after 48 h (Supplementary Fig. 3c), the trend of which was consistent with the aforementioned cell growth characterizations (Figs. 2b, 3b). Single-gene inactivation experiments of genes encoding the TetR/AcrA family (AAW51_RS20450) and LysR transcriptional proteins (AAW51_RS26400) showed the promotion of cell growth with a maximum OD_600_ of 10, but cell autolysis still occurred after 48 h (Supplementary Fig. 3b, c), suggesting that these proteins are negative regulators of cell growth and biomass. Cell autolysis was delayed significantly in the single mutants with deletion of the lysozyme gene (AAW51_RS19980) in the prophage region (Table 1 and Supplementary Data 2) and the XRE family transcriptional protein gene (AAW51_RS26360) in GI 1 (Table 1 and Supplementary Data 2), but these strains exhibited depressed growth and biomass compared with those of the DSM 7029 WT (Supplementary Fig. 3b, c), indicating that the lysozyme and XRE family transcriptional protein promote the cell autolysis. Thus, combinatorial deletions of regions containing the aforementioned regulatory and lysozyme genes may lead to improvement in cell growth with alleviation of autolysis in DT mutants. However, the specific regulatory mechanism still needs to be investigated.

Thus, the genome reduction in DT series made the cells smaller and delayed or even eliminated the autolysis of cells, laying the groundwork for the industrial fermentation production of valuable products using these chassis. The genome reduction strategy harnessed in this strain can also be applied for the optimization of other chassis, in particular for the alleviation of early autolysis and growth improvement.

**Fig. 4:**
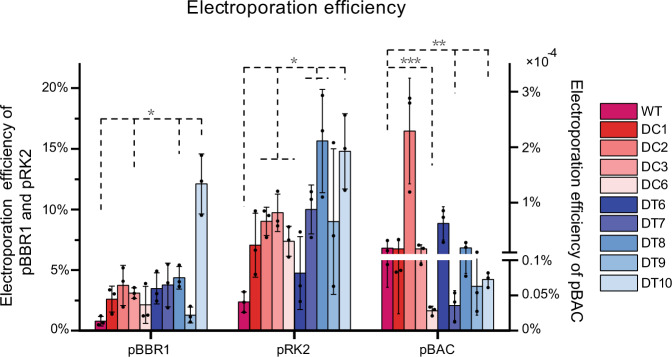
Electroporation efficiency comparison. The absolute electroporation efficiency of the WT and the DT6 to DT10 mutants were calculated with two small multicopy plasmids pBBR1 and pRK2 replicon (the left *Y* axis) and a 99 kb pBAC plasmid carrying an *oriT-tnpA-IR* transposable element and a rhizoxin BGC (the right *Y* axis). Data are presented as mean values ± SD. *n* = 3 biologically independent samples. *P* values were determined by two-tailed unpaired *t* test. *p < 0.05; **p < 0.01; ***p < 0.001. Source data and the exact *p* values are provided as a Source Data file.

The abilities of chassis to accept and uptake exogenous DNAs are important, particularly for chassis expressing large complex BGCs. A previous study of genome-reduced *E. coli* showed emergent properties, including a marked increase in electroporation efficiency with both large single-copy and small multicopy plasmids compared with that of WT *E. coli* strains^24^. We compared the absolute electroporation efficiency of plasmid-free genome-reduced mutants and WT DSM 7029 (Fig. 4). Two small multicopy plasmids (pBBR1-SacB-apra, 6 kb and pRK2-apra-cm, 3.8 kb), and pBAC-apra-TnpA-rhizoxin (99 kb), a bacterial artificial chromosome containing a 92 kb rhizoxin BGC^61^ and an *oriT-tnpA-IR* transposable element^62^, were used in this experiment. The absolute electroporation efficiencies of the pBBR1 replicon plasmids in the DC mutants were on average three times higher than that of the parental strain, and that of DT10 mutants was sixteen times higher than that of the DSM 7029 WT. For thr pRK2 replicon plasmid, the electroporation efficiencies in the DC and DT mutants were higher (1- to 4- fold) than that in the DSM 7029 WT. For pBAC, DC2, DT6, and DT8, with the two deletion routes, had an increased capability to accept foreign DNA. The moderate genome reduction of endogenous BGCs or dispensable genes, e.g., transposases, flagella-related genes, and IS elements, could to some degree increase the ability of cells to take up and carry exogenous DNAs. The intrinsic mechanisms need to be investigated with more in-depth experiments.

Three antibiotics kanamycin (Km), apramycin (Apra), and gentamicin (Genta) are currently available for selection in DSM 7029, impeding the heterologous expression of BGCs carrying other resistance markers. We performed a quantitative assay of the minimum inhibitory concentrations (MICs) of four antibiotics frequently used in genetic engineering, ampicillin (Amp), hygromycin (Hyg), chloramphenicol (Cm) and tetracycline (Tet), in the genome-reduced strains DT6 to DT10 and the WT DSM 7029 (Supplementary Table 2). The results indicated that DT series mutants had increased sensitivity to Amp and Hyg as well as Genta and Apra but slightly elevated resistance to Cm and Tet, suggesting that apart from Km, Apra, and Genta, Amp could also be used as a resistance selection marker in genome-reduced strains for heterologous expression of BGCs.

Based on the comprehensive comparison of growth characteristics, electroporation efficiencies and metabolic backgrounds, which are essential traits of chassis cells applied in biotechnology and industrial production, the genome-reduced mutants DT6–DT10 were selected for heterologous production of natural products to screen promising heterologous hosts.

### Heterologous expression of BGCs in genome-reduced DSM 7029 strains

To compare the productivity of the genome-reduced *S. brevitalea* chassis for the heterologous expression of compounds, the BGCs of six proteobacterium-derived natural products, namely, the NRPs rhizomide and holrhizin, the hybrid NRPS/*trans*-AT PKS-derived rhizoxin from *Paraburkholderia rhizoxinica* HKI 454^46,48,61^, the NRP icosalide from *Burkholderia gladioli* ATCC 10248^63,64^, the hybrid NRP/PK epothilone from the myxobacterium *Sorangium cellulosum* So ce90^33,34^, and the NRP siderophore myxochelin from *Myxococcus xanthus* DK 1622^65^, were transferred and integrated into the target chromosome of the genome-reduced strains DT6–DT10 and the DSM 7029 WT, as well as another two commonly used Gram-negative bacterial chassis, *E. coli* GB05-MtaA, the *E. coli* GB2005 strain harboring the phosphopantheteine transferase MtaA from *Stigmatella aurantiaca*^66^, and *P. putida* KT2440^67^. The phiC31 attB loci for site-specific integration of foreign DNA were introduced into the chromosomes of the DSM 7029 strains, *E. coli* GB05-MtaA and *P. putida* KT2440 via Red/ET recombineering in advance. A single-copy BGC with the *oriT-attP-phiC31* cassette was then targeted and integrated into the chromosome to prevent the position effect on production. The correct transformants were verified by colony PCR and cultivated in the CYMG liquid medium with appropriate antibiotics for metabolic analysis (see the *Methods* section).

**Fig. 5:**
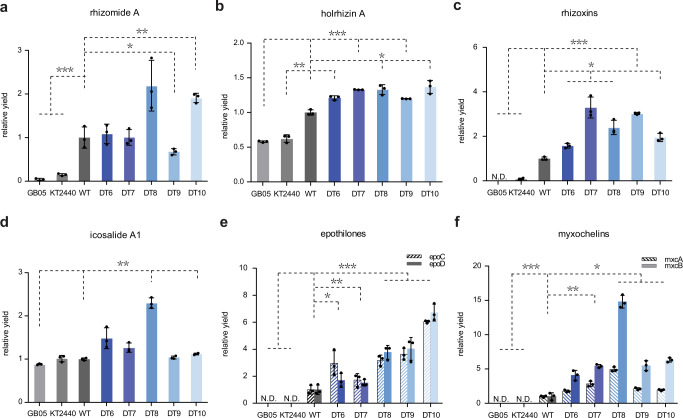
The relative yields of heterologously expressed six natural products. Relative yields of heterologously expressed rhizomide A (**a**), holrhizin A (**b**), rhizoxins (**c**), icosalide A (**d**), and epothilones (**e**) and myxochelins (**f**) in *E. coli* GB05-MtaA (GB05), *P. putida* KT2440 (KT2440), DSM 7029 wild type (WT), and genome-reduced mutants (DTs). The BGCs were integrated into the genome by phiC31 site-specific recombination, respectively. Data are presented as mean values ± SD. *n* = 3 biologically independent samples. *P* values were determined by two-tailed unpaired *t* test. *p < 0.05; **p < 0.01; ***p < 0.001. Source data and the exact *p* values are provided as a Source Data file.

We found that the production of rhizomide A in the DT8 mutant with deletions of nonessential genes and the glidobactin BGC was increased to 2-fold that in the DSM 7029 WT and other mutant strains, which was at least 50-fold and 15-fold higher than that in GB05-MtaA and KT2440, respectively (Fig. 5a). With respect to the yields of holrhizin A in genome-reduced strains, there were certain degrees of increase compared to that in the DSM 7029 WT (19–36%), particularly for the highest yield obtained in the DT10 mutant with the maximum genomic region deletion in the DT series, which exhibited conspicuous improvement in yield compared to that in GB05-MtaA and KT2440 (136% and 119%) (Fig. 5b). The production levels of total rhizoxin derivatives in the genome-reduced DT7 and DT9 chassis were increased to 3.2-fold and 3-fold that in the WT strain, respectively, and the maximum yields of rhizoxins were obtained in the DT7 strain. In contrast, the GB05-MtaA could not produce rhizoxins at all, while only trace amounts of rhizoxins were detected in KT2440, which represented a 35-fold decrease in production yield compared with that for the DT7 strain (Fig. 5c). The icosalide A1 yield from the genome-reduced strain DT8 was improved ~1.6- to 2.2-fold compared with that of the DSM 7029 WT, GB05-MtaA, and KT2440 (Fig. 5d). To compare the yields of epothilones, a plasmid containing rare tRNAs and the methylmalonyl-CoA biosynthetic pathway was also electroporated after site-specific integration of epothilone BGC^34^. The defined CYMG liquid medium used for fermentation was also supplemented with 100 mg/L methylmalonic acid to facilitate the biosynthesis of epothilones. Epothilones C and D were not produced in either GB05-MtaA or KT2440. The production yields of epothilones C and D in the DT7 to DT10 mutants showed gradual improvements of one- to fivefold compared to those in the DSM 7029 WT, especially for the production yield of epothilone D in DT10, which was fivefold higher than that in the parental DSM 7029 strain (Fig. 5e). The DT mutants showed improved growth features and enhanced epothilone yields, facilitating potential industrial fermentation. Another myxobacterial myxochelin BGC was also successfully expressed in *S. brevitalea* mutants and the WT but not in the other two chassis strains, and the observed production yields of myxochelins A and B in the DT8 mutant were dramatically higher (3.9- and 13.8-fold) than those in the DSM 7029 WT and other mutants (Fig. 5f). These results demonstrated the feasibility and improved robustness of these genome-reduced DSM 7029 mutants for heterologous expression of PKS/NRPS natural products from myxobacteria and Burkholderiales compared to that of the DSM 7029 WT and two other well-accredited chassis.

Overall, the optimal chassis cell with desired properties and relatively high production of heterologous natural products was a panel of DT8 and DT10, which had less metabolic background, and these were used for the subsequent genome mining of compounds via heterologous expression of cryptic BGCs from other proteobacteria.

### Discovery of natural products using genome-reduced DSM 7029 strain

**Fig. 6:**
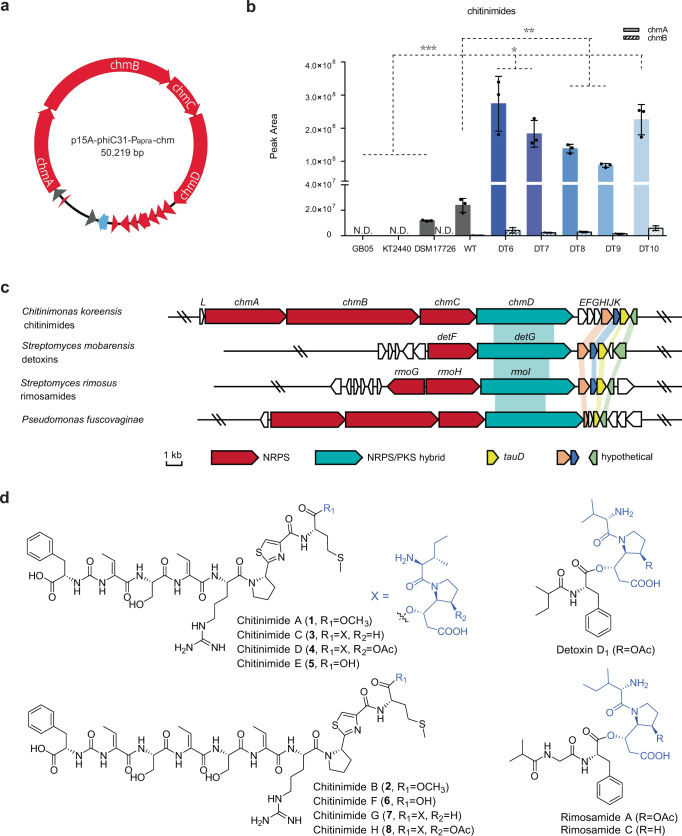
Discovery of chitinimides by heterologous expression of *chm* BGC in genome-reduced chassis. **a** Construct of p15A-phiC31-Papra-chm with p15A replicon (blue), *oriT-attP-phiC31* cassette (gray), and *chm* BGC (red) was under the control of P_apra_ constitutive promoter (gray arrow upstream of the *chmA*). **b** Yield comparison of chitinimides (A and B) in *E. coli* GB05-MtaA (GB05), *P. putida* KT2440 (KT2440), native producer *C. koreensis* DSM 17726 (DSM 17726), DSM 7029 wild type (WT) and DT6–DT10 mutants. Data are presented as mean values ± SD. *n* = 3 biologically independent samples. *P* values were determined by two-tailed unpaired *t* test. *p < 0.05; ** *p* < 0.01; *** *p* < 0.001. Source data and the exact *p* values are provided as a Source Data file. **c** The bioinformatics analysis of *chm* BGC from *C. koreensis* DSM 17726. The *chm* BGC (top) shares many ORFs that of high similarity with the detoxin and rimosamide BGCs in Streptomyces spp. and an undefined BGC in *Pseudomonas* sp. Homologous ORFs are marked with identical color. **d** The structures of chitinimide A-H (**1**–**8**), and the structures of detoxin and rhimosamides.

To harness the genome-reduced *S. brevitalea* chassis for genome mining of natural products via heterologous expression, we cloned, engineered and transferred a cryptic *chm* BGC from the Gram-negative bacterium *Chitinimonas koreensis* DSM 17726 (β-proteobacteria) into the parental DSM 7029 WT strain and the genome-reduced strains DT6 to DT10^68^. The *chm* BGC was found by antiSMASH analysis of the whole shotgun-sequenced genome (NZ_ATZZ00000000.1)^69^ but was incomplete. We resequenced and reassembled the whole genome to obtain this 43.7 kb complete *chm* BGC (GenBank: MW160162). The *chm* BGC belongs to a family of detoxin/rimosamide BGCs featuring a hybrid NRPS/PKS gene (*detG*/*rmoI*) and a *tauD*-like *(detJ*/*rmoL*) gene. This BGC family is widely distributed in actinobacterial genomes, and their products detoxins and rimosamides are rare secondary metabolites displaying anti-antibiotic activity from *Streptomyces* sp^70,71^. The *chm* BGC (*chmA*-*chmL*) not only consists of the characteristic core (hybrid NRPS/PKS gene *chmD* and *tauD*-like gene *ChmJ*) which exists in all known detoxin and rimosamide BGCs but also harbors three more NRPS genes *chmA*, *chmB* and *chmC* encoding eight modules expected to produce a much longer peptidyl backbone than detoxin and rimosamide (Fig. 6c, Supplementary Table 3). The *chm* BGC was cloned from the genome of DSM 17726 via direct cloning mediated by ExoCET^72^. A potent constitutive P_apra_ promoter was then inserted in front of the first biosynthetic gene of the *chm* operon to drive the whole BGC^34^, and the cassette *oriT-attP-phiC31* was also added into the *chm* expression vector to transfer the BGC into the chromosome of the heterologous host via site-specific integration^62^. The resulting BGC was electroporated and integrated into the genome of the WT DSM 7029, genome-reduced chassis DT6 to DT10, and two Gram-negative bacterial chassis, *E. coli* GB05-MtaA and *P. putida* KT2440, for heterologous expression to investigate its products (Fig. 6a).

The LC–MS analysis showed that GB05-MtaA and KT2440 lacked the capability for heterologous expression of *chm* BGC. The genome-reduced DT6–DT10 carrying engineered *chm* BGC produced several evident peaks, whereas only two small peaks were present in the DSM 7029 WT containing the *chm* BGC, but the peaks were completely absent in the negative control. Especially for the main product chitinimide A (**1**), the production yield in the genome-reduced DT6–DT10 was fivefold to one order of magnitude higher than that of the WT DSM 7029 and the native producer *C. koreensis* DSM 17726. For chitinimide B (**2**), the production yield in the DT10 mutant was 13.7-fold higher than that in WT DSM 7029, while no chitinimide B was produced in DSM 17726 (Fig. 6b). The products of *chm* BGCs, chitinimides, showed clearly improved production in the genome-reduced mutants, indicating that these strains are superior chassis than the DSM 7029 WT and two other widely used Gram-negative chassis for heterologous expression of many exogenous BGCs from proteobacteria to discover natural products.

We isolated and identified four main products, chitinimides A–D (**1**–**4**), by high-resolution electrospray ionization mass spectroscopy (HRESIMS), nuclear magnetic resonance (NMR) spectroscopy and chemical derivation (Fig. 6d). Chitinimide A (**1**) was isolated as a colorless oil with the molecular formula established as C_41_H_57_N_11_O_11_S_2_ based on HRESIMS (*m/z* 944.3776 [M + H]^+^, *calcd* 944.3753), together with its ^1^H and ^13^C NMR spectra data (Fig. 6d, Supplementary Table 4, Supplementary Figs. 16, 17), which indicated 19 degrees of unsaturation. The 1D NMR and distortionless enhancement by polarization transfer (DEPT) (Supplementary Table 4, Supplementary Figs. 18, 19) combined with the substrate specificity predicted by the adenylation (A) domain (Supplementary Fig. 4) revealed the existence of one Met, one Arg, two 2,3-dehydrobutyric acids (Dhb), one Ser, one Phe, one tetrahydropyrrole (THP) and one 2,4-disubstrituated thiazole, which was further confirmed by ^1^H-^1^H correlation spectroscopy (COSY) and heteronuclear multiple bond (HMBC) correlations (Supplementary Figs. 6, 20–22). These units were connected to form a linear polypeptide on the basis of the HMBC correlations (Supplementary Fig. 6). In addition, the remaining methoxy group (C-1′, *δ*_H_/*δ*_C_ 3.65, 52.1) and ureido moiety (C-31, *δ*_C_ 155.6) were located at C-1 and between C-28 and C-33, respectively, evidenced by the HMBC correlations from H-1′ to C-1 and from 28/33-NH to C-32. Chitinimide B (**2**) was also isolated as a colorless oil, having a molecular formula of C_48_H_67_N_13_O_14_S_2_, as deduced from the HRESIMS (*m/z* 1114.4467 [M + H]^+^, *calcd* 1114.4445). Comparison of NMR spectroscopic data of **1** and **2** (Fig. 6d, Supplementary Table 4, Supplementary Figs. 23–28) demonstrated that **2** differed from **1** only by the presence of an additional Ser and Dhb. The locations of the additional Ser and Dhb were supported by HMBC correlations (Supplementary Fig. 6). The absolute configurations of the amino acid residues of **1** and **2** were confirmed by HPLC-MS analysis of the 1-fluoro-2-4-dinitrophenyl-5-L-alanine amide (FDAA) derivatives according to Marfey’s method (Supplementary Table 6)^73,74^. The remaining absolute configuration of C-10 was designated *S* by bioinformatic analysis, which indicated that the NRPS *ChmB* encoded an L-Pro (Supplementary Fig. 6).

Chitinimide C (**3**) and chitinimide D (**4**) were obtained as mixtures at a ratio of approximately 1:2 due to their nearly identical retention times and peak tailings. Their molecular formulas were defined as C_53_H_77_N_13_O_14_S_2_ (*m/z* 1184.5240 [M + H]^+^) and C_55_H_79_N_13_O_16_S_2_ (*m/z* 1241.5286 [M + H]^+^) according to the HRESIMS data, respectively. The NMR spectra of the mixture of **3** and **4** were analyzed, and comparison of the NMR data (Fig. 6d, Supplementary Table 5, Supplementary Figs. 29, 30) with that for **1** indicated that **3** and **4** also shared the same octapeptide skeleton, except for an additional part of X (Fig. 6d) instead of a methoxy group. The structures of X in **3** and **4** were proposed to be the same as those of rimosamides C and A considering the high level of sequence similarity of their PKS module (Supplementary Fig. 4), which was also consistent with their molecular weights and supported by correlations from 2D NMR spectra (Supplementary Fig. 6, Supplementary Figs. 31–34). The two units were connected via an ester bond, as indicated by the HMBC cross-peaks between H-3′ and C-1 (Supplementary Fig. 6). The absolute configurations of the amino acid residues in **3** and **4** were confirmed by using Marfey’s method (Supplementary Table 6). The high sequence similarity between ChmD, DetG and RmoI, as well as bioinformatic analysis of the condensation (C) and ketoreductase (KR) domains of ChmD (Supplementary Fig. 4) suggested that the stereochemistry of C-3′, C-4′ and C-5′ was identical to that of the compounds described as 3′ *S*, 4′ *R*, and 5′ *R*. The structures of **1**–**4** were further confirmed by HRESIMS/MS (Fig. 6d, Supplementary Figs. 8–11). The structures of chitinimide E-H (**5**–**8**) were deduced via comparative HRESIMS/MS fragment analysis (Fig. 6d, Supplementary Figs. 12–15).

The potential biological function and bioactivity of chitinimides were also briefly investigated. They also exhibited antagonistic activity against blasticidin S in *Bacillus cereus* (Supplementary Fig. 7) as shown for detoxins and rimosamides in previous reports^70,75^. The bioactivity of chitinimides was then screened by several assays, chitinimide B (**2**) showed weak antiproliferative activity against the human cancer cell lines Kasumi and MDA-MB-231, with IC_50_ values ranging from 14 to 22 μM (Supplementary Table 7), and chitinimide A (**1**) displayed weak in vitro inhibitory activity (IC_50_ = 6.01 μg/mL) against the cysteine protease cathepsin B, a potential drug target for several diseases^76,77^, via an enzymatic activity assay (Supplementary Table 8).

The heterologous production yield of chitinimide A in the genome-reduced mutants was increased five- to tenfold compared with that of the native producer DSM 17726 and the DSM 7029 WT, simplifying the isolation and purification processes of target products. Thus, based on the relative yield comparison to DSM 7029 WT and other well-accredited Gram-negative chassis strains, the genome-streamlined DT series mutants were proven to be the optimal chassis cells for heterologous expression and biosynthetic studies, which could accelerate the course of genome mining.

## Discussion

Here, we constructed a series of DSM 7029 mutants with multiple deletions via two parallel routes. In the first route with several endogenous secondary metabolite BGCs eliminated, the mutants exhibited a decrease in certain growth parameters to some extent, suggesting that these BGCs may have certain endogenous functions in cell growth. The DC7 mutant exhibited more impaired growth features and cellular morphology than WT DSM 7029 (Fig. 2), which may be due to the cumulative effect of the sequential deletions of BGC14, BGC15, and BGC17 from the DC4, a mutant displaying a better growth profile than WT DSM 7029.

In the second route, with a number of transposases, GIs and other dispensable genes deleted, the DT1–DT10 mutants showed classical growth curves with typical exponential and stable phases, and enhanced cell growth and biomass, and shorter and smaller cells, and the early autolysis of cells of DT6 to DT10 was inhibited (Fig. 3). The acyl-homoserine lactone (AHL)-mediated QS system, which is widely distributed in Burkholderiales strains, has been demonstrated to play an important role in multiple cellular processes, e.g., restriction of nutrient acquisition and maintenance of metabolic homeostasis, production of intra- and extracellular products, control of virulence, motility, and efflux pumps^56,57,78,79^. The removal of putative QS-related regulators in DSM 7029, e.g., a TetR/AcrR family transcriptional regulator (AAW51_RS20450) or LysR family transcriptional regulator (AAW51_RS26400), to some extent enhanced the cell growth in log phase (Supplementary Fig. 3a, b). A previous report demonstrated that deletion of multiple lytic genes, such as prophages in Gram-positive bacteria, e.g., *Bacillus subtilis*, can prevent cell lysis and enhance biomass^80^. A recent study showed that the salicylic acid interferes with the regulatory *agar* QS system in pathogenic *Staphylococcus aureus*, consequently negatively affecting autolysis^58^. The delay of autolysis in DT mutants of DSM 7029 was probably due to the deletion of lytic genes or their regulatory genes during the genome reduction procedure, e.g., lysozyme or XRE family transcriptional regulator-coding genes (Supplementary Fig. 3). The improvement in sensitivity to certain antibiotics and negative regulation of autolysis in DSM 7029 DT series mutants may also be related to the perturbation of the intrinsic QS system, which needs to be explored with additional experiments in the future.

The marked change in cell size of the DT strains may also be correlated with an improved growth rate and biomass^55^, which is comprehensively controlled by several complex processes, and the subtle variations in each process, e.g., central carbon metabolism, cell cycle progression, or cell envelope biogenesis, can considerably implicate cell size^81^. However, no universal model could be utilized to determine how these separate processes are functionally coordinated, and the molecular basis and regulation of bacterial cell size control remain imperfectly understood^81^. The smaller cell size and possible increase in membrane homeostasis that emerged in these mutants may provide better robustness and adaptability in atypical environments during industrial production, and this has been demonstrated in several studies focused on improving the performance of cell factories^82^. The alleviation of autolysis observed in the DT series mutants will greatly facilitate industrial bioprocessing, but the molecular mechanism controlling autolysis and the relationship among growth, cell size and autolysis in this strain is thought-provoking and still need to be investigated through additional experiments in the future.

The recently described GI-free *P. putida* genome-reduced strain KTU-U13 exceeded the parental strain KTU in many physiological traits, e.g., plasmid transformation efficiency and heterologous protein expression capacity^31^. Our study also underscored that the DT mutants of DSM 7029 may to some extent exhibit increased expression of exogenous DNA, therefore upgrading the heterologous production of exogenous secondary metabolic pathways in mutant strains with reduced metabolic backgrounds and improved growth status.

The versatile heterologous host traits of the DSM 7029 mutants were further demonstrated via improved yields of diverse natural products from proteobacteria and successful expression of the cryptic *chm* BGC from *C. koreensis*. The production yields of compounds of the same natural product BGC in several genome-reduced mutants showed differences, possibly because of the cumulative effect of multiple deletions affecting the biomass or regulatory or metabolic networks. Except for the conserved core of the detoxin/rimosamide BGC for biosynthesis of the X part, up to three adenylation (A) domains in actinobacteria form the remaining short linear peptide of detoxin/rimosamide^71^, while seven A domains in a putative BGC from *Pseudomonas fuscovaginae* strain LMG 2158 (GenBank: LT629972.1) and eight A domains in the *chm* BGC from *C. koreensis* form longer linear peptides (Supplementary Fig. 4). We assumed that the highly conserved core structure of the X part was not limited to actinobacteria but distributed in many phylogenetically distant bacteria via gene cluster horizontal transfer, which might be related to their defense mechanism against other organisms^70^. Our research extends the detoxin/rimosamide BGC family to Gram-negative proteobacteria, and their anti-antibiotic activity suggests the complex chemical ecology of these microorganisms. The exact biological function and activity of chitinimides need to be further scrutinized via additional experiments.

Overall, we report that the Burkholderiales strain that underwent rational genome reduction exhibited dramatically improved properties for growth and increased efficiency of heterologous expression of bacterial natural products, which will facilitate the genome mining of cryptic BGCs derived from proteobacteria and improve the production yields of compounds of interest. Thus, these mutants extend the panel of Gram-negative chassis for heterologous expression of natural product BGCs from proteobacteria. Our study suggest that the combinatorial deletions of transposases, IS elements, prophages and dispensable genes in bacteria could be a feasible strategy to construct optimal chassis as robust cell factories.

## Methods

### Bacterial strains and reagents

Strains, plasmids and mutants used and created in this study, and primers used in this study were listed in Supplementary Data 3. PCRs performed with PrimeSTAR Max DNA Polymerase (Takara, cat. -no. R045B) and further purification of dsDNA with Agarose gel DNA recovery kit (TIANGEN, cat. -no. DP214-03) according to manufacturer’s instructions. Construction of plasmids in this study were performed via recombineering in *E. coli* GB05-dir for LLHR or in *E. coli* GB08-red for linear plus circular homologous recombination (LCHR)^46^. The procedure of direct cloning carried out in *E. coli* GB05RedTrfA harboring the pSC101-BAD-ETgA-tet plasmid and Red/ET recombineering occurring in *E. coli* GB08-red via LLHR and LCHR were shown in Supplementary Fig. 5^10,62,66,83,84^. Antibiotics were purchased from Sangon (Shanghai) Co., LTD., in China. The restriction enzymes and DNA markers were purchased from New England Biolabs. All *E. coli* strains were grown on Luria broth (LB) medium for propagation at 37 °C. The antibiotic concentrations used for resistance selection of *E. coli* strains on LB agar plates or liquid medium were as follows: kanamycin (15 or 10 µg/mL), gentamicin (4 or 2 µg/mL), and apramycin (20 or 10 µg/mL). *S. brevitalea* DSM 7029 strains were grown on CYMG medium for propagation, growth rate determination, and electroporation efficiency determination at 30 °C^46^. The antibiotic concentrations used for resistance selection of DSM 7029 strains on CYMG agar plates or liquid medium were as follows: kanamycin (30 or 20 µg/mL), gentamicin (25 or 20 µg/mL), and apramycin (30 or 20 µg/mL). The *P. putida* KT2440 was grown on Luria broth (LB) medium for propagation at 30 °C. The antibiotic concentrations on LB agar plates or liquid medium were as follows: kanamycin (30 or 20 µg/mL), gentamicin (10 or 5 µg/mL), apramycin (40 or 20 µg/mL), and ampicillin (400 or 200 µg/mL). The transformations of plasmids into *S. brevitalea* DSM 7029 strains were performed by electroporation (*Eppendorf*^*®*^ *Electroporator 2510, 1350V*)^46^. The large constructs were introduced into the specific chromosomal site of *P. putida* with the help of donor strain *E. coli* WM3064 via conjugation^85,86^. *C. koreensis* DSM 17726 strain was purchased from the Deutsche Sammlung von Mikroorganismen und Zellkulturen (DSMZ). For genomic DNA preparation, DSM 17726 was cultured in R2A medium for 12 h at 30 °C^68^.

### Markerless deletion of genome sequence region in DSM 7029

The Redγ-Redαβ7029 recombineering^46^, Cre/loxP site-specific recombination, and SacB counterselection system were combinatorically applied to the sequential deletions of several dispensable regions as shown in Supplementary Fig. 1. At the first place, the target deletion region on the chromosome of DSM 7029 containing Redγ-Redαβ7029 expression plasmid was replaced by the *Genta* selection marker flanked with *lox66*, *lox71* sites, and homology arms (HAs, ~80 bp) mediated by Redγ-Redαβ7029 recombinase via recombineering^46^ (Supplementary Fig. 1a). The correct recombinants were selected on the CYMG agar plate containing kanamycin and gentamicin and further verified by colony PCR with check primers in Supplementary Table 6. Then, the Cre/loxP site-specific recombination mediated by Cre recombinase was implemented to eliminate the *Genta* resistance selection marker, leaving a 34 bp nonrecombinogenic scar sequence *lox72* and 80 bp flanking sequence (Supplementary Fig. 1b). The Cre expression plasmid pRK2-BAD-Cre-apra-SacB was electroporated into the abovementioned correct mutant, and transformants were screened on CYMG agar plate containing kanamycin, gentamicin and apramycin. The single clone was verified with inside check primers of Cre expression plasmid (*sacB-ins-1* and *sacB-ins-2*, Supplementary Data 3). Inoculating correct transformants into 1.3 mL liquid CYMG medium containing kanamycin and apramycin, and transfer 20 µL culture into 1.3 mL fresh medium after 24 h culturing at 30 °C, and the inoculation procedure needed to be repeated for 3–4 times. To induce the expression of Cre site-specific recombinase and efficient elimination of selection marker, 35 µL L-(+)-Arabinose (100 mg/mL) was added with the transferring. After the last transferring, approximate 100 µL culture were three-zone spread on CYMG agar plate containing kanamycin and apramycin, and single clone with successful elimination of *Genta* marker was verified by colony PCR with check primers independently as listed in Supplementary  Data 3. The last step in each round of deletion is the elimination of Cre expression plasmid pRK2-BAD-Cre-Apra-SacB via SacB counterselection (Supplementary Fig. 1c). The verified correct transformant in former step was inoculated in CYMG liquid medium containing kanamycin and 5% sucrose at 30 °C for 24 h, then transfer approximate 20 µL overnight culture into CYMG liquid medium containing kanamycin and 10% sucrose at 30 °C for 24 h, and transfer 20 µL overnight culture into CYMG liquid medium containing kanamycin and 15% sucrose, repeat the above step for 2–3 times at 30 °C for 24 h. After last transferring, approximate 100 µL culture were three-zone spread on CYMG plate containing 30 µg/mL kanamycin and successful elimination of Cre expression plasmid was verified by colony PCR with inside check primers (*sacB-ins-1* and *sacB-ins-2*, Supplementary Data 3) for the operation of next round of deletion.

### Remove of recombinase expression plasmid in genome-reduced strain

The Redγ-Redαβ7029 expression plasmid pBBR1-Rha-RedG-BA_7029-km need to be removed before absolute electroporation efficiency determination. The nine selected genome-reduced mutants, DC1–DC3, DC6, DT6–DT10 with Redγ-Redαβ7029 expression plasmid were inoculated in CYMG liquid medium containing kanamycin, and competitive plasmid with identical origin pBBR1-SacB-apra were electroporated into these mutant strains, and correct transformants were screened on CYMG agar plate containing apramycin and further verified by colony PCR using pBBR1-SacB-apra inside check primers (*sacB-apra-ins-check-1*, *sacB-apra-ins-check-1*, Supplementary Data 3). Then single clone was picked into CYMG liquid medium containing apramycin and sequentially transferred for 2–3 times as mentioned above. After the last transfer, approximate 100 µL culture were three-zone spread on CYMG agar plate containing apramycin with successful removal of Redγ-Redαβ7029 expression plasmid, and transformants were verified by colony PCR with inside check primers (*sacB-apra-ins-check-1*, *sacB-apra-ins-check-1*, Supplementary Data 3). The SacB counterselection was applied to subsequently remove the competitive plasmid pBBR1-SacB-apra as aforementioned.

### Growth characteristic test

Growth profiles of DSM 7029 WT and all constructed genome-reduced mutants in this study (DC1 to DC7, DT1 to DT10) were determined in CYMG normal liquid medium without antibiotic by culturing in 250 mL flasks at 30 °C, 200 rpm. The starting OD_600_ values were uniformed and the OD_600_ value under the UV 600 nm of per sample was measured every 6 h during the first 12 h of cultivation, and every 4 h during 12 to 48 h of cultivation, then every 8 h until 72 h of cultivation. Three parallel trials of every determined sample were set to perform error bar and standard deviation.

### Absolute electroporation efficiency determination

Two small multicopy plasmids and one pBAC artificial bacterial chromosome containing 92-kb rhizoxin gene cluster were used to calculate the absolute electroporation efficiency of DSM 7029 WT and nine selected genome-reduced mutants DC1–DC3, DC6 and DT6–DT10. One of the small plasmid, pRK2-apra-cm, was constructed via LLHR in *E. coli* GB05-dir^46^, while another small plasmid, pBBR1-SacB-apra, was constructed by LCHR in *E. coli* GB08-red based on the plasmid pBBR1-Rha-RedG-BA_7029-km-SacB and the *apra-SacB* cassette flanked with corresponding HAs (with primers in Supplementary Data 3)^62^. The plasmids pBAC-km-phiC31-rhizoxin was constructed before, the *oriT-tnpA-apra* cassette flanked with corresponding HAs was amplified by primers (Supplementary Data 3) using p15A-apra-tnpA-rhizomide as template via PCR and dsDNA purification to construct pBAC-apra-tnpA-rhizoxin by LCHR in *E. coli* GB08-red. The OD_600_ values of DSM 7029 WT and nine selected genome-reduced mutants were uniformed to 3.0 before electroporation, and 1 μg plasmid was electroporated into these DSM 7029 strains, respectively. The electroporation protocol was performed by using room temperature cuvettes (1 mm) and an Eppendorf 2510 electroporator (1300 V)^46^. To calculate the absolute electroporation efficiency of pRK2-apra-cm or pBBR1-SacB-apra, the clone number of DSM 7029 strains harboring pRK2-apra-cm or pBBR1-SacB-apra was counted on CYMG agar plates containing appropriate apramycin and CYMG agar plates with no antibiotics, separately, after appropriate dilution, and the absolute electroporation efficiency were measured via the clone number of the former divided by that of the latter. The DSM 7029 strains with rhizoxin BGC integrated into the genome was screened on CYMG agar plates containing appropriate apramycin, and DSM 7029 strains without any plasmid electroporated into were coated after appropriate dilution on CYMG agar plates without antibiotics, and the absolute electroporation efficiency was measured as abovementioned. Three parallel trials of every determined sample were set to perform error bar and standard deviation.

### Antibiotics minimum inhibitory concentration assay

Seven kinds of antibiotics, including four inapplicable: ampicillin (Amp), hygromycin (Hyg), chloramphenicol (Cm) and tetracycline (Tet), as well as three applicable antibiotics: kanamycin (Km), gentamicin (Genta), and apramycin (Apra), were selected to qualitatively compare the sensitivity of DSM 7029 WT and five selected genome-reduced mutants (DT6 to DT10). The starting OD_600_ values of DSM 7029 strains were uniformed to 0.2 in 2 mL EP tube. For ampicillin, chloramphenicol, tetracycline, kanamycin, gentamicin, and apramycin, the concentrations of antibiotics were gradually diluted by CYMG liquid medium from 128 to 0.0625 μg/mL. For hygromycin, the gradient concentrations were set from 1024 to 0.5 μg/mL. The final OD_600_ values were determined after culturing at 30 °C, 950 rpm for 16 h. The MIC of each antibiotic were determined by unchanged OD_600_ value and viable counts on CYMG agar plates without antibiotics.

### Integration of attachment sites on the choromosome of chassis strains

The Redγ-Redαβ7029 recombineering was utilized to integrate the phiC31 attB site into the chromosome of DSM 7029 WT and DT6 to DT10 genome-reduced mutants^46,62^. The 2789-bp genomic region (from 3,979,777 to 3,982,566 bp) within BGC11 was replaced by *attB-Genta* cassette flanked with *lox66*, *lox71* sites and homology arms (HAs, ~80 bp) mediated by pBBR1-Rha-RedG-BA_7029-km^46^. The correct transformants were selected on the CYMG plate containing gentamicin, and further verified by colony PCR using check primers (*C11-attB-out-1*, *C11-attB-out-2*, Supplementary Data 3) and DNA sequencing. The Red/ET recombineering was utilized to integrate the phiC31 attB site into the chromosome of *E. coli* GB05-MtaA^66^, mediated by pSC101-BAD-gbaA-tet^10^. The Genta antibiotic selection marker in this strain was partially replaced with *attB-km* cassette flanked with *lox66*, *lox71* sites and HAs (~50 bp). The correct transformants were screened on the LB agar plate containing kanamycin, and further verified by colony PCR using check primers (*MtaA-attB-out-1*, *MtaA-attB-out-2*, Supplementary Data 3) and DNA sequencing. The Red/ET recombineering was also used to integrate the phiC31 attB site into the chromosome of *P. putida* KT2440, mediated by pBBR1-Rha-GBAS-km^87^. The 500-bp genomic region (from 4,829,741 to 4,830,241 bp) of *P. putida* KT2440 was replaced with Genta selection marker flanked with *lox66*, *lox71* sites, and HAs (~98 bp). The correct transformants were screened on the LB agar plate containing 10 µg/mL gentamicin, and further verified by colony PCR using check primers (*KT-attB-out-1*, *KT-attB-out-2*, Supplementary Data 3) and DNA sequencing. Primers used in this section were listed in Supplementary Data 3.

### Cloning and engineering of BGCs

The BGCs of rhizomide, holrhizin, and rhizoxin from *P. rhizoxinica* HKI 454, the icosalide BGC from *B. gladioli* ATCC 10248, and epothilone BGC from *S. cellulosum* So ce90, were directly cloned and genetically engineered via Red/ET recombineering^10,34,46,62,72^, resulting in constructs of p15A-phiC31-apra-rhizomide, p15A-phiC31-apra-holrhizin, pBAC-cm-rhizoxin, p15A-TnpA-P_apra_-icosalide, and pBAC-cm-phiC31-apra-P11-epothilone, respectively. The plasmids pBAC-cm-phiC31-apra-rhizonxin and p15A-phiC31-amp-P_apra_-icosalide were constructed via recombineering. The chitinimide BGC (*chm*) from *C. koreensis* DSM 17726 and myxochelin BGC (*mxc*) from *M. xanthus* DK 1622 were directly cloned with primers (*chm-F*, *chm-R*, *mxc-F*, *mxc-R*) and inserted with *oriT-tnpA-IR* cassette and P_apra_ promoter, leading to constructs of p15A-TnpA-P_apra_-chm and p15A-TnpA-Tn5-km-mxc, respectively (Supplementary Data 3). The transposable element of *chm* and *mxc* BGCs were then replaced with the phiC31 site-specific integrase by recombineering, resulting in constructs of p15A-phiC31-amp-P_apra_-chm and p15A-phiC31-apra-Tn5-km-mxc, respectively^10,62,72^. In order to avoid the effects of replicable plasmids with p15A replicons on heterologous production yields of natural products in *E. coli* GB05-MtaA, the replicons of rhizomide, holrhizin, icosalide, myxochelin, and chitinimdie were replaced with pBAC vector, leading to the constructs: pBAC-cm-phiC31-apra-rhizomide, pBAC-cm-phiC31-apra-holrhizin, pBAC-spect-phiC31-amp-P_apra_-icosalide, pBAC-cm-phiC31-apra-Tn5-km-mxc, and pBAC-cm-phiC31-amp-P_apra_-chm, respectively, for heterologous expression in *E. coli* GB05-MtaA. The Cm antibiotic selection marker in pRK2-MMCoA-tRNA^34^ was replaced with Amp selection marker for screening, obtaining the construct of pRK2-amp-MMCoA-tRNA. Primers used in this section were listed in Supplementary Data 3. The rhizomide, holrhizin, rhizoxin, icosalide, epothilone, myxochelin, and chitinimide expression constructs were used for yield comparison by heterologous expression in *S. brevitalea* strains and two Gram-negative chassis strains *E. coli* GB05-MtaA and *P. putida* KT2440. The constructs harboring different BGCs were electroporated into *S. brevitalea* strains and *E.coli* GB05-MtaA. Each BGC was integrated into the specific landing point (phiC31 attB loci) of the chromosomes via site-specific integration. The correct transformants were then verified by colony PCR using check primers (Supplementary Data 3). For the heterologous expression of BGCs in *P. putida* KT2440, the constructs were introduced into the specific chromosomal site of *P. putida* with the help of donor strain *E. coli* WM3064, which is autotrophic for diaminopimelic acid (DAP)^85^. The obtained clones were verified by colony PCR and DNA sequencing using check primers (Supplementary Data 3). For the heterologous expression of epothilone, the correct transformants were screened on CYMG or LB agar plates containing apramycin, and then the plasmid pRK2-amp-MMCoA-tRNA was also electroporated into DSM 7029 strains and another two chassis harboring *epo* gene cluster in the chromosome and then verified with primers via colony PCR (*CoA-ins-check-F*, *CoA-ins-check-F*, Supplementary Data 3).

### Analysis of compounds from *S. brevitalea* mutants

The fermentation for BGCs in *S. brevitalea* strains were performed in normal CYMG liquid medium as described above. For the fermentation of the transformants containing epothilone gene cluster and methylmalonyl-CoA (MMCoA) -tRNA expression plasmid, the CYMG liquid medium was added with 100 mg/L methylmalonic acid to optimize the production yield^41^. Liquid seed cultures of *S. brevitalea* mutants carrying different gene clusters on the chromosome were inoculated from a plate in 2 mL CYMG liquid culture tubes. Seed cultures were incubated at 30 °C with 200 rpm shaking until achieving high cell density (typically 24 h). Seed cultures were diluted at the ratio of 1:50 into 50 mL of CYMG broth in 250 mL flasks, and the flash cultures were incubated at 30 °C, 200 rpm. After the cultures were incubated for 48 h, the resin Amberlite XAD-16 (2%) was added, and the mixture was incubated for another 48 h continually. The XAD-16 and all cells were harvested by centrifugation at 7647 × *g* for 5 min, and the mixture was extracted with 40 mL MeOH incubated at 30 °C with 200 rpm shaking for 2 h. Then the MeOH mixtures were evaporated to dryness after filtering by funnel. The crude extracts were resuspended in 1 mL MeOH for further HPLC-HRMS analysis. The HPLC system was performed using an ODS column (Luna RP-C18, 4.6 × 250 mm, 5 μm, 0.3 mL/min) with gradient elution (solvent A, H_2_O with 0.1% FA (formic acid); solvent B, 0.1% FA in acetonitrile (ACN); gradient at a constant flow rate of 0.3 mL/min, 0–3 min, 5% B; 3–19 min, 5–95% B; 19–22 min, 95% B; 23–25 min, 5% B, detection by UV spectroscopy at 200–600 nm). UV spectra was recorded on a DAD detector with wavelength ranging from 190 to 400 nm. The HRMS was measured on a Bruker Impact HD microTOF Q III mass spectrometer (BrukerDaltonics, Bremen, Germany) using the standard ESI source. Mass spectra was acquired in centroid mode ranging from 70 to 2200 m/z with positive-mode electrospray ionization and auto MS^2^ fragmentation. HPLC parameters were as follows: solvent A, H_2_O with 0.1% FA (formic acid); solvent B, 0.1% FA in acetonitrile (ACN); gradient at a constant flow rate of 0.3 mL/min, 0–3 min, 5% B; 3–19 min, 5–95% B; 19–22 min, 95% B; 23–25 min, 5% B, detection by UV spectroscopy at 200 to 600 nm.

The UHPLC-MS analysis of rhizomide, holrhizin, rhizoxin, icosalide, epothilone, and myxochelin were performed same as described before^34^. The relative yield was determined by comparison of the peak area in DSM 7029 WT and in genome-reduced strains, and peak area in WT was quantified as a reference (100%). The peak area of rhizomide A was calculated by extracted ion chromatogram (EIC) at 732.4 ± 0.5 (retention time at 10.4 min). The peak area of holrhizin A were calculated at 817.5 ± 0.5 (14.4 min). The peak area of icosalide A1 was calculated by EIC at 713.5 ± 0.5 (17.6 min). The peak areas of total rhizoxins were calculated by EICs as follows: 628.2 ± 0.5 (13.5 min), 628.2 ± 0.5 (14 min), 642.3 ± 0.5 (15.5 min), 610.2 ± 0.5 (15.5 min), and 610.2 ± 0.5 (16.1 min). The peak area of epothilone C and D were calculated by EICs at 478.3 ± 0.5 and 492.3 ± 0.5. The peak areas of chitinimides A and B were calculated by EICs at 944.4 ± 0.5 (10.7 min) and 1114.5 ± 0.5 (10.5 min). The peak areas of myxochelins A and B were calculated by EICs at 405.2 ± 0.5 (9.8 min) and 404.2 ± 0.5 (8.0 min).

### Purification of chitinimides

The 2% resin XAD-16 was added into the fermentation broth (20 L) after 2 days cultivation and cultivated at 30 °C, 200 rpm for further 2 days. The resin XAD-16 was collected and repeatedly washed with ddH_2_O, and then extracted with MeOH (15 L). The resulting crude extract was firstly fractionated on a normal-phase silica gel column via different ratios of MeOH and dichloromethane as mobile phases to perform gradient solution, leading to 60 fractions totally. For target compounds **3** and **4**, fractions (Fr. 36-Fr. 43) were concentrated and subjected to a Sephadex LH-20 column using MeOH as mobile phase. Then, the fractions containing target compounds **3** and **4** were subjected to a semipreparative reverse-HPLC system (Agilent ZORBAXSB-C18, 9.4 × 250 mm, 5 μm, DAD at 210 nm). The reverse-HPLC parameters were as follows: solvent A, H_2_O with 0.3% TFA (trifluoroacetic acid), solvent B, MeOH; gradient at a flow rate of 3 mL/min, 0–5 min 48% B, 5–35 min 48% B, 35–45 min 100% B, 45–50 min 48% B to acquire chitinimide C (**3**) and D (**4**) mixture (15 mg) with retention time at 41.5 min. For compounds **1** and **2**, fractions (Fr. 44-Fr. 60) were concentrated and subjected to a Sephadex LH-20 column using MeOH and ddH_2_O with 0.1% formic acid addition as mobile phases. Then, the fractions containing target compounds **1** and **2** were subjected to a semipreparative reverse-HPLC system (Agilent ZORBAXSB-C18, 9.4 × 250 mm, 5 μm, DAD at 210 nm). The reverse-HPLC parameters were set as follows: solvent A, H_2_O with 0.2% TFA, solvent B, MeOH; gradient at a flow rate of 3 mL/min, 0–5 min 32% B, 5–25 min 32% B, 25–35 min 100% B, 35–45 min 32% B to acquire chitinimide A (**1**) with 30 mg and B (**2**) with 7.6 mg, and the retention time were at 21 min and 23 min, respectively. *Chitinimide A (****1****)*: colorless oil; [*α*]^20^_D_-22 (*c* 0.16, MeOH); UV (MeOH) *λ*_max_ 215 nm; IR (KBr) *v*_max_ 3344, 2929, 2932, 1673, 1550, 1448, 1208, 1143, 843, 801, 724 cm^−1^; ^1^H and ^13^C NMR, Table S5; HRESIMS *m/z* 944.3776 [M + H]^+^ (*calcd* for C_41_H_58_N_11_O_11_S_2_ 944.3753). *Chitinimide B (****2****)*: colorless oil; [*α*]^20^_D_-12 (*c* 0.1, MeOH); UV (MeOH) *λ*_max_ 215 nm; IR (KBr) *v*_max_ 3362, 2927, 1675, 1458, 1209, 1147, 847, 801, 725 cm^−1^; ^1^H and ^13^C NMR, Table S5; HRESIMS *m/z* 1114.4467 [M + H]^+^ (*calcd* for C_48_H_68_N_13_O_14_S_2_ 1114.4445). *Chitinimide C (****3****) and Chitinimide D (****4****)*: mixture; UV (MeOH) *λ*_max_ 215 nm; ^1^H and ^13^C NMR, Table S6; HRESIMS *m/z* 1184.5240 [M + H]^+^ (*calcd* for C_53_H_78_N_13_O_14_S_2_ 1184.5227) and 1242.5286 [M + H]^+^ (*calcd* for C_55_H_80_N_13_O_16_S_2_ 1242.5284).

### Marfey’s analysis of the amino acid constituents of chitinimides

Marfey’s analysis was performed as reported previously^73,74^. In brief, chitinimide A (1 mg) was dissolved in 100 μL of 6 M HCl. The reaction was stood at 60 °C overnight for drying, then the acid hydrolysates of chitinimide A were redissolved in ddH_2_O (200 μL), and 100 μL Marfey’s reagent in acetone was added, followed by 1 M NaHCO_3_ (25 μL). The mixtures were heated for 1 h at 40 °C and 100 μL 2 M HCl were added to quench the reaction. Finally, the resulting solution was filtered through a small 2.5 μm filter and analyzed by LC–MS with a linear gradient of ACN and 0.1% formic acid aqueous solution with different elution conditions at a flow rate of 0.3 mL/min and UV detection at 330 nm. Amino acid standards were derivatized with Marfey’s reagent in a similar manner. For the analysis of L/D-Ser, L/D-Met, L/D-Arg, and L/D-Phe, the elution condition is a linear gradient of ACN 5–95% in 16 min. As for the analysis of L/D-Ile and allo-L/D/Ile, the elution condition is a linear gradient of ACN 5–50% in 36 min. Each chromatographic peak was identified by comparing their retention times and molecular weight of the Marfey’s reagent derivatives to the L- and D-amino acid standards independently^74^. The retention times and configurations of amino acids in chitinimide A (**1**) and chitinimides C/D (**3/4**) were listed in Supplementary Table 6.

### Biological activity screening of chitinimides

Antagonism of blasticidin S in *Bacillus cereus* was consistent with previous description and the procedure was also performed as reported^70^. Briefly, paper disks were treated with 10 µL of 1 mg/mL blasticidin S, 10 µL of 10 mg/mL chitinimides, or 10 µL of mixed stocks. Disks were placed on a LB plate seeded with 30 µL of pre-grown *B. cereus* incubated at 37 °C for 12 h (Supplementary Fig. 7). Three human cancer cell lines (human acute lymphoblastic leukemia cells Kasumi, human breast cancer cells MDA-MB-231, human lung carcinoma cell line A549) and one human normal lung epithelial cells line BEAS-2B were utilized to test the cytotoxicity of compounds **1**–**4** by MTT assay^88^ (Supplementary Table 7). The cathepsin B/L assays were performed in triplicate according to a modified method as published^89^.

### Reporting summary

Further information on research design is available in the Nature Research Reporting Summary linked to this article.

## Supplementary information

Supplementray information

Supplementary Data 1

Supplementary Data 2

Supplementary Data 3

Description of additional supplementary files

Reporting Summary

## Data Availability

Data supporting the findings of this work are available within the paper and its Supplementary Information files. A reporting summary for this Article is available as a Supplementary Information file. The chitinimide BGC has been deposited in Genbank under accession number MW160162. Source data are provided with this paper.

## Source data

Source data
