# Supplementary material for: Rational construction of genome-reduced Burkholderiales chassis facilitates efficient heterologous production of natural products from proteobacteria
Source: Nat Commun. 2021 Jul 23;12:4347. doi: 10.1038/s41467-021-24645-0 (PMC8302735; doi:10.1038/s41467-021-24645-0)
Supplement: Supplementary file 1 — Supplementray information [file 41467_2021_24645_MOESM1_ESM.pdf]

# **Rational construction of genome-reduced Burkholderiales chassis facilitates efficient heterologous production of natural products from proteobacteria**

Liu *et al.*

**Supplementary Table 1. Pros and cons of DSM 7029 and other widely-used bacterial chassis for heterologous expression of natural products.**

| Chassis cell | characteristics | | Construction strategies | Ref. |
|---|---|---|---|---|
| | Pros | cons | | |
| *Escherichia coli* | Abundant precursors, such as acetyl-CoA, malonyl-CoA, mevalonate, and shikimate, *et al*. | Lacks post-translational modification system and intracellular membranes | Pathway engineering, genetic modification to facilitate the accumulation of multiple metabolic precursors. | [1] |
| *Bacillus subtilis* | Used to produce vitamins and functional sugars | Lacks stable gene expression system | Genome minimization, synthetic biology toolboxes and genome editing tools development. | [2, 3] |
| *Streptomyces* spp. | Ability to produce multiple metabolites, such as PKs, NRPS and terpenes. | Much longer fermentation and genetic manipulation cycles, difficult to modify most industrial *streptomyces* | Rational genome reduction to simplify the metabolic background, reprogramming biosynthetic pathways, transcriptional regulator modification, metabolic and morphology engineering, and advanced synthetic biology tools development | [4] |
| *Pseudomonas* spp. | Grow fast, abundant PPTases, high xenobiotic tolerance, production of phenazine and its derivatives | lacks abundant precursors for secondary metabolites | Development of diverse genetic manipulation tools and inducible promoter systems, genome reduction to simplify metabolic background | [5] |
| *Schlegelella brevitalea* DSM 7029 | Used to produce multiple secondary metabolites from myxobacteria and Burholderiales strains, easy to operate genetic manipulation | Relative complicated metabolic background, slow growth | Establishment of genome editing tools, strong promoter screening and synthetic biology tools development, metabolic pathway engineering | [6, 7] |
| *Myxococcus xanthus* | Model strain of myxobacteria, a relative clear lifecycle, has the potential to produce various bioactive secondary metabolites | Slow growth rate, lacks efficient and advanced genetic manipulation tools | Modification of heterologous production platform by promoter changing, combinatorial engineering of complex synthetic BGCs, elimination of endogenous metabolic background | [8, 9] |

**Supplementary Table 2. Minimum inhibitory concentration (MIC) assay.**

| DSM 7029 strains | **Minimum Inhibitory Concentration (MIC) of selected antibiotics (μg/mL)** | | | | | | |
|---|---|---|---|---|---|---|---|
| | Amp | Hyg | Cm | Tet | Km | Genta | Apra |
| WT | ＞32 | ＞1024 | ＞8 | ＞32 | ＞32 | ＞32 | ＞64 |
| DT6 | ＞8 | ＞512 | ＞32 | ＞64 | ＞64 | ＞32 | ＞32 |
| DT7 | ＞16 | ＞512 | ＞16 | ＞64 | ＞32 | ＞16 | ＞16 |
| DT8 | ＞16 | ＞512 | ＞32 | ＞64 | ＞32 | ＞16 | ＞32 |
| DT9 | ＞16 | ＞512 | ＞32 | ＞64 | ＞16 | ＞16 | ＞16 |
| DT10 | ＞4 | ＞512 | ＞16 | ＞64 | ＞32 | ＞4 | ＞16 |

**Supplementary Table 3. Annotation of the chitinimide biosynthetic gene cluster.**

| ORF | No. of amino acids | Function in Chitinimide BGC | Homolog function | Homolog accession | Fractional amino acid identity (%) |
|---|---|---|---|---|---|
| *chmL* | 83 | | MbtH family NRPS accessory protein | WP_008447535.1 | 42/72 (58) |
| *chmA* | 2558 | $A_{Phe}$-PCP-$C_2$-$A_{Dhb1}$-PCP-$C_3$-$A_{Ser1}$-PCP | Non-ribosomal peptide synthetase | WP_090208411.1 | 1623/2781 (58) |
| *chmB* | 4200 | $C_4$-$A_{Dhb2}$-PCP-$C_5$-$A_{Arg}$-PCP-$C_6$-$A_{Pro}$-PCP-$C_7$-$A_{Cys}$-PCP | Non-ribosomal peptide synthetase | RLK09907.1 | 2927/4444 (66) |
| *chmC* | 1684 | $C_8$-$A_{Met}$-PCP-TE | Non-ribosomal peptide synthetase | WP_052853215.1 | 1002/1384 (72) |
| *chmD* | 3083 | $A_{Ile}$-PCP-$C_9$-$A_{Pro}$-PCP-KS-KR-ACP-TE | Non-ribosomal peptide synthetase | WP_101403278.1 | 2123/3122 (68) |
| *chmE* | 298 | | Sterol desaturase family protein | WP_00802760.1 | 92/179 (51) |
| *chmF* | 242 | | 4’-phosphopantetheinyl transferase superfamily protein | WP_052853123.1 | 164/234 (77) |
| *chmG* | 504 | Thioesterase | Thioesterase/alpha/beta fold hydrolase | WP_052853213.1 | 164/234 (77) |
| *chmH* | 352 | | GSCFA domain-containing protein | WP_144322328.1 | 244/349 (70) |
| *chmI* | 230 | | Hypothetical protein | Hypothetical protein | 99/180 (55) |
| *chmJ* | 292 | | Taurine dioxygenase | Taurine dioxygenase | 237/291 (81) |
| *chmK* | 202 | | Hypothetical protein | Hypothetical protein | 157/225 (70) |

**Supplementary Table 4. The $^{1}H$ (500 MHz) and $^{13}C$ NMR (125 MHz) Data of 1 and 2 in DMSO-$d_6$.**

| no. | **1** $\delta_C$ | **1** $\delta_H$ ($J$ in Hz) | **2** $\delta_C$ | **2** $\delta_H$ ($J$ in Hz) |
|---|---|---|---|---|
| 1 | 172.1, C | | 172.1, C | |
| 2 | 51.1, CH | 4.63, m | 51.1, CH | 4.62, m |
| 3 | 30.0, $CH_2$ | 2.13, m[a] | 30.0, $CH_2$ | 2.12, m[a] |
| 4 | 29.9, $CH_2$ | 2.52, m | 29.9, $CH_2$ | 2.53, m |
| 5 | 14.6, $CH_3$ | 2.04, s | 14.6, $CH_3$ | 2.04, s |
| 6 | 160.7, C | | 160.7, C | |
| 7 | 149.0, C | | 149.0, C | |
| 8 | 124.2, CH | 8.16, s | 124.2, CH | 8.18, s |
| 9 | 174.0, C | | 174.0, C | |
| 10 | 58.4, CH | 5.23, dd (1.8, 8.0) | 58.5, CH | 5.32, dd (1.8, 8.1) |
| 11 | 31.5, $CH_2$ | 2.17, m[a] | 31.5, $CH_2$ | 2.26, m |
| | | 2.12, m[a] | | 2.17, m[a] |
| 12 | 24.0, $CH_2$ | 1.91, m[a] | 24.0, $CH_2$ | 1.98, m[a] |
| 13 | 46.8, $CH_2$ | 3.78, m[a] | 46.8, $CH_2$ | 3.78, m[a] |
| | | 3.71, m[a] | | 3.71, m[a] |
| 14 | 170.7, C | | 170.7, C | |
| 15 | 50.4, CH | 4.55, m | 50.5, CH | 4.59, m |
| 16 | 28.2, $CH_2$ | 1.74, m[a] | 28.1, $CH_2$ | 1.75, m[a] |
| | | 1.56, m[a] | | 1.55, m[a] |
| 17 | 24.8, $CH_2$ | 1.52, m | 24.8, $CH_2$ | 1.52, m |
| 18 | 40.5, $CH_2$ | 3.07, m | 40.5, $CH_2$ | 3.07, m |
| 19 | 156.8, C | | 156.7, C | |
| 20 | 163.5, C | | 163.5, C | |
| 21 | 130.0, C | | 129.9, C | |
| 22 | 130.5, CH | 6.52, q (7.0) | 130.5, CH | 6.52, q (6.9) |
| 23 | 13.0, $CH_3$ | 1.61, d (7.0) | 13.0, $CH_3$ | 1.62, d (6.9) |
| 24 | 170.1, C | | 169.8, C | |
| 25 | 56.4, CH | 4.19, m | 56.5, CH | 4.18, m |
| 26 | 61.5, $CH_2$ | 3.78, m[a] | 61.4, $CH_2$ | 3.78, m[a] |
| | | 3.71, m[a] | | 3.71, m[a] |
| 27 | 166.1, C | | 166.3, C | |
| 28 | 132.2, C | | 129.8, C | |
| 29 | 120.5, CH | 5.90, q (6.8) | 129.5, CH | 6.47, q (6.7) |
| 30 | 12.2, $CH_3$ | 1.63, d (6.8) | 13.0, $CH_3$ | 1.64, d (6.7) |
| 31 | 155.6, C | | 170.5, C | 4.23, m |
| 32 | 173.1, C | | 56.8, CH | 3.78, m[a] |
| 33 | 54.0, CH | 4.32, dd (6.3, 7.7) | 61.0, $CH_2$ | 3.71, m[a] |
| 34 | 37.9, $CH_2$ | 2.94, d (6.3) | 166.6, C | |
| 35 | 137.1, C | | 131.8, C | 5.94, q (6.2) |
| 36 | 129.4, CH | 7.14, d (7.6) | 121.3, CH | 1.64, d (6.2) |

| | | | | |
|---|---|---|---|---|
| 37 | 128.2, CH | 7.26, t (7.6) | 12.3, $CH_3$ | |
| 38 | 126.5, CH | 7.20, d (7.6) | 155.5, C | |
| 39 | 128.2, CH | 7.26, t (7.6) | 173.1, C | |
| 40 | 129.4, CH | 7.14, d (7.6) | 53.9, CH | 4.35, m |
| 41 | | | 37.6, $CH_2$ | 2.99, dd (5.5, 14.0) |
| 42 | | | 137.10, C | |
| 43/47 | | | 129.4, CH | 7.18, d (7.5) |
| 44/46 | | | 128.2, CH | 7.28, t (7.5) |
| 45 | | | 126.5, CH | 7.22, d (7.5) |
| 1′ | 52.1, $CH_3$ | 3.65, s | 52.1, $CH_3$ | 3.65, s |
| 2-NH | | 8.63, d (8.0) | | 8.64, d (8.0) |
| 15-NH | | 7.52, d (7.6) | | 7.56, d (7.7) |
| 18-NH | | 7.65, brs | | 7.54, brs |
| 21-NH | | 9.16, s | | 8.95, s |
| 25-NH | | 7.87, d (6.5) | | 7.61, d (5.6) |
| 28-NH | | 8.10, s | | 9.31, s |
| 32-NH | | | | 7.97, d (5.9) |
| 33-NH | | 6.72, d (7.7) | | |
| 35-NH | | | | 8.18, s |
| 40-NH | | | | 6.64, d (7.7) |

[a] overlapped

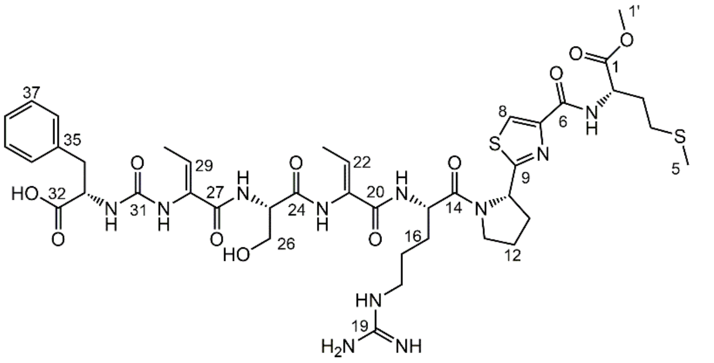

1

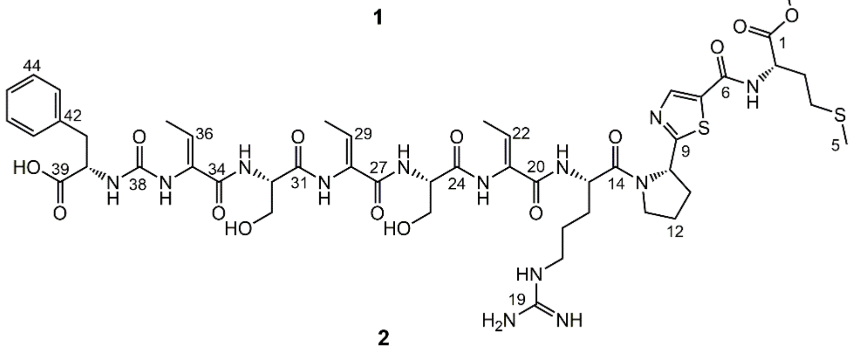

2

**Supplementary Table 5. The $^{1}$H (500 MHz) and $^{13}$C NMR (125 MHz) Data of 3 and 4 in DMSO-$d_6$.**

| no. | **3** | | **4** | |
|---|---|---|---|---|
| | $\delta_C$ | $\delta_H$ ($J$ in Hz) | $\delta_C$ | $\delta_H$ ($J$ in Hz) |
| 1 | 170.5, C | | 170.1, C | |
| 2 | 51.4, CH | 4.58, m | 51.2, CH | 4.61, m |
| 3 | 29.6, $CH_2$ | 2.10, m[a] | 29.6, $CH_2$ | 2.10, m[a] |
| 4 | 29.9, $CH_2$ | 2.53, m | 29.9, $CH_2$ | 2.53, m |
| 5 | 14.6, $CH_3$ | 2.04, s | 14.6, $CH_3$ | 2.04, s |
| 6 | 160.7, C | | 160.6, C | |
| 7 | 149.0, C | | 149.0, C | |
| 8 | 124.2, CH | 8.17, s | 124.2, CH | 8.17, s |
| 9 | 174.1, C | | 174.1, C | |
| 10 | 58.4, CH | 5.24, dd (1.6, 7.8) | 58.4, CH | 5.24, dd (1.6, 7.8) |
| 11 | 31.6, $CH_2$ | 2.20, m[a] | 31.6, $CH_2$ | 2.20, m[a] |
| | | 2.10, m[a] | | 2.10, m[a] |
| 12 | 23.9, $CH_2$ | 1.91, m[a] | 23.9, $CH_2$ | 1.91, m[a] |
| 13 | 46.7, $CH_2$ | 3.78, m[a] | 46.7, $CH_2$ | 3.78, m[a] |
| | | 3.71, m[a] | | 3.71, m[a] |
| 14 | 170.8, C | | 170.8, C | |
| 15 | 50.5, CH | 4.54, m | 50.5, CH | 4.54, m |
| 16 | 28.1, $CH_2$ | 1.75, m[a] | 28.1, $CH_2$ | 1.75, m[a] |
| | | 1.57, m[a] | | 1.57, m[a] |
| 17 | 24.8, $CH_2$ | 1.52, m[a] | 24.8, $CH_2$ | 1.52, m[a] |
| 18 | 40.4, $CH_2$ | 3.08, m | 40.4, $CH_2$ | 3.08, m |
| 19 | 156.9, C | | 156.9, C | |
| 20 | 163.5, C | | 163.5, C | |
| 21 | 130.0, C | | 130.0, C | |
| 22 | 130.4, CH | 6.52, q (7.0) | 130.4, CH | 6.52, q (7.0) |
| 23 | 13.0, $CH_3$ | 1.60, d (7.0) | 13.0, $CH_3$ | 1.60, d (7.0) |
| 24 | 170.1, C | | 170.1, C | |
| 25 | 56.4, CH | 4.19, dd (4.4, 10.4) | 56.4, CH | 4.19, dd (4.4, 10.4) |
| 26 | 61.4, $CH_2$ | 3.78, m[a] | 61.4, $CH_2$ | 3.78, m[a] |
| | | 3.71, m[a] | | 3.71, m[a] |
| 27 | 166.1, C | | 166.1, C | |
| 28 | 132.1, C | | 132.1, C | |
| 29 | 120.6, CH | 5.90, q (7.0) | 120.6, CH | 5.90, q (7.0) |
| 30 | 12.2, $CH_3$ | 1.63, d (7.0) | 12.2, $CH_3$ | 1.63, d (7.0) |
| 31 | 155.6, C | | 155.6, C | |
| 32 | 173.1, C | | 173.1, C | |
| 33 | 54.0, CH | 4.32, dd (5.5, 6.2) | 54.0, CH | 4.32, dd (5.5, 6.2) |
| 34 | 37.9, $CH_2$ | 2.94, d (5.5) | 37.9, $CH_2$ | 2.94, d (5.5) |
| 35 | 137.1, C | | 137.1, C | |
| 36/40 | 129.4, CH | 7.14, d (7.1) | 129.4, CH | 7.14, d (7.1) |

| | | | | |
|---|---|---|---|---|
| 37/39 | 128.2, CH | 7.26, t (7.1) | 128.2, CH | 7.26, t (7.1) |
| 38 | 126.5, CH | 7.20, d (7.1) | 126.5, CH | 7.20, d (7.1) |
| 1′ | 171.5, C | | 171.1, C | |
| 2′ | 35.2, $CH_2$ | 2.43, m[a] | 35.7, $CH_2$ | 2.47, m[a] |
| 3′ | 72.5, CH | 5.46, m | 69.4, CH | 5.37, m |
| 4′ | 57.4, CH | 4.46, m | 57.3, CH | 4.76, dd (1.6, 7.5) |
| 5′ | 24.2, $CH_2$ | 1.91, m[a] | 70.7, CH | 5.10, dd (7.5, 15.0) |
| | | 1.52, m[a] | | |
| 6′ | 25.8, $CH_2$ | 1.91, m[a] | 29.8, $CH_2$ | 2.20, m[a] |
| | | 1.75, m[a] | | 2.00, m[a] |
| 7′ | 47.9, $CH_2$ | 3.71, m[a] | 44.9, $CH_2$ | 3.78, m[a] |
| | | 3.33, m[a] | | 3.46, m[a] |
| 8′ | 168.4, C | | 169.4, C | |
| 9′ | 55.4, CH | 4.08, d (4.5) | 54.6, CH | 4.09, d (3.4) |
| 10′ | 35.9, CH | 1.75, m[a] | 36.0, CH | 1.75, m[a] |
| 11′ | 23.5, $CH_2$ | 1.41, m | 23.6, $CH_2$ | 1.41, m |
| | | 1.07, m | | 1.07, m |
| 12′ | 11.3, $CH_3$ | 0.80, t (7.3) | 11.2, $CH_3$ | 0.77, t (7.4) |
| 13′ | 14.9, $CH_3$ | 0.92, d (7.0) | 14.7, $CH_3$ | 0.86, d (6.8) |
| 14′ | | | 169.7, C | |
| 15′ | | | 20.6, $CH_3$ | 2.00, s |
| 2-NH | | 8.62, d (8.2) | | 8.73, d (8.3) |
| 15-NH | | 7.51, d (7.6) | | 7.51, d (7.6) |
| 18-NH | | 7.76, brs | | 7.76, brs |
| 21-NH | | 9.16, s | | 9.16, s |
| 25-NH | | 7.87, d (6.5) | | 7.87, d (6.5) |
| 28-NH | | 8.18, s | | 8.18, s |
| 33-NH | | 6.75, d (6.2) | | 6.76, d (6.2) |
| 9′-$NH_2$ | | 8.10, brs | | 8.16, brs |

[a] overlapped

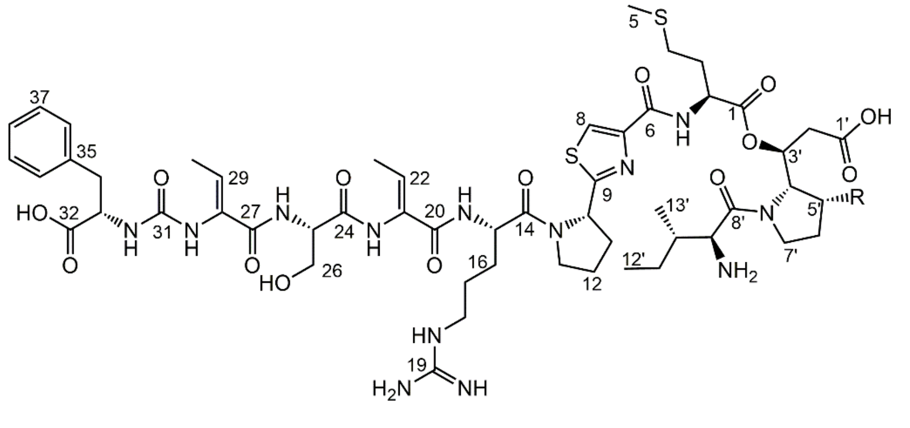


**3** R=H
**4** R=OAc

**Supplementary Table 6. Retention times of amino acids derivatized with Marfey's reagent (L-FDAA).**

| Amino acid | Configuration | Retention times of hydrolyzed compounds | | |
|---|---|---|---|---|
| | | Standard amino acid | **1** | **3/4** |
| Phe | L | 12.3 | 12.4 | |
| | D | 12.9 | | |
| Ser | L | 9.3 | 9.3 | |
| | D | 9.4 | | |
| Met | L | 11.4 | 11.4 | |
| | D | 12.1 | | |
| Arg | L | 14.1 | 14.1 | |
| | D | 13.8 | | |
| Ile | L | 29.5 | | 29.5 |
| | D | 33.0 | | |
| | L-*allo* | 29.6 | | |
| | D-*allo* | 32.9 | | |

**Supplementary Table 7. The cytotoxicity of chitinimide A (1) and B (2).**

| Compounds | ($IC_{50}$/μM) | | | |
|---|---|---|---|---|
| | Human cancer cell lines | | | Human normal cell lines |
| | Kasumi | MDA-MB-231 | A549 | BEAS-2B |
| chitinimide A (**1**) | ＞20 | ＞20 | ＞20 | ＞20 |
| chitinimide B (**2**) | 21.39±2 | 14.4±3.13 | ＞20 | ＞40 |
| Dox[a] | 0.097±0.01 | 0.63±0.07 | 0.36±0.12 | 0.53±0.45 |

[a] Doxorubicin as positive control.

**Supplementary Table 8. The inhibitory activity of chitinimide A-D (1-4) on Cathepsin B and Cathepsin L.**

| Compounds | $IC_{50}$ (μg/mL) | |
|---|---|---|
| | cathepsin B | cathepsin L |
| chitinimide A (**1**) | 6.01 | >25 |
| chitinimide B (**2**) | 12.5 | >25 |
| chitinimide C and D (**3** and **4**) | 12.5 | >25 |

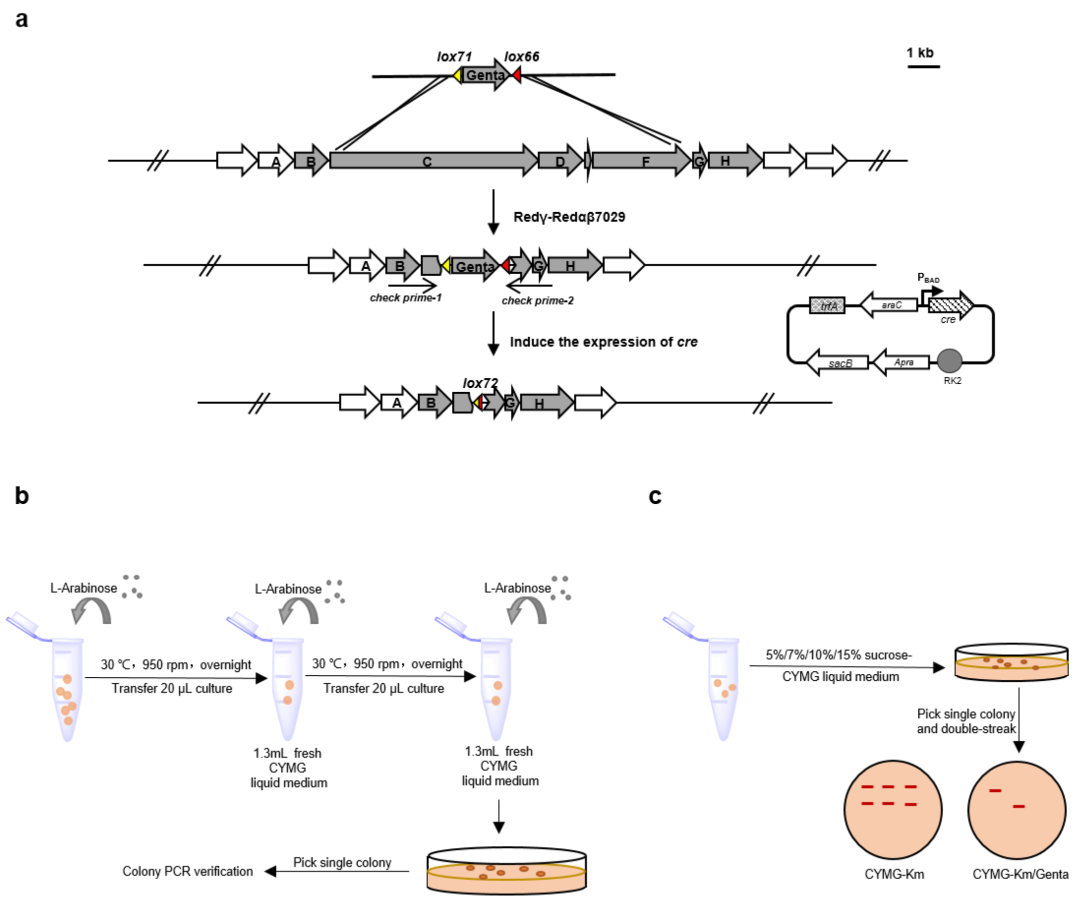


**Supplementary Fig. 1. Scheme diagram of the established genome reduction method in DSM 7029. a**. The markerless deletion of glidobactin BGC (*glb*, BGC5), the Genta resistance selection marker flanked with *lox66*, *lox71* sites and corresponding homology arms (HAs) were used to replace *glbC-glbF* via Redγ-Redαβ7029 recombinase, and the correct recombinant was verified by colony PCR. Then the Cre expression plasmid was transferred to perform Cre/loxP site-specific recombination for removal of resistance marker. **b**. The Genta selection marker on the genome was eliminated by induction of Cre site-specific recombinase by adding L-(+)-Arabinose for several times, and the correct single clone was verified by colony PCR. **c**. The Cre expression plasmid was eliminated in the markerless deletion mutant by culturing in CYMG liquid medium added with different concentration gradient of sucrose via SacB counterselection, and the correct single clone screened on CYMG plate containing kanamycin was further verified by colony PCR.

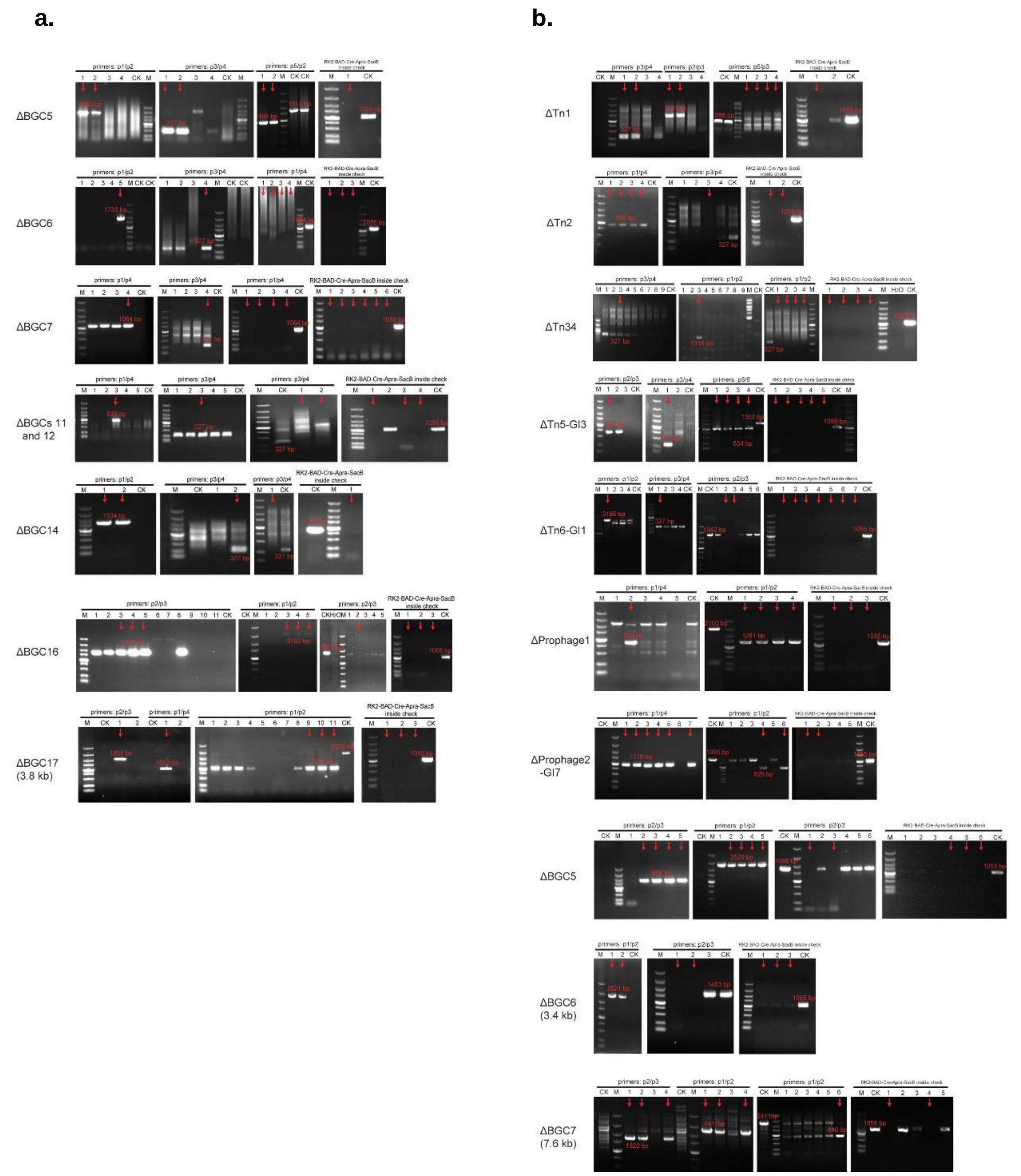


**Supplementary Fig. 2. Colony PCR verification of the deleted genomic regions and elimination of Cre site-specific recombinase expression plasmid in the genome-reduced mutants of DC series (a) and DT series (b).** M represents DL5000 DNA Marker and the molecular weight are displayed as 100 bp, 250 bp, 500 bp, 750 bp, 1000 bp, 1500 bp, 2000 bp, 3000 bp, and 5000 bp from bottom to top. CK represents the negative blank control. The panels marked with red arrows represent correct colonies, and the corresponding molecular weight were labelled beside the bands. The experiment was repeated twice independently with similar results.

**a**

| Single deletion of hypothetical QS-related and lytic genes | | | |
|---|---|---|---|
| Tn1 | 1 | AAW51_RS01950 | AraC family transcriptional regulator |
| Tn2 | 2 | AAW51_RS08255 | MerR family transcriptional regulator |
| | 3 | AAW51_RS08295 | MarR family transcriptional regulator |
| | 4 | AAW51_RS08365 | TetR/AcrR family transcriptional regulator |
| Tn34 | 5 | AAW51_RS20290 | AraCfamily transcriptional regulator |
| | 6 | AAW51_RS20310 | TetR/AcrR family transcriptional regulator |
| | 7 | AAW51_RS20335 | YafY family transcriptional regulator |
| | 8 | AAW51_RS20380 | AraC family transcriptional regulator |
| Tn34 | 9 | AAW51_RS20450 | TetR/AcrR family transcriptional regulator |
| Tn6-GI1 | 10 | AAW51_RS26360 | XRE family transcriptional regulator |
| | 11 | AAW51_RS26400 | LysR family transcriptional regulator |
| Pro2-GI7 | 12 | AAW51_RS19695 | hypothetical protein |
| | 13 | AAW51_RS19715 | LysR family transcriptional regulator |
| | 14 | AAW51_RS19800 | RNA polymerase subunit sigma |
| | 15 | AAW51_RS19965<br>AAW51_RS19970<br>AAW51_RS29400 | membrane protein<br>membrane protein<br>HNH endonuclease |
| | 16 | AAW51_RS19980 | Lysozyme |

**b**

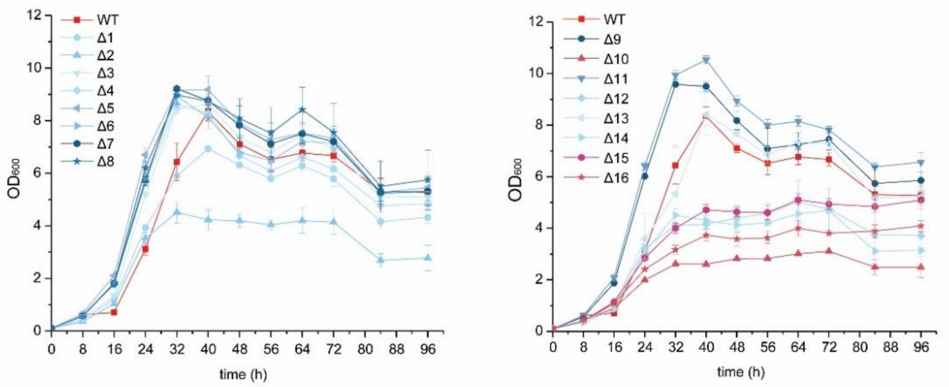


**c**

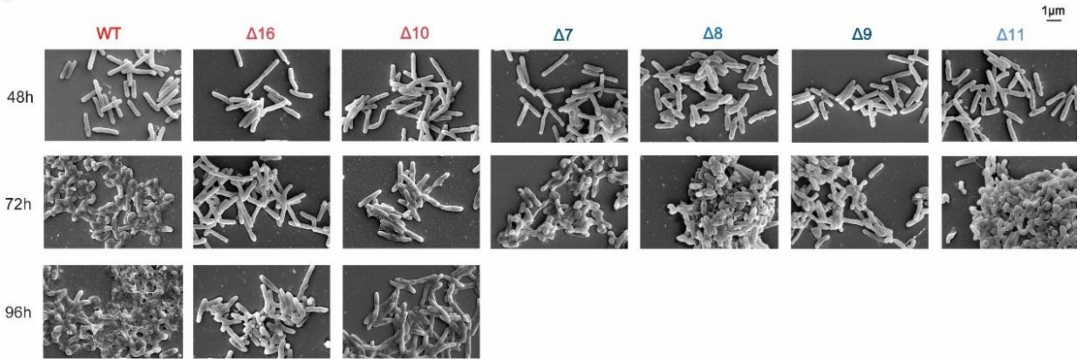


**Supplementary Fig. 3. Single gene inactivation of hypothetical quorum sensing (QS) system-related and lytic genes. a.** Sixteen hypothetical QS-related and lytic genes (1-16) within the transposases, GIs, and prophages regions deleted in the genome-reduced DSM 7029 strains of DT series. **b.** Systematic growth profiles of single gene inactivation mutants of DSM 7029 containing Redγ-Redαβ7029 recombinase expression plasmid (DSM 7029-Redγ-Redαβ7029) were measured by culturing in normal CYMG medium after unforming the starting $OD_{600}$ values. The growth curves of Δ1 to Δ8 mutants as well as the DSM 7029-Redγ-Redαβ7029 were illustrated in the left. The growth curves of Δ9 to Δ16 mutants as well as DSM 7029-Redγ-Redαβ7029 were illustrated in the right. Data are presented as mean values +/- SD. n=3 biologically independent samples. **c.** Cell morphology determination of DSM 7029-Redγ-Redαβ7029 and six single gene inactivation mutants (Δ16, Δ10, Δ7, Δ8, Δ9, and Δ11) by using the field emission scanning electron microscopy (FESEM) at 48 h, 72 h, and 96 h, respectively. The experiment was repeated twice independently with similar results. Source data underlying Supplementary Figure 3b are provided as a Source Data file.

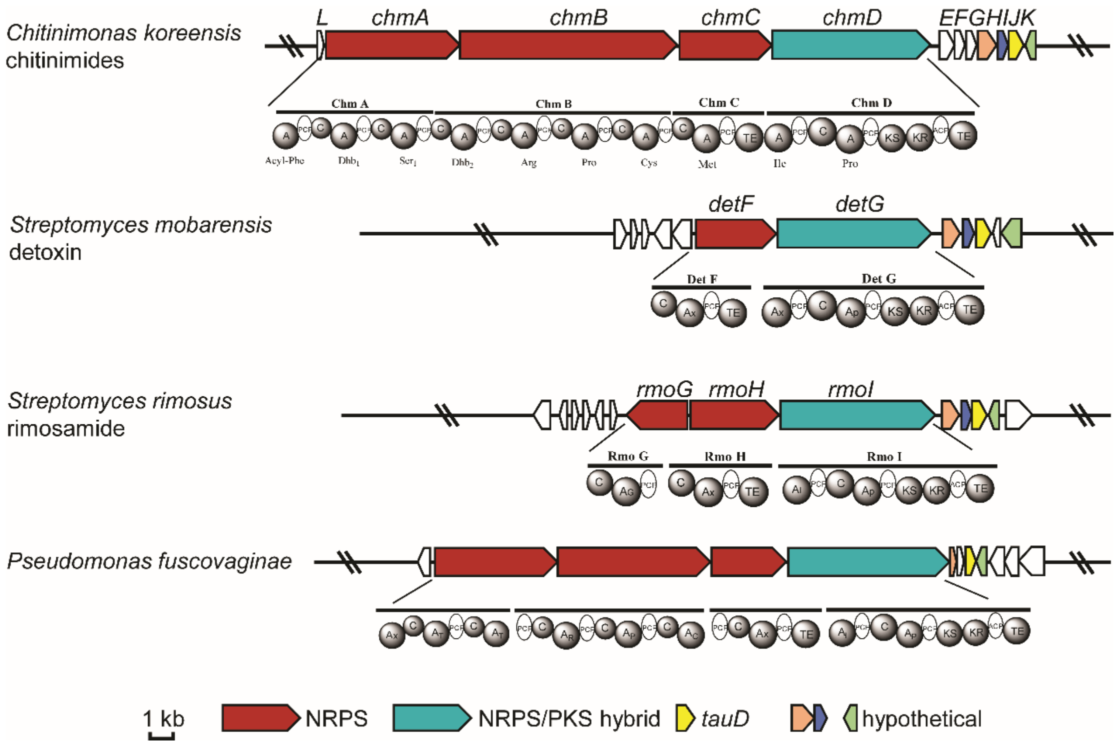


**Supplementary Fig. 4. Comparative analysis of chitinimide BGC (*chm*).** The chitinimide (top) shares several ORFs that of high identity with the detoxin and rimosamide BGCs in *Streptomyces* spp., as well as undefined BGC in *Pseudomonas* sp.. Homologous ORFs were marked with identical color and the encoding domains were listed below the corresponding cluster.

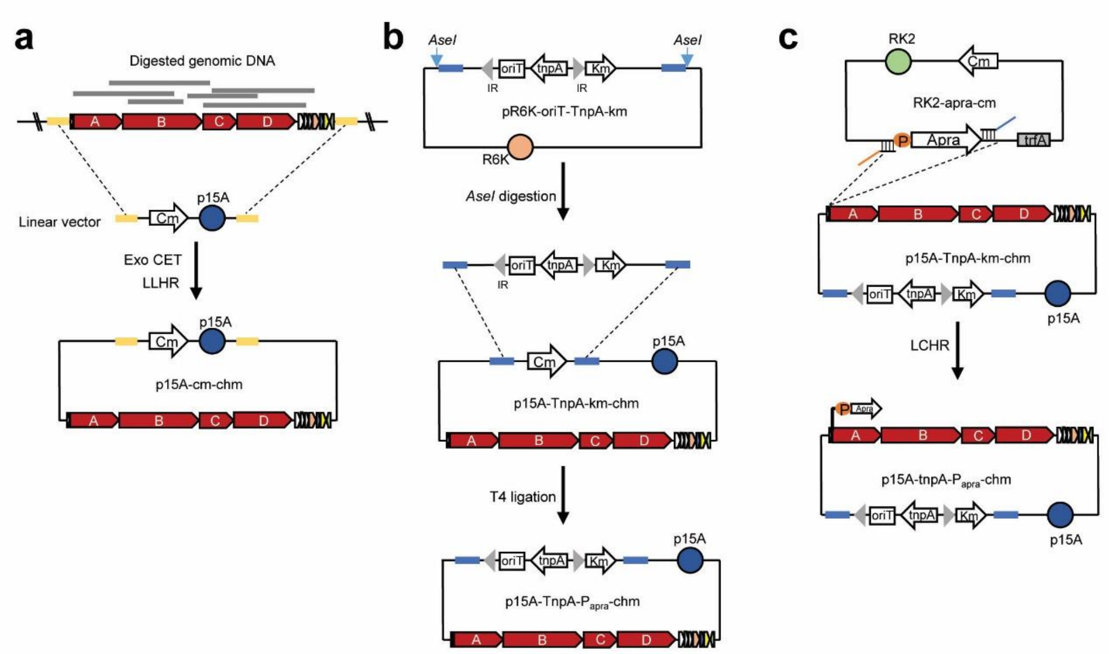


**Supplementary Fig. 5. Procedures of direct cloning and engineering of chitinimide BGC (*chm*)**. Homology arms were marked with bright color. **a**. The direct cloning of chitinimide biosynthetic gene cluster, resulting the construct of p15A-cm-chm. **b**. The insertion of *oriT-tnpA-IR* transposable element, resulting the construct of p15A-TnpA-km-chm. **c**. The insertion of $P_{apra}$ constitutive promoter, resulting the construct of p15A-TnpA-$P_{apra}$-chm. The direct cloning and engineering were performed in *E. coli* via Red/ET recombineering

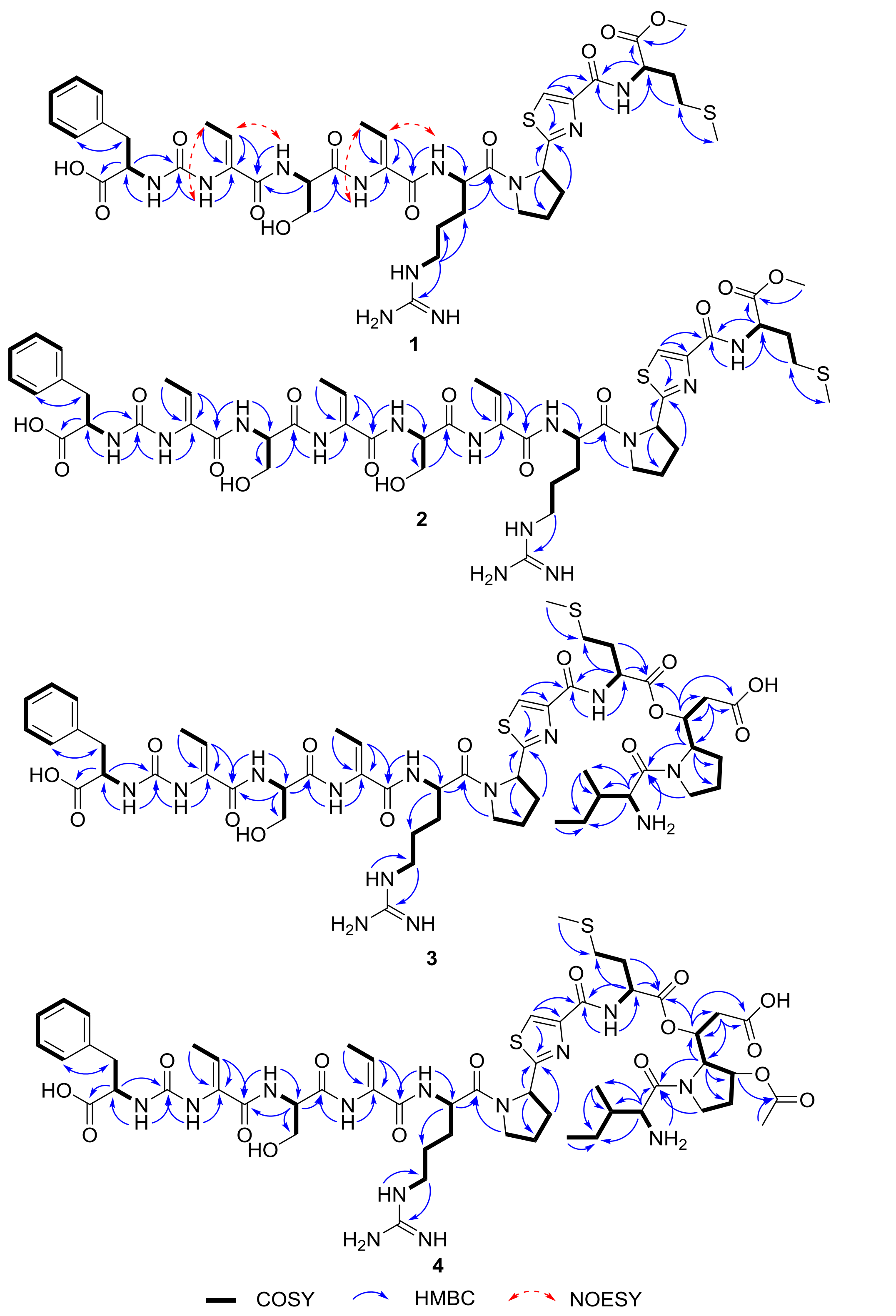


**Supplementary Fig. 6. Key COSY, HMBC and NOESY correlations of compounds 1-4.**

*Bacillus cereus*

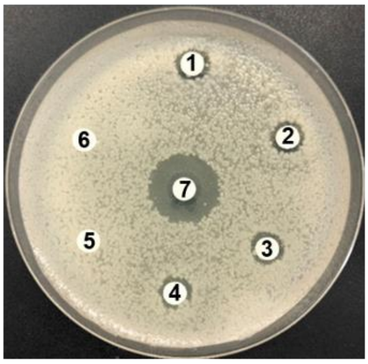

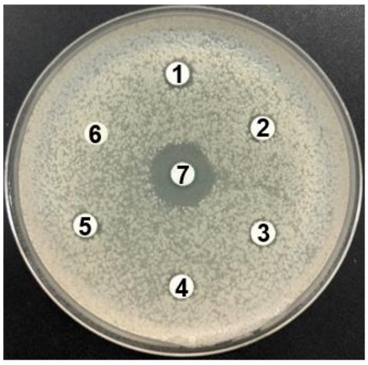

| | BS (μg/mL) | chm A (μg/mL) | chm B (μg/mL) | Inhibition Zone (mm) |
|---|---|---|---|---|
| 1 | 1000 | 10000 | 0 | 8.7 |
| 2 | 1000 | 5000 | 0 | 8.5 |
| 3 | 1000 | 0 | 10000 | 8.2 |
| 4 | 1000 | 0 | 5000 | 8.5 |
| 5 | 0 | 10000 | 0 | 0 |
| 6 | 0 | 0 | 10000 | 0 |
| 7 | 1000 | 0 | 0 | 19.2 |

| | BS (μg/mL) | chm C, D (μg/mL) | Inhibition Zone (mm) |
|---|---|---|---|
| 1 | 1000 | 10000 | 8.2 |
| 2 | 1000 | 8000 | 7.9 |
| 3 | 1000 | 5000 | 7.8 |
| 4 | 1000 | 2000 | 7.2 |
| 5 | 1000 | 1000 | 7.2 |
| 6 | 0 | 10000 | 0 |
| 7 | 1000 | 0 | 17.2 |

**Supplementary Fig. 7. The anti-antibiotic activity of chinitimides.** Disk diffusion assay of chitinimide A (**1**), chitinimide B (**2**), and the mixture of chitinimides C/D (**3**/**4**) as antagonist of the antibiotic blasticidin S (BS) against *B. cereus.* The assay plate is shown on the top. The mixture ratio of BS and chitinimides applied to each of the discs labelled 1-7 and tabulated results are shown in the corresponding table on the bottom. The plate shown on the left represents the anti-antibiotic activity of chmA and chmB as antagonist of the BS against *B.cereus*, respectively. Meanwhile the plate on the right represents the anti-antibiotic activity of chmC/D mixture as antagonist of the BS against *B.cereus*. Decreasing concentrations gradient of the chitinimides has no significant impact on the antagonism of BS (i.e., inhibition zone resembles).

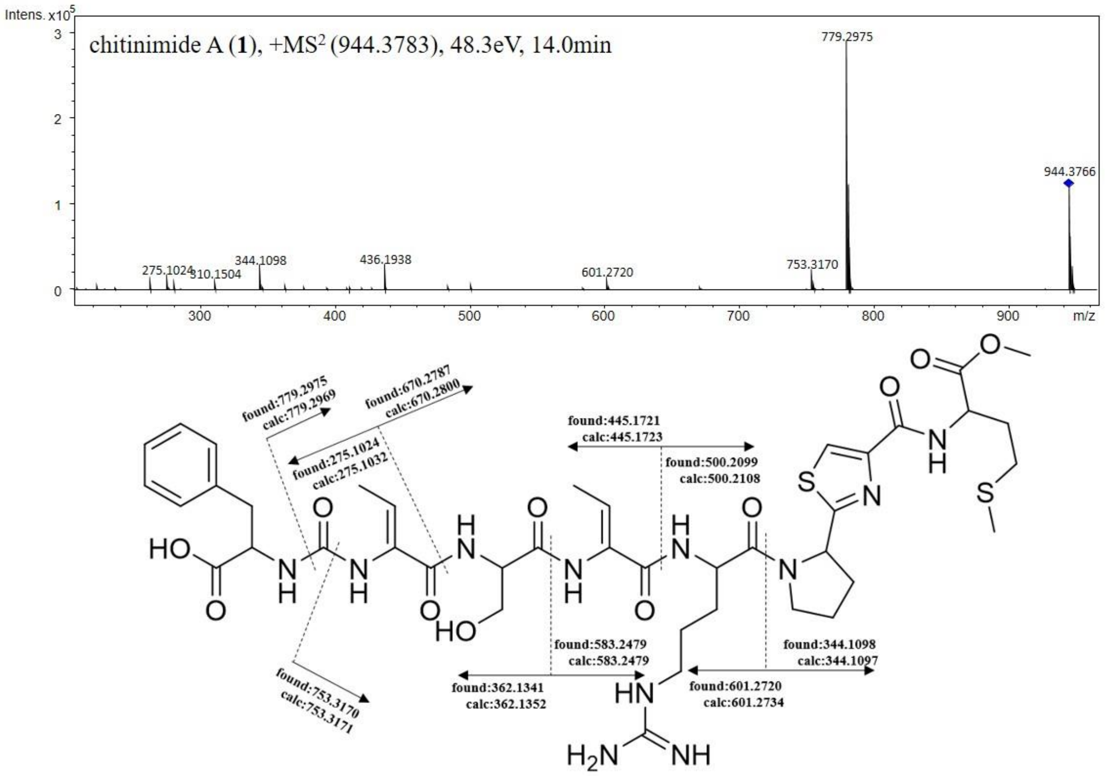


**Supplementary Fig. 8. HRESIMS spectra and MS/MS fragmentation of chitinimide A (1).**

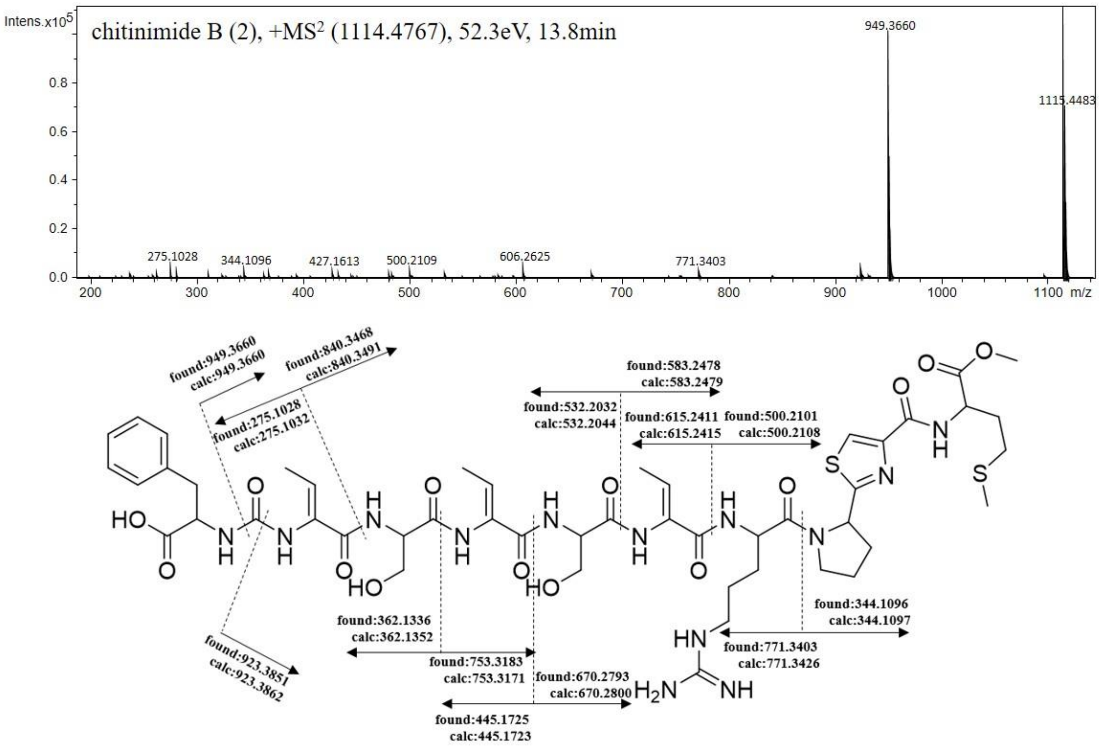


**Supplementary Fig. 9. HRESIMS spectra and MS/MS fragmentation of chitinimide B (2).**

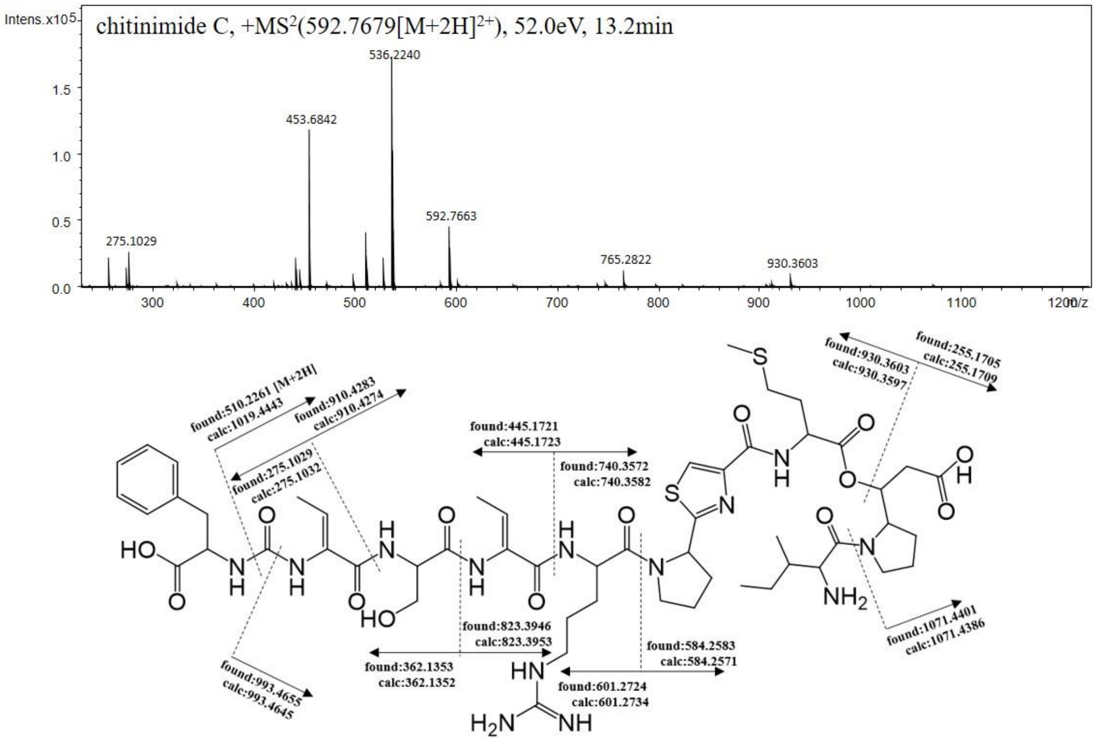


**Supplementary Fig. 10. HRESIMS spectra and MS/MS fragmentation of chitinimide C (3).**

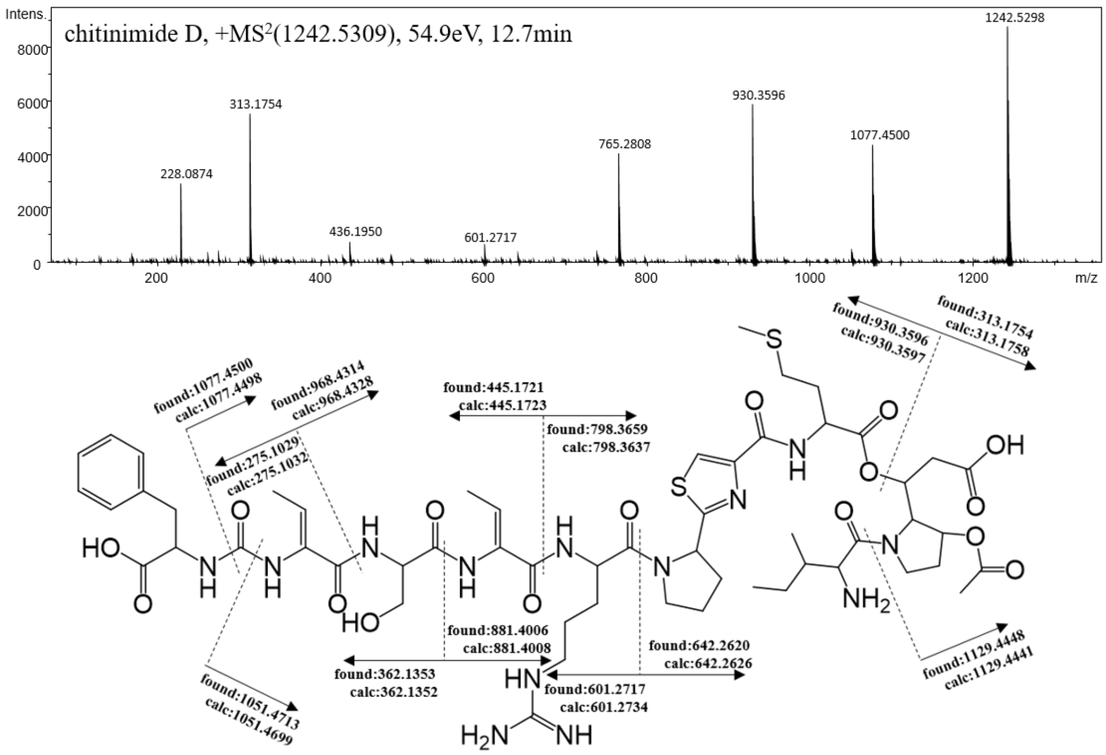


**Supplementary Fig. 11.** HRESIMS spectra and MS/MS fragmentation of chitinimide D (4).

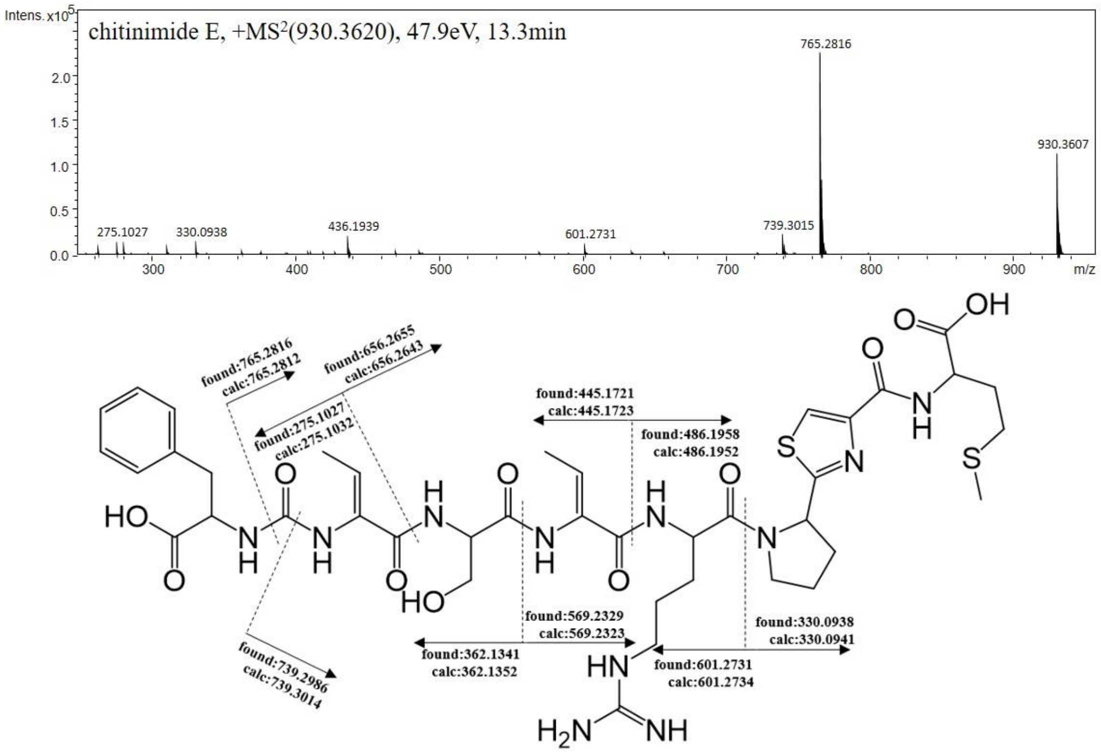


**Supplementary Fig. 12. HRESIMS spectra and MS/MS fragmentation of chitinimide E (5).**

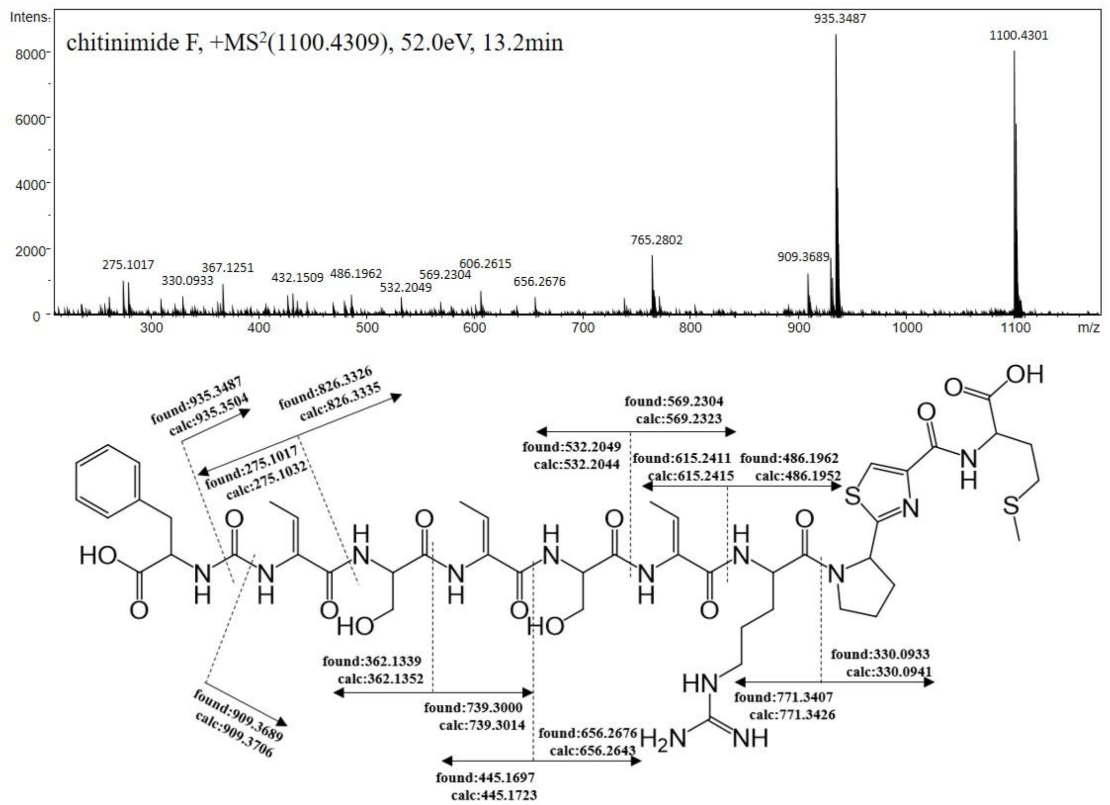


**Supplementary Fig. 13. HRESIMS spectra and MS/MS fragmentation of chitinimide F (6).**

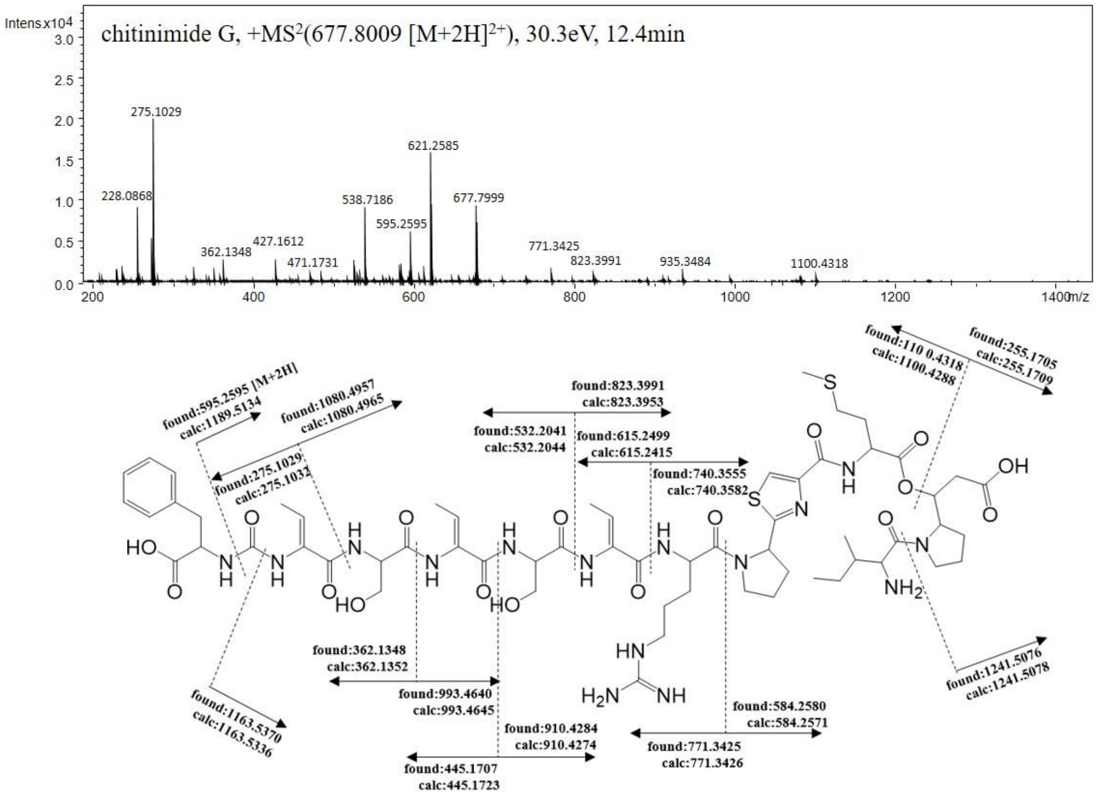


**Supplementary Fig. 14. HRESIMS spectra and MS/MS fragmentation of chitinimide G (7).**

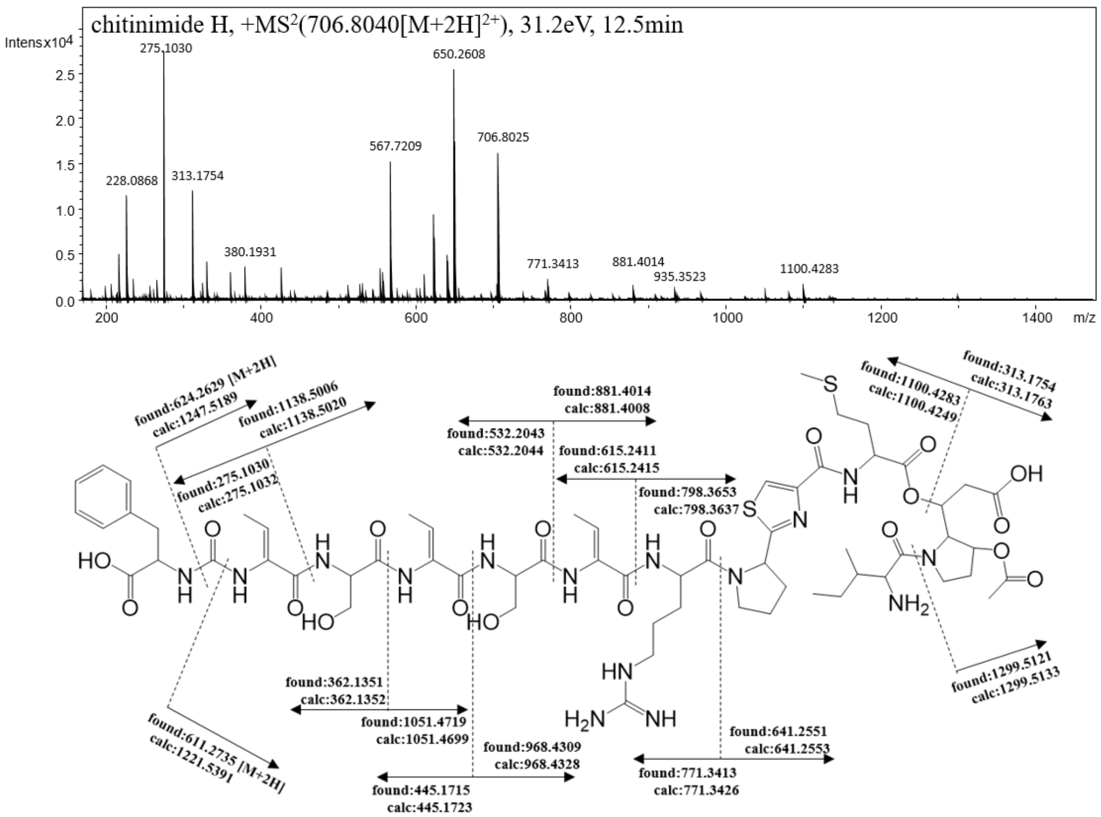


**Supplementary Fig. 15. HRESIMS spectra and MS/MS fragmentation of chitinimide H (8).**

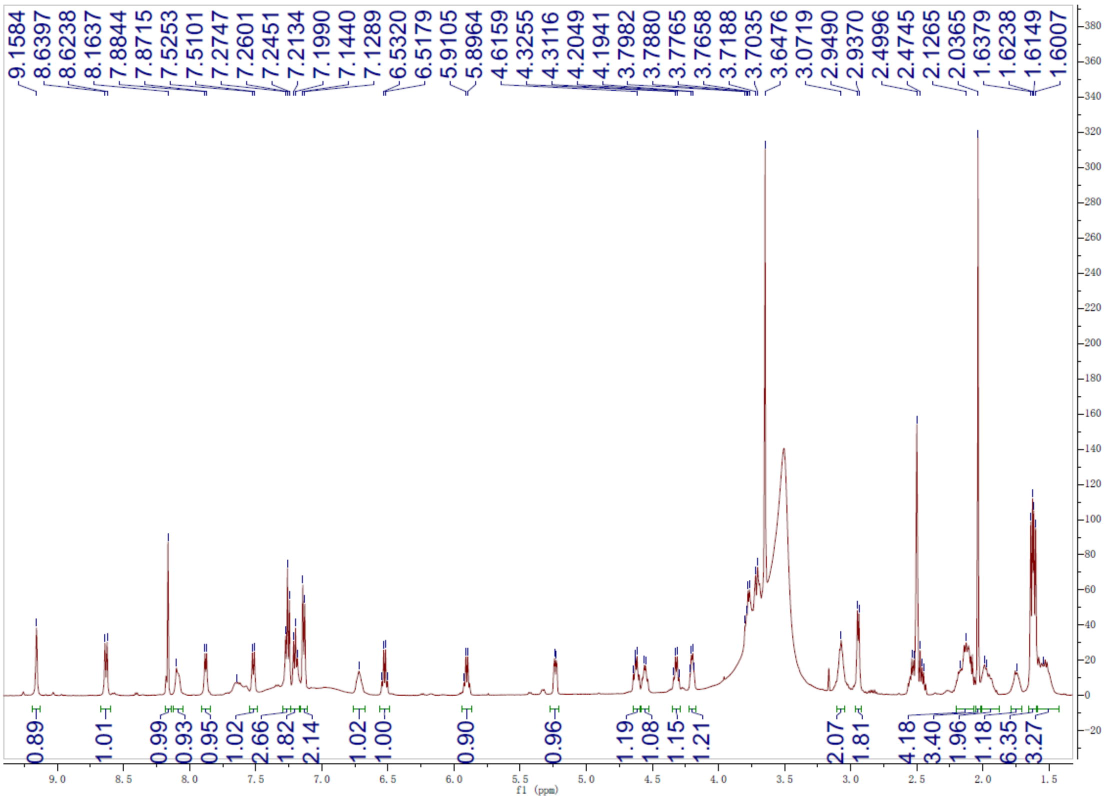

**Supplementary Fig. 16. $^{1}$H NMR spectrum of chitinimide A (1) in DMSO- $d_6$.**

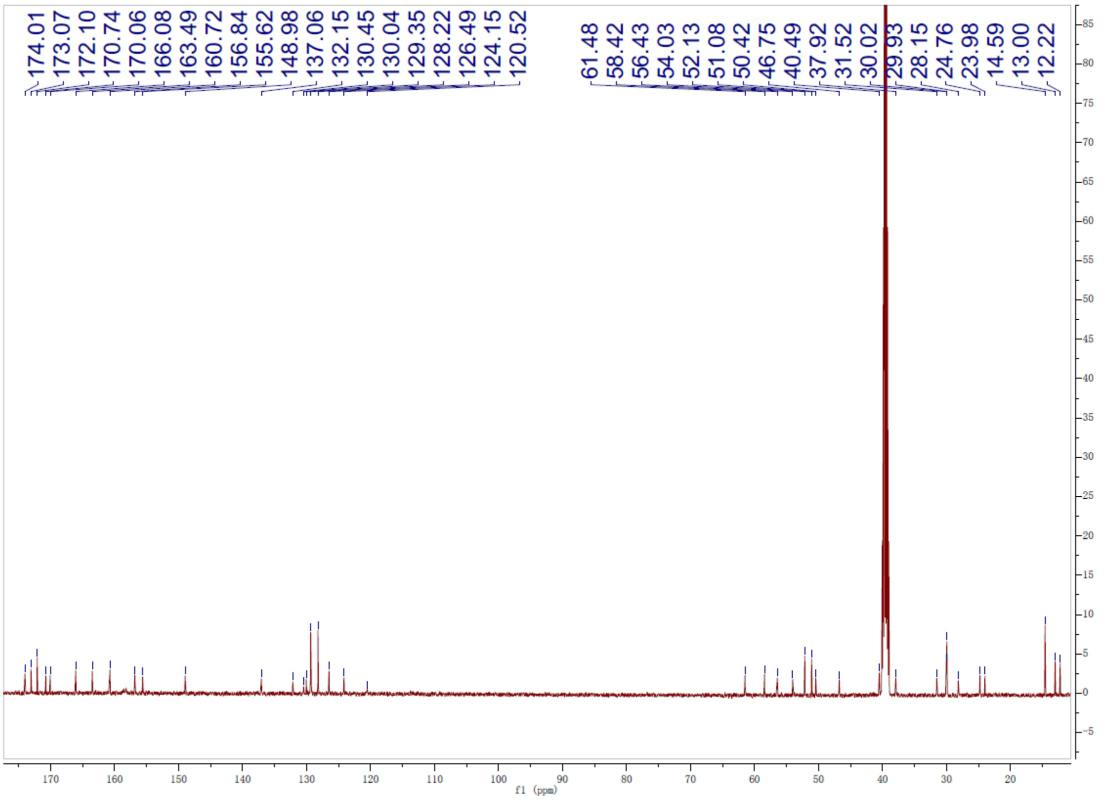

**Supplementary Fig. 17. $^{13}$C NMR spectrum of** chitinimide A (1) **in DMSO-$d_6$.**

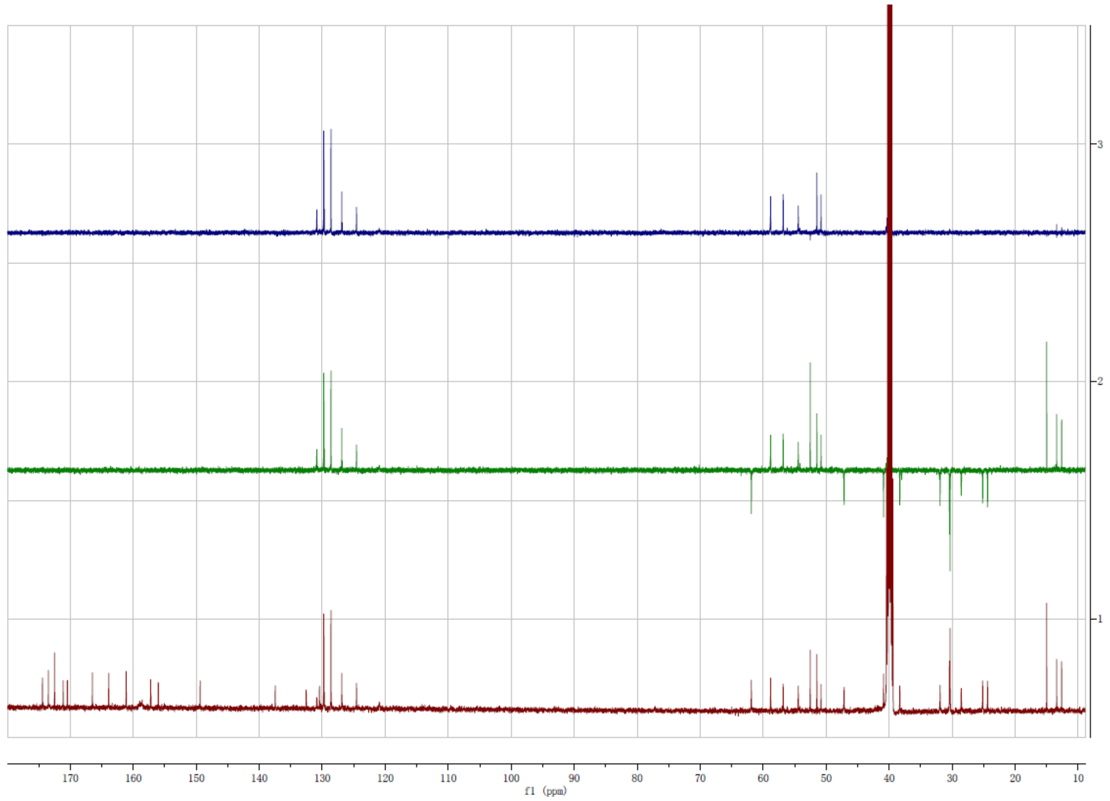


**Supplementary Fig. 18. DEPT spectrum of chitinimide A (1) in DMSO-*d*6.**

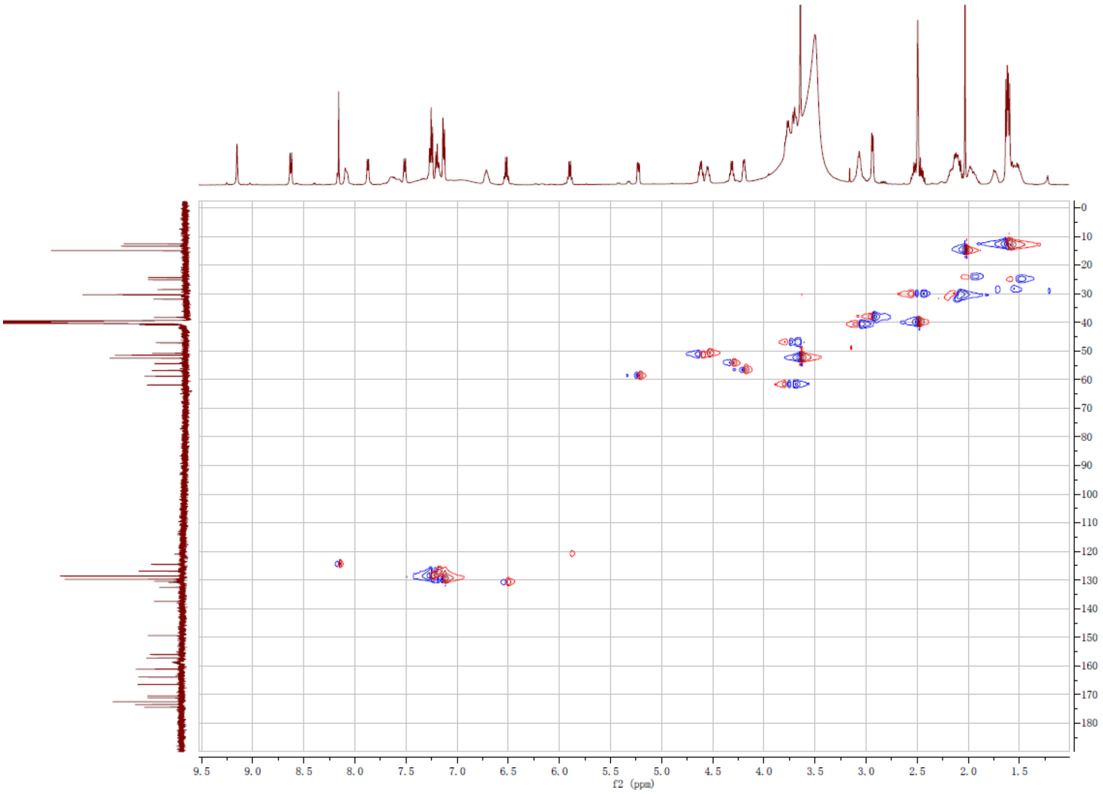


**Supplementary Fig. 19. HSQC spectrum of chitinimide A (1) in DMSO-*d*6.**

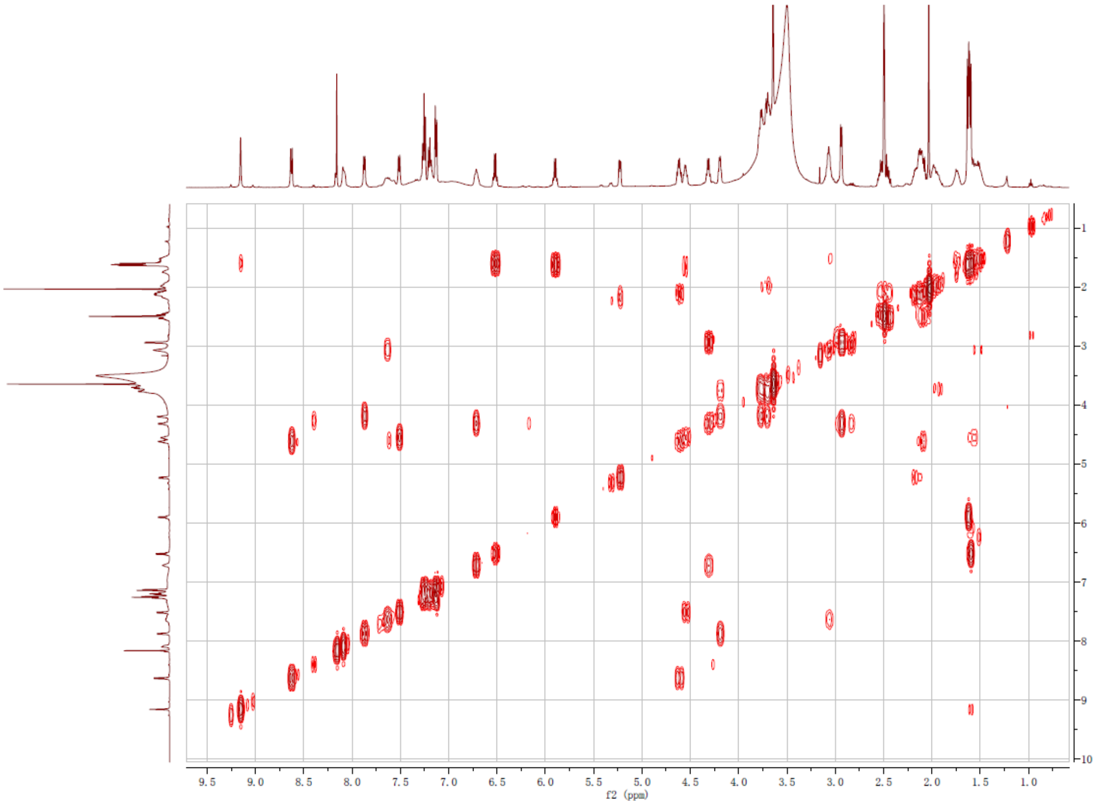


**Supplementary Fig. 20. $^{1}H$-$^{1}H$ COSY spectrum of chitinimide A (1) in DMSO-*d*$_6$.**

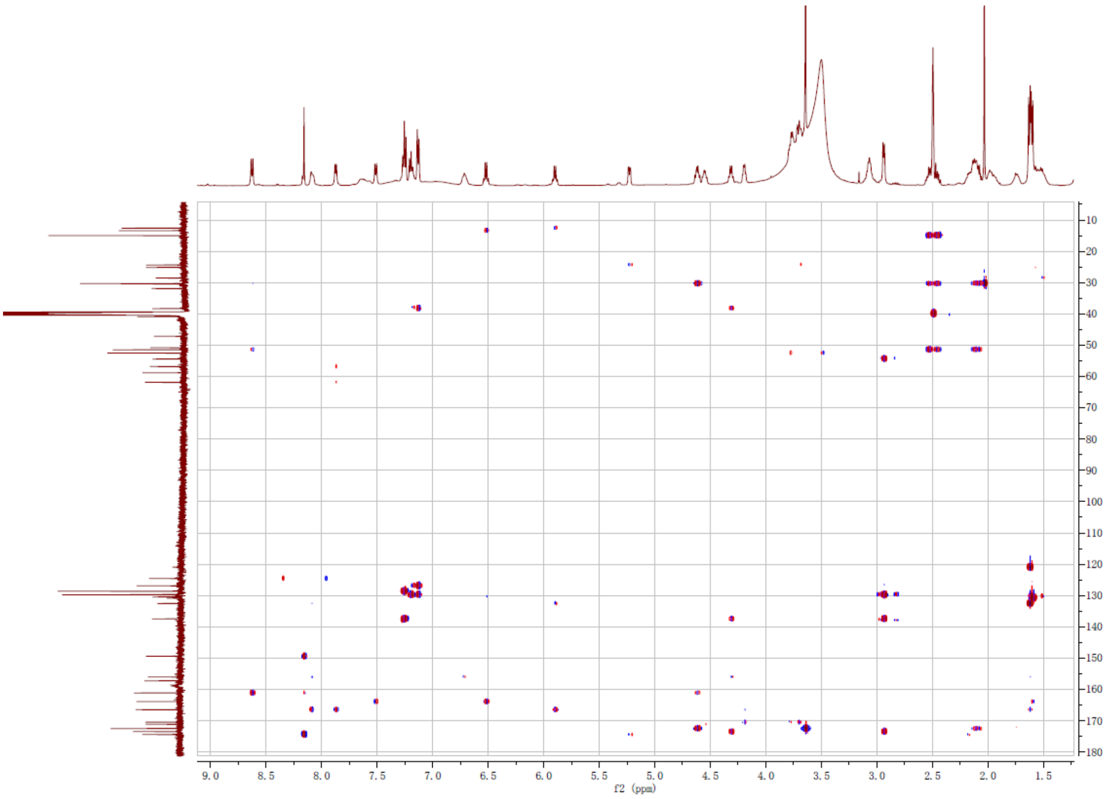


**Supplementary Fig. 21. HMBC spectrum of chitinimide A (1) in DMSO-*d*$_6$.**

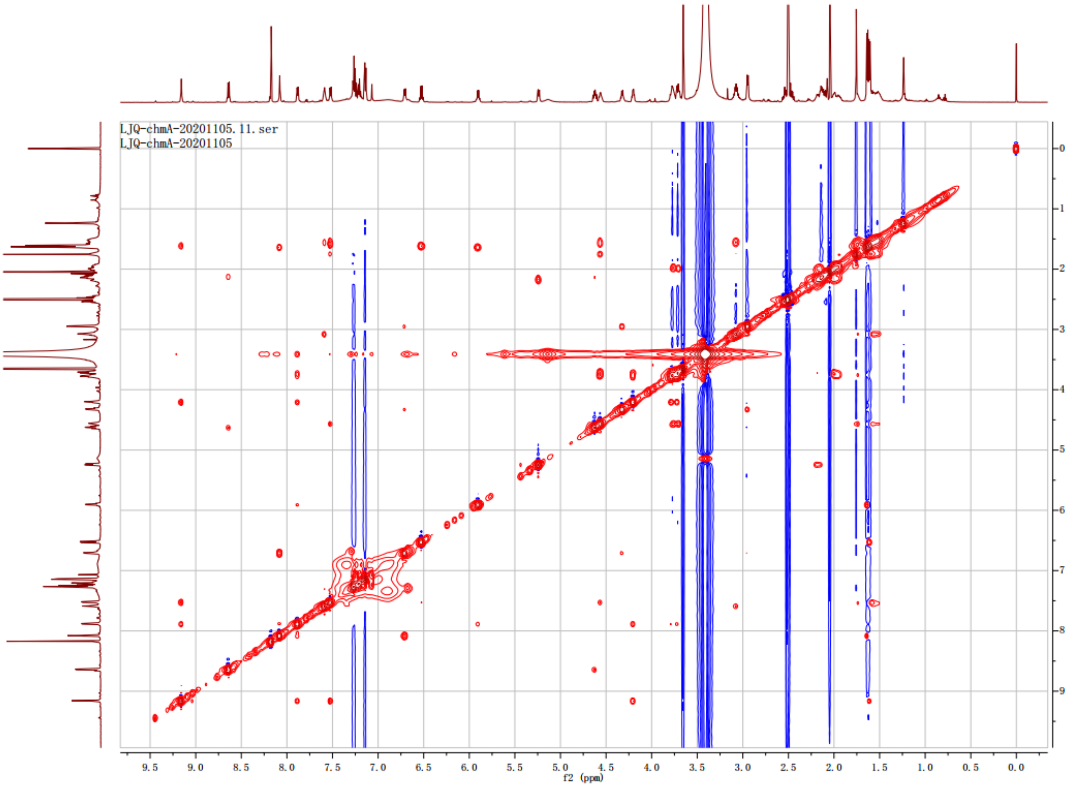


**Supplementary Fig. 22. NOESY spectrum of chitinimide A (1) in DMSO-*d*6.**

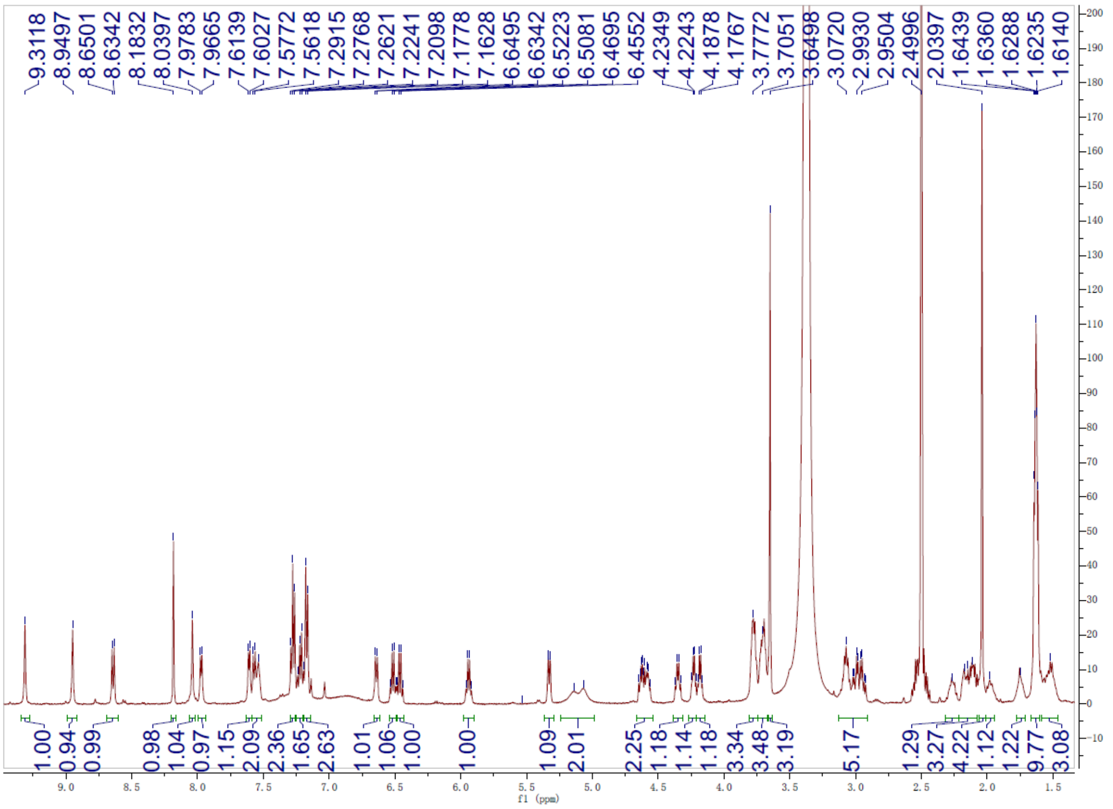


**Supplementary Fig. 23. $^1$H NMR spectrum of chitinimide B (2) in DMSO- *d*6.**

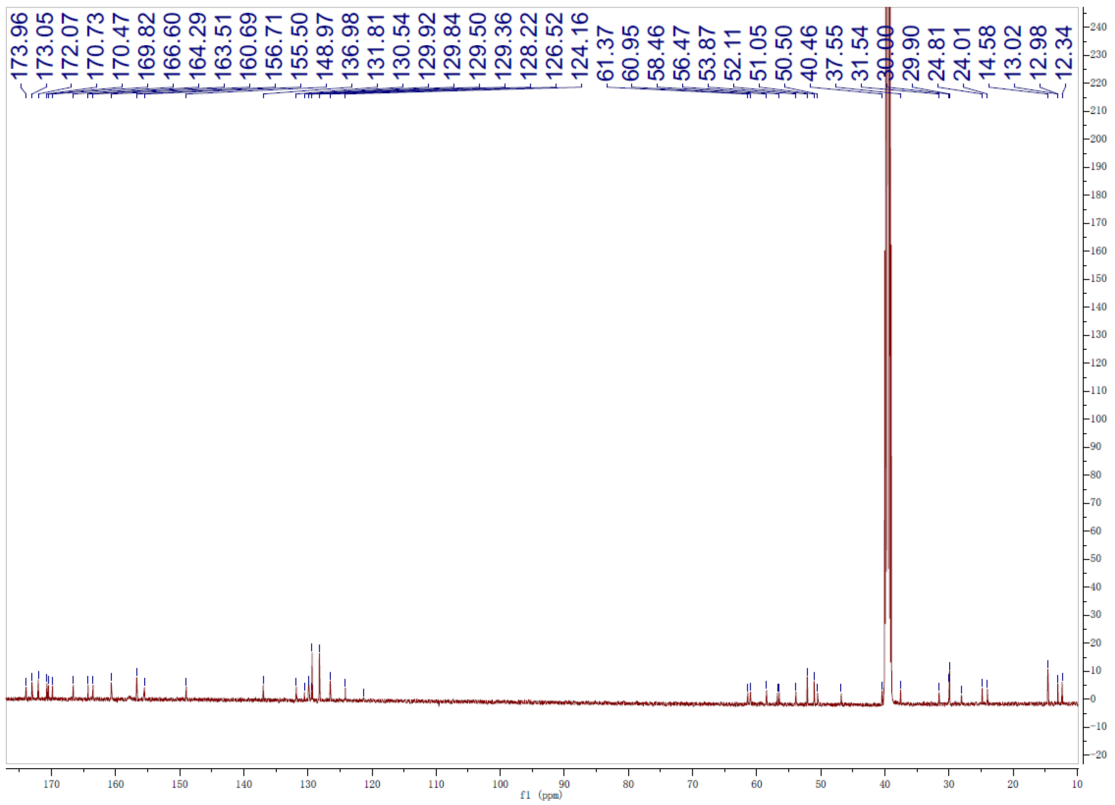


**Supplementary Fig. 24. $^{13}$C NMR spectrum of chitinimide B (2) in DMSO-*d*$_6$.**

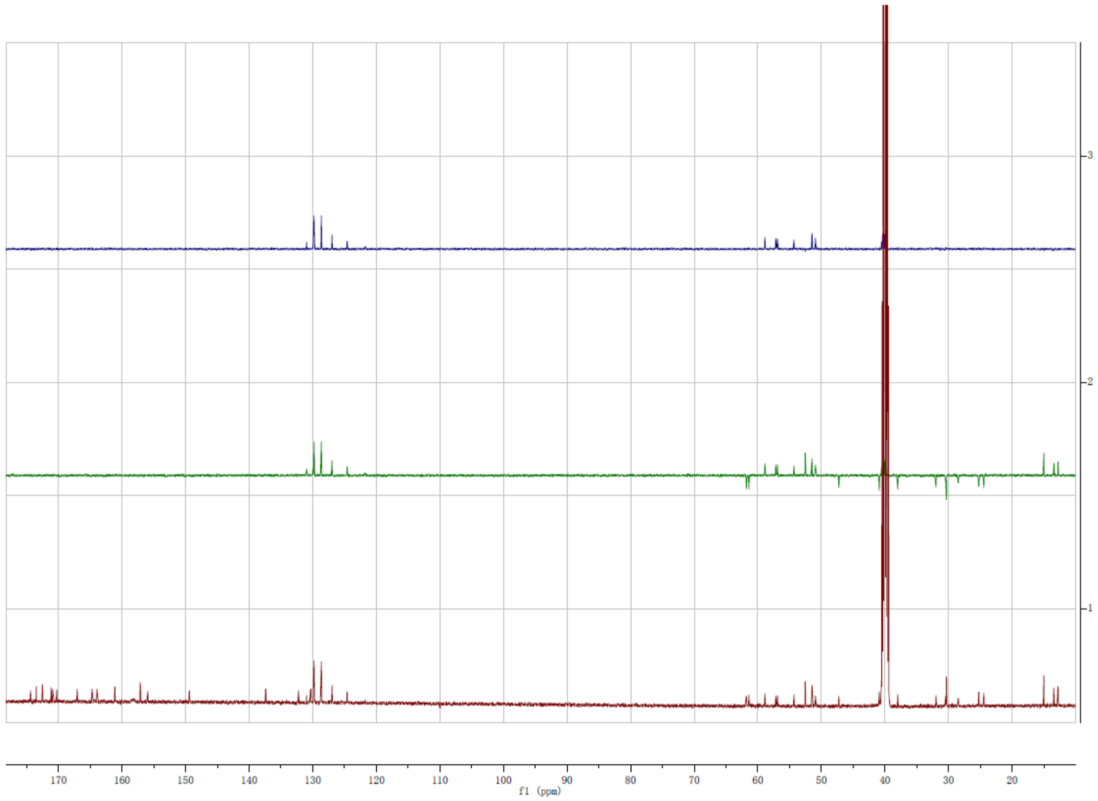


**Supplementary Fig. 25. DEPT spectrum of chitinimide B (2) in DMSO-*d*$_6$.**

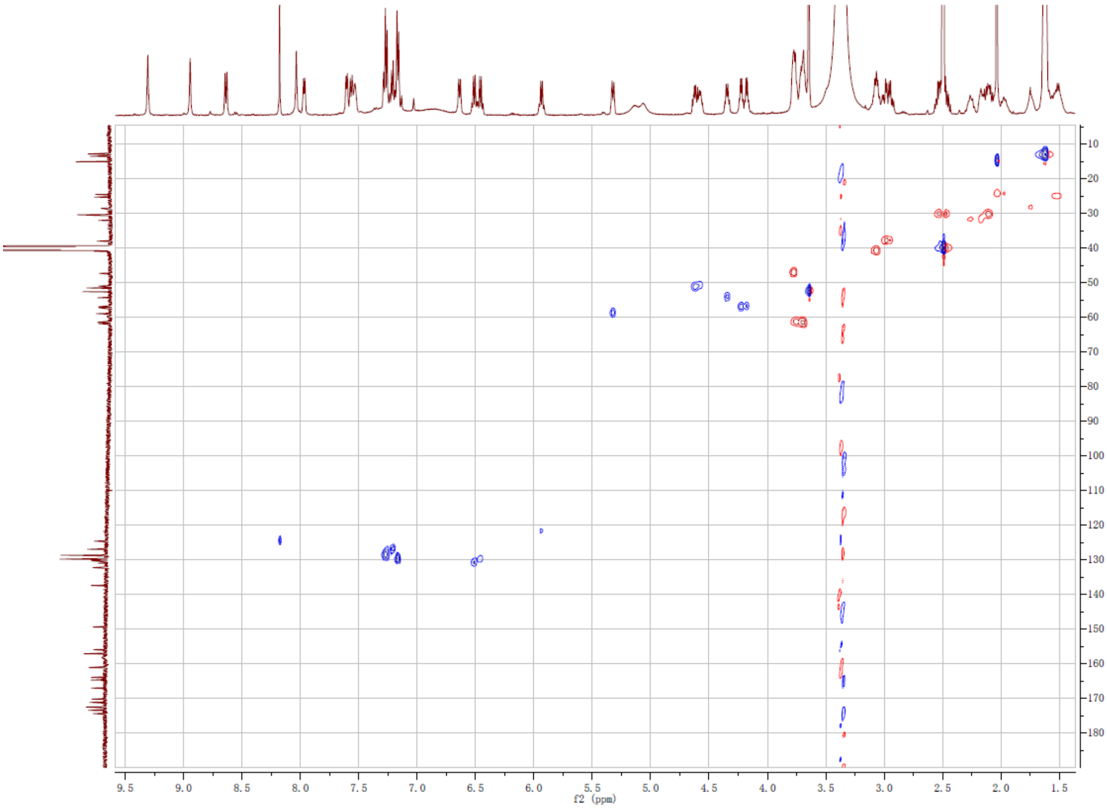


**Supplementary Fig. 26. HSQC spectrum of chitinimide B (2) in DMSO-*d*6.**

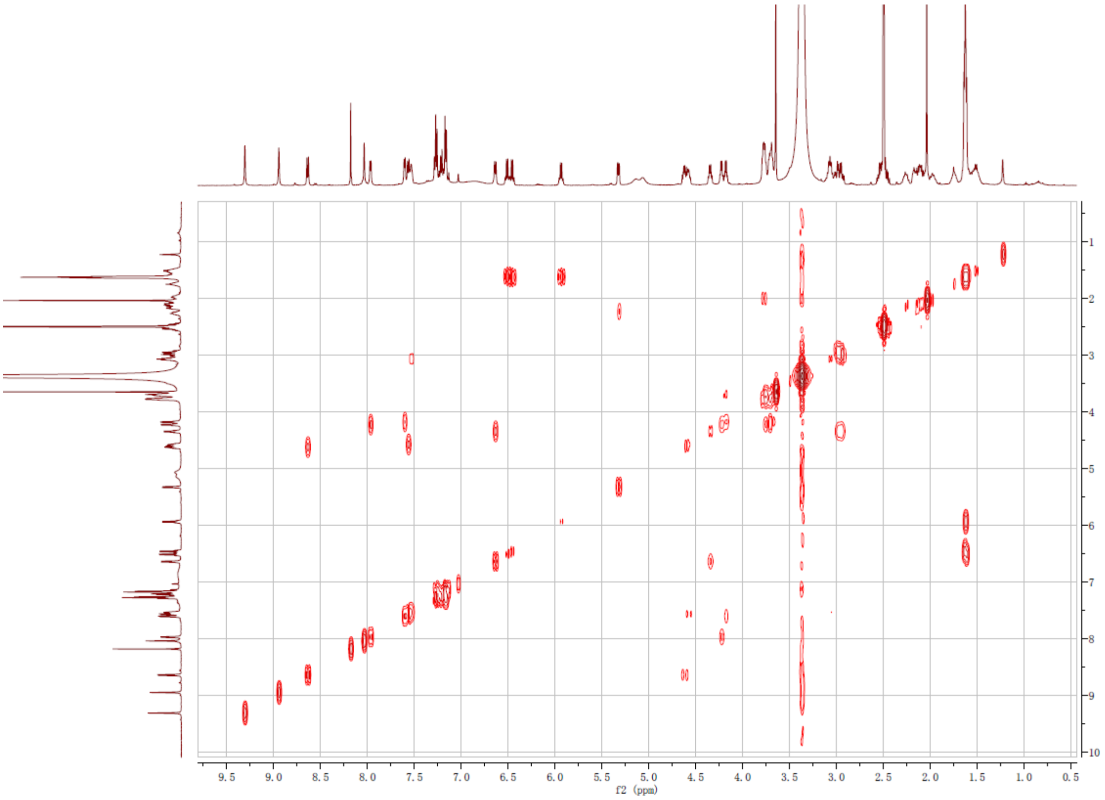


**Supplementary Fig. 27. $^{1}$H-$^{1}$H COSY spectrum of chitinimide B (2) in DMSO-*d*6.**

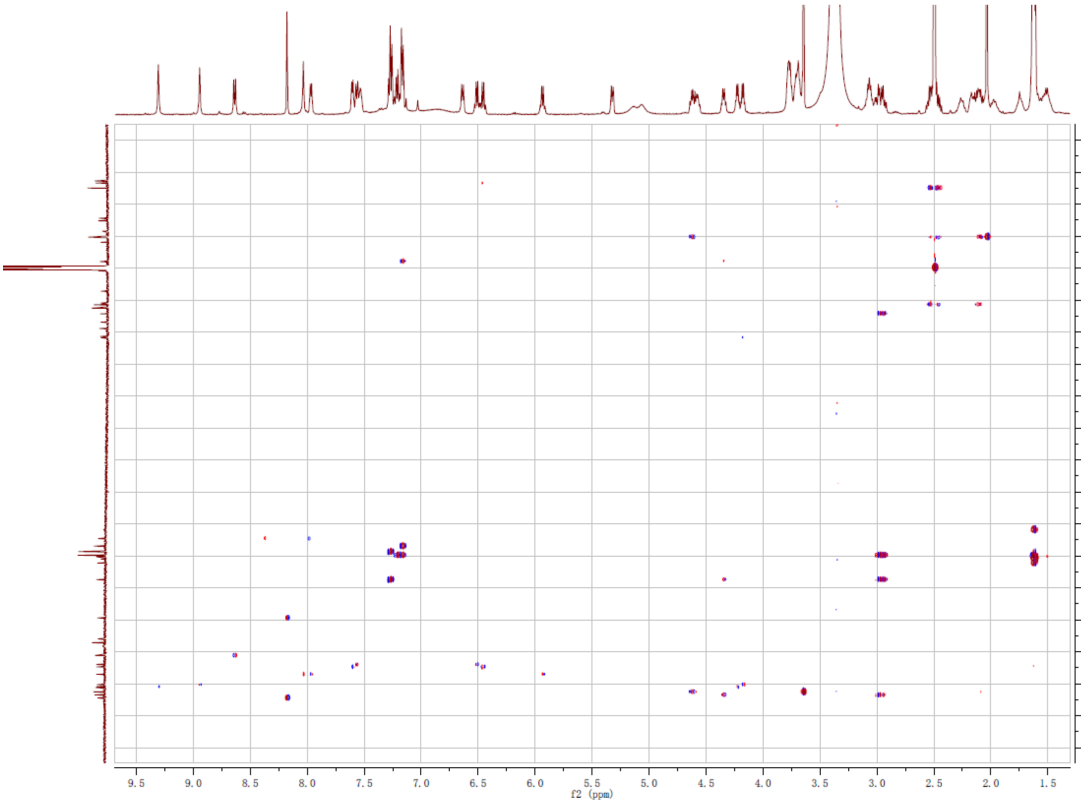


**Supplementary Fig. 28. HMBC spectrum of chitinimide B (2) in DMSO-*d*6.**

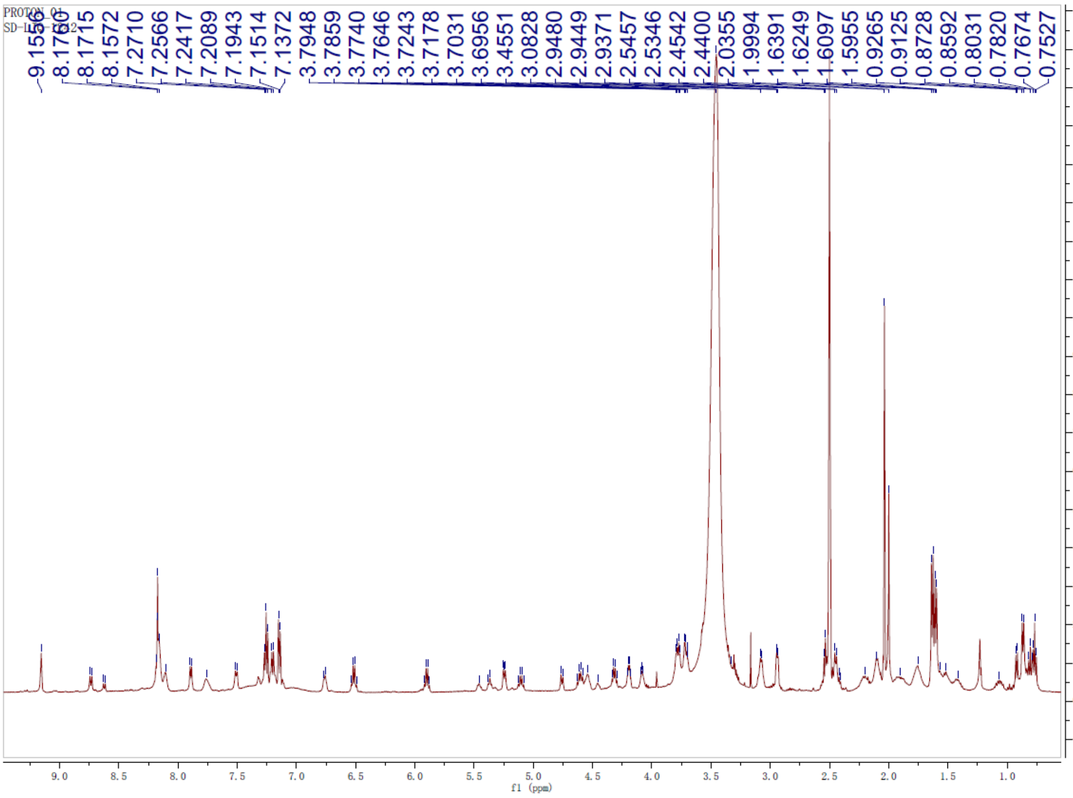


**Supplementary Fig. 29. $^{1}$H NMR spectrum of chitinimide C-D (3-4) in DMSO- *d*6.**

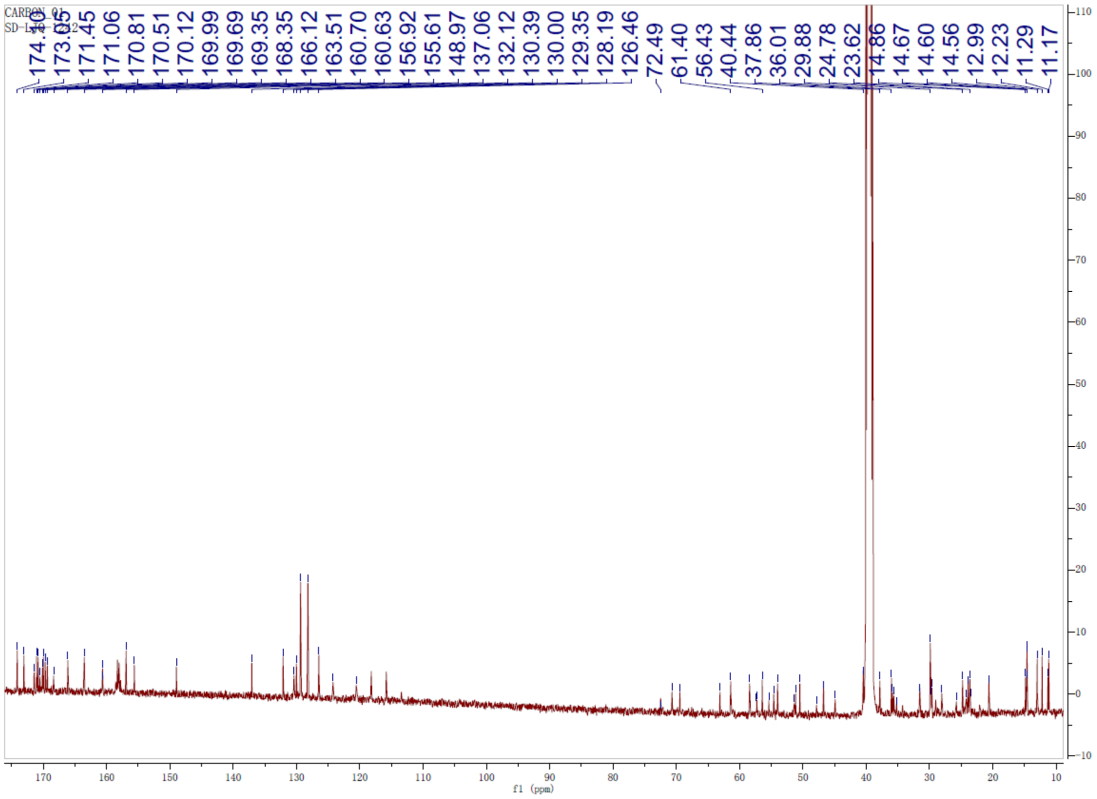

**Supplementary Fig. 30. $^{13}C$ NMR spectrum of chitinimide C-D (3-4) in DMSO-$d_6$.**

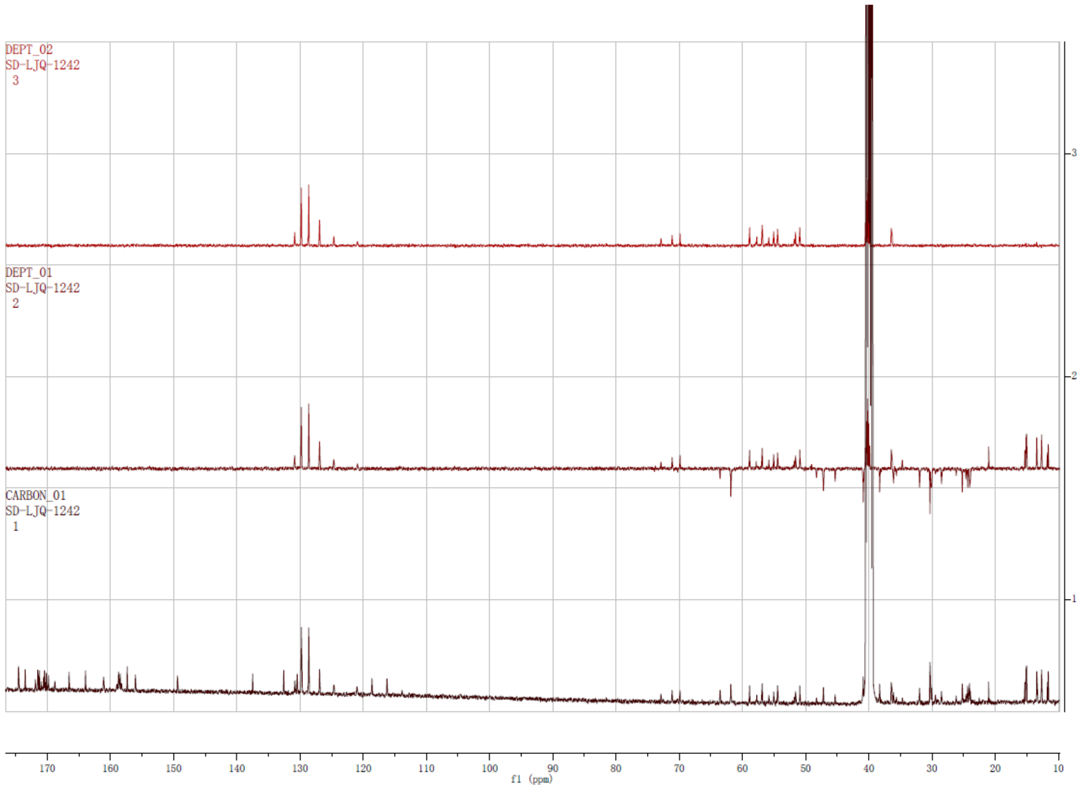

**Supplementary Fig. 31. DEPT spectrum of chitinimide C-D (3-4) in DMSO-$d_6$.**

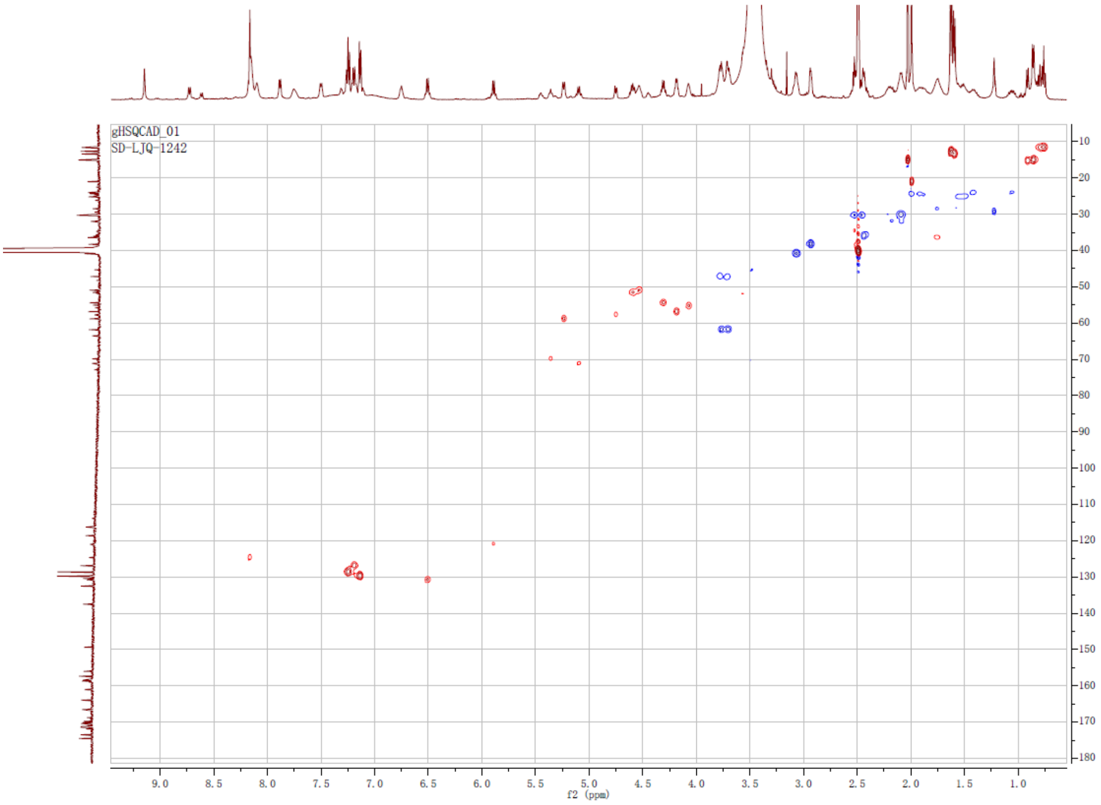


Supplementary Fig. 32. HSQC spectrum of chitinimide C-D (3-4) in DMSO-$d_6$.

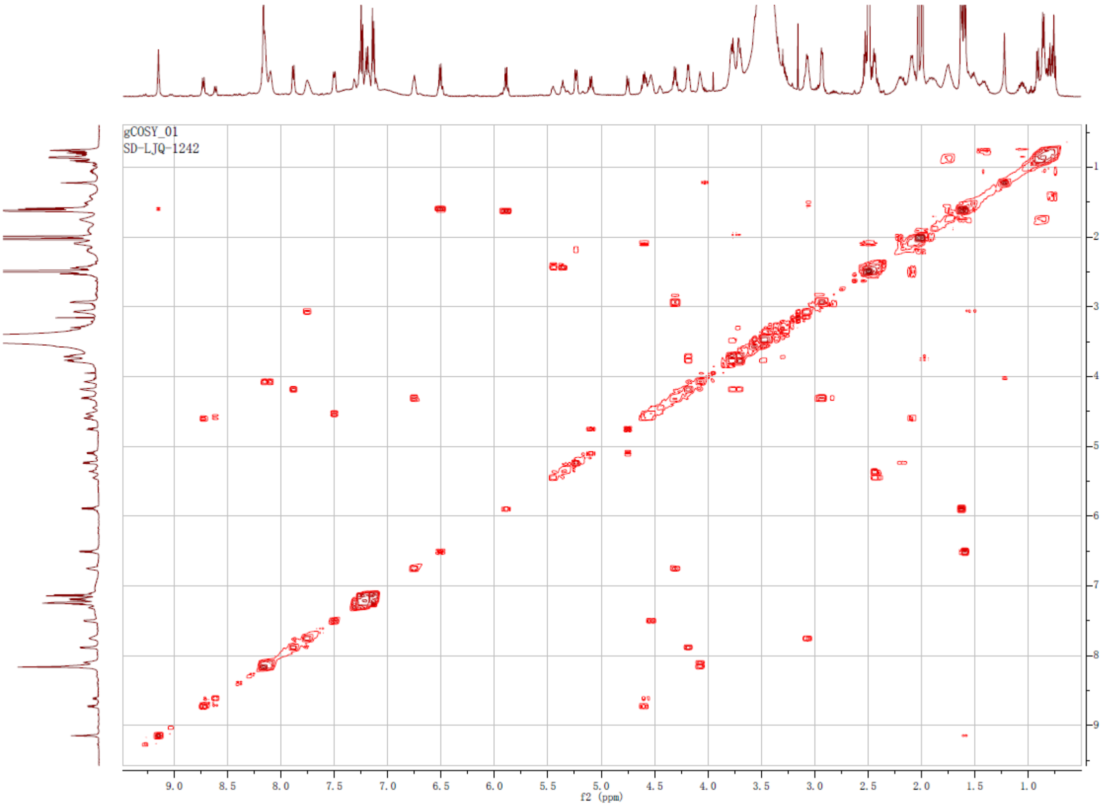


**Supplementary Fig. 33. $^{1}$H-$^{1}$H COSY spectrum of chitinimide C-D (3-4) in DMSO-*d*$_6$.**

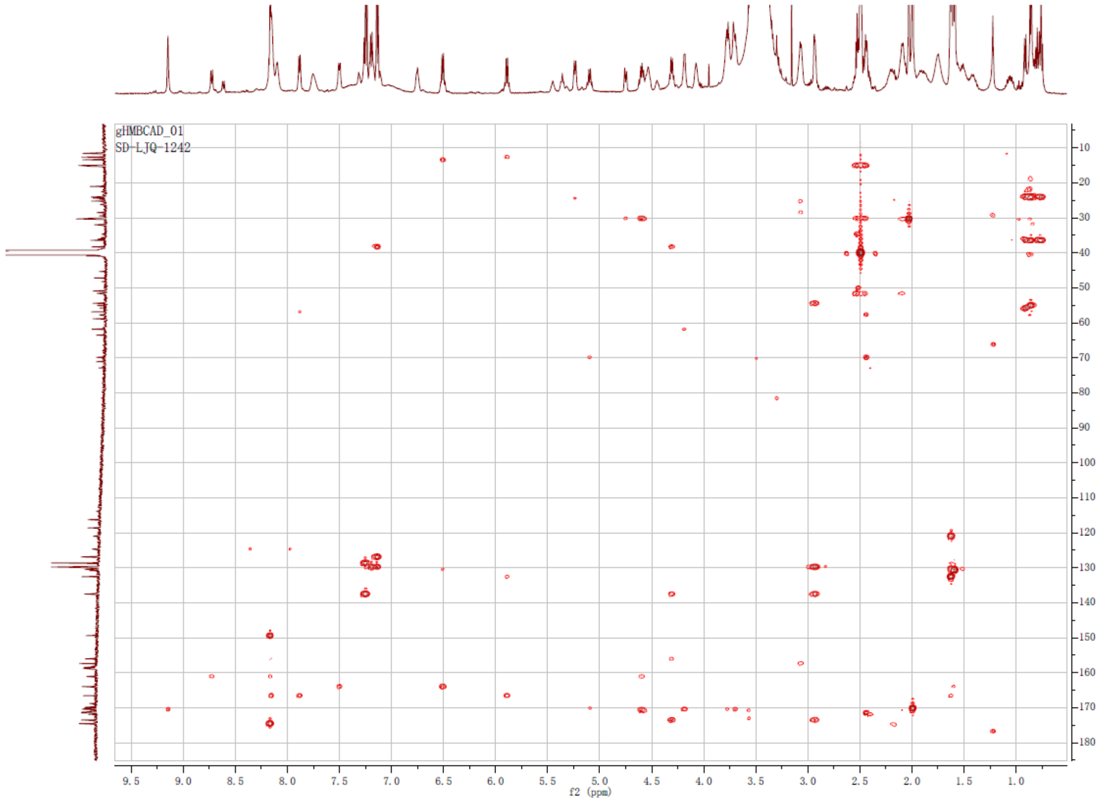


**Supplementary Fig. 34. HMBC spectrum of chitinimide C-D (3-4) in DMSO-*d*$_6$.**

## Supplementary references


1. Matsumoto, T.; Tanaka, T.; Kondo, A., Engineering metabolic pathways in *Escherichia coli* for constructing a "microbial chassis" for biochemical production. *Bioresour Technol* **2017,** *245*, 1362-1368.
2. Liu, Y.; Liu, L.; Li, J.; Du, G.; Chen, J., Synthetic biology toolbox and chassis development in *Bacillus subtilis*. *Trends Biotechnol* **2019,** *37*, 548-562.
3. Liu, Y.; Li, J.; Du, G.; Chen, J.; Liu, L., Metabolic engineering of *Bacillus subtilis* fueled by systems biology: Recent advances and future directions. *Biotechnol Adv* **2017,** *35*, 20-30.
4. Palazzotto, E.; Tong, Y. J.; Lee, S. Y.; Weber, T., Synthetic biology and metabolic engineering of actinomycetes for natural product discovery. *Biotechnol Adv* **2019,** *37*.
5. Loeschcke, A.; Thies, S., *Pseudomonas putida*-a versatile host for the production of natural products. *Appl Microbiol Biotechnol* **2015,** *99*, 6197-6214.
6. Ouyang, Q.; Wang, X.; Zhang, N.; Zhong, L.; Liu, J. Q.; Ding, X. M.; Zhang, Y. M.; Bian, X. Y., Promoter screening facilitates heterologous production of complex secondary metabolites in Burkholderiales strains. *ACS Synth Biol* **2020,** *9*, 457-460.
7. Wang, X.; Zhou, H. B.; Chen, H. N.; Jing, X. S.; Zheng, W. T.; Li, R. J.; Sun, T.; Liu, J. Q.; Fu, J.; Huo, L. J.; Li, Y. Z.; Shen, Y. M.; Ding, X. M.; Muller, R.; Bian, X. Y.; Zhang, Y. M., Discovery of recombinases enables genome mining of cryptic biosynthetic gene clusters in Burkholderiales species. *Proc. Natl. Acad. Sci. U. S. A.* **2018,** *115*, E4255-E4263.
8. Pogorevc, D.; Panter, F.; Schillinger, C.; Jansen, R.; Wenzel, S. C.; Muller, R., Production optimization and biosynthesis revision of corallopyronin A, a potent anti-filarial antibiotic. *Metab Eng* **2019,** *55*, 201-211.
9. Pogorevc, D.; Tang, Y.; Hoffmann, M.; Zipf, G.; Bernauer, H. S.; Popoff, A.; Steinmetz, H.; Wenzel, S. C., Biosynthesis and heterologous production of argyrins. *ACS Synth Biol* **2019,** *8*, 1121-1133.
