# Supplementary material for: Rational construction of genome-reduced Burkholderiales chassis facilitates efficient heterologous production of natural products from proteobacteria
Source: Nat Commun. 2021 Jul 23;12:4347. doi: 10.1038/s41467-021-24645-0 (PMC8302735; doi:10.1038/s41467-021-24645-0)
Supplement: Supplementary file 5 — Description of additional supplementary files [file 41467_2021_24645_MOESM5_ESM.docx]

Description of Additional Supplementary Data

**Supplementary Data 1**

Description: Genomic regions predicted to be deleted in DSM 7029 via bioinformatic analysis.

**Supplementary Data 2**

Description: Gene annotation of deleted genomic regions (transposases coding genes and other non-essential genes) in DSM 7029.

**Supplementary Data 3**

Description: Bacteria strains, plasmids and sequences of primers used in this study.
