# Supplementary material for: Rational construction of genome-reduced Burkholderiales chassis facilitates efficient heterologous production of natural products from proteobacteria
Source: Nat Commun. 2021 Jul 23;12:4347. doi: 10.1038/s41467-021-24645-0 (PMC8302735; doi:10.1038/s41467-021-24645-0)
Supplement: Supplementary file 6 — Reporting Summary [file 41467_2021_24645_MOESM6_ESM.pdf]

nature research

Corresponding author(s): Xiaoying Bian

Last updated by author(s): Jun 27, 2021

# Reporting Summary

Nature Research wishes to improve the reproducibility of the work that we publish. This form provides structure for consistency and transparency in reporting. For further information on Nature Research policies, see our Editorial Policies and the Editorial Policy Checklist.

## Statistics

For all statistical analyses, confirm that the following items are present in the figure legend, table legend, main text, or Methods section.

| n/a | Confirmed | |
|---|---|---|
| ☐ | ☒ | The exact sample size (*n*) for each experimental group/condition, given as a discrete number and unit of measurement |
| ☒ | ☐ | A statement on whether measurements were taken from distinct samples or whether the same sample was measured repeatedly |
| ☐ | ☒ | The statistical test(s) used AND whether they are one- or two-sided<br>*Only common tests should be described solely by name; describe more complex techniques in the Methods section.* |
| ☒ | ☐ | A description of all covariates tested |
| ☒ | ☐ | A description of any assumptions or corrections, such as tests of normality and adjustment for multiple comparisons |
| ☐ | ☒ | A full description of the statistical parameters including central tendency (e.g. means) or other basic estimates (e.g. regression coefficient) AND variation (e.g. standard deviation) or associated estimates of uncertainty (e.g. confidence intervals) |
| ☐ | ☒ | For null hypothesis testing, the test statistic (e.g. *F*, *t*, *r*) with confidence intervals, effect sizes, degrees of freedom and *P* value noted<br>*Give P values as exact values whenever suitable.* |
| ☒ | ☐ | For Bayesian analysis, information on the choice of priors and Markov chain Monte Carlo settings |
| ☒ | ☐ | For hierarchical and complex designs, identification of the appropriate level for tests and full reporting of outcomes |
| ☒ | ☐ | Estimates of effect sizes (e.g. Cohen's *d*, Pearson's *r*), indicating how they were calculated |

*Our web collection on statistics for biologists contains articles on many of the points above.*

## Software and code

Policy information about availability of computer code

| | |
|---|---|
| Data collection | UPLC/MS or UPLC/HRMS data were collected from Ultimate 3000 UPLC (Thermo Fisher) connected to Bruker amaZon SL ESI - Ion Trap Mass Spectrometer or Bruker Impact HD Mass Spectrometer; NMR data were collected from Bruker Avance III-600 spectrometer. |
| Data analysis | DataAnalysis (v4.2); Microsoft Word & Excel & PowerPoint (2019); Adobe Illustrator CS4; GraphPad Prism (v6.01) |

For manuscripts utilizing custom algorithms or software that are central to the research but not yet described in published literature, software must be made available to editors and reviewers. We strongly encourage code deposition in a community repository (e.g. GitHub). See the Nature Research guidelines for submitting code & software for further information.

## Data

Policy information about availability of data

All manuscripts must include a data availability statement. This statement should provide the following information, where applicable:

- Accession codes, unique identifiers, or web links for publicly available datasets
- A list of figures that have associated raw data
- A description of any restrictions on data availability

All data are available in the main text, Supplementary Information, including experimental details, mutants, and NMR spectra. A reporting summary for this article is available as a Supplementary Information file. The bacteria materials generated during the current study are available from the corresponding author upon reasonable request. The chitinimide BGC has been deposited in the Genbank under accession number "MW160162 [https://www.ncbi.nlm.nih.gov/nuccore/MW160162]". Source data are provided with this paper.

# Field-specific reporting

Please select the one below that is the best fit for your research. If you are not sure, read the appropriate sections before making your selection.

☒ Life sciences ☐ Behavioural & social sciences ☐ Ecological, evolutionary & environmental sciences

For a reference copy of the document with all sections, see nature.com/documents/nr-reporting-summary-flat.pdf

# Life sciences study design

All studies must disclose on these points even when the disclosure is negative.

| | |
|---|---|
| Sample size | No statistical method was used to predetermine the sample size. For n=3 independent biological replicates, individual replicate number were given in the figure legend. According to previous experiments, the sample size has been determined to be sufficient to ensure reproducibility and make statistics. |
| Data exclusions | No data were excluded. |
| Replication | The experimental findings in all figures were reproduced successfully. |
| Randomization | Samples were not randomized, and randomization was not need for the study as this work dose not involve particular animals or groups. |
| Blinding | The investigators were not blinded to group allocation, and the statistic data were collected from the raw data. |

# Behavioural & social sciences study design

All studies must disclose on these points even when the disclosure is negative.

| | |
|---|---|
| Study description | n/a |
| Research sample | n/a |
| Sampling strategy | n/a |
| Data collection | n/a |
| Timing | n/a |
| Data exclusions | n/a |
| Non-participation | n/a |
| Randomization | n/a |

# Ecological, evolutionary & environmental sciences study design

All studies must disclose on these points even when the disclosure is negative.

| | |
|---|---|
| Study description | n/a |
| Research sample | n/a |
| Sampling strategy | n/a |
| Data collection | n/a |
| Timing and spatial scale | n/a |
| Data exclusions | n/a |
| Reproducibility | n/a |
| Randomization | n/a |

Blinding: n/a

Did the study involve field work? ☐ Yes ☒ No

# Reporting for specific materials, systems and methods

We require information from authors about some types of materials, experimental systems and methods used in many studies. Here, indicate whether each material, system or method listed is relevant to your study. If you are not sure if a list item applies to your research, read the appropriate section before selecting a response.

## Materials & experimental systems

| n/a | Involved in the study |
|---|---|
| ☒ | ☐ Antibodies |
| ☐ | ☒ Eukaryotic cell lines |
| ☒ | ☐ Palaeontology and archaeology |
| ☒ | ☐ Animals and other organisms |
| ☒ | ☐ Human research participants |
| ☒ | ☐ Clinical data |
| ☒ | ☐ Dual use research of concern |

## Methods

| n/a | Involved in the study |
|---|---|
| ☒ | ☐ ChIP-seq |
| ☒ | ☐ Flow cytometry |
| ☒ | ☐ MRI-based neuroimaging |

## Antibodies

Antibodies used: n/a

Validation: n/a

## Eukaryotic cell lines

Policy information about cell lines

Cell line source(s): Human acute lymphoblastic leukemia cells Kasumi used in cytotoxicity assay was obtained from ATCC. Human breast cancer cells MDA-MB-231, human lung carcinoma cell line A549, and human normal lung epithelial cells line BEAS-2B used in cytotoxicity assay were obtained from Beyotime Biotechnology.

Authentication: The ATCC used SRT and isoenzyme profiles for cell line authentication. The Beyotime Biotechnology used SRT for cell line authentication. Additional authentication in the laboratory was performed regularly based on morphology and gene/protein expression.

Mycoplasma contamination: Cell lines were periodically tested for mycoplasma contamination and confirmed negative.

Commonly misidentified lines (See ICLAC register): No commonly misidentified cell lines were used in the study.

## Palaeontology and Archaeology

Specimen provenance: n/a

Specimen deposition: n/a

Dating methods: n/a

☐ Tick this box to confirm that the raw and calibrated dates are available in the paper or in Supplementary Information.

Ethics oversight: n/a

Note that full information on the approval of the study protocol must also be provided in the manuscript.

## Animals and other organisms

Policy information about studies involving animals; ARRIVE guidelines recommended for reporting animal research

Laboratory animals: n/a

Wild animals: n/a

Field-collected samples: n/a

| | |
|---|---|
| Ethics oversight | n/a |

Note that full information on the approval of the study protocol must also be provided in the manuscript.

# Human research participants

Policy information about studies involving human research participants

| | |
|---|---|
| Population characteristics | n/a |
| Recruitment | n/a |
| Ethics oversight | n/a |

Note that full information on the approval of the study protocol must also be provided in the manuscript.

# Clinical data

Policy information about clinical studies

All manuscripts should comply with the ICMJE guidelines for publication of clinical research and a completed CONSORT checklist must be included with all submissions.

| | |
|---|---|
| Clinical trial registration | n/a |
| Study protocol | n/a |
| Data collection | n/a |
| Outcomes | n/a |

# ChIP-seq

## Data deposition

- [ ] Confirm that both raw and final processed data have been deposited in a public database such as GEO.
- [ ] Confirm that you have deposited or provided access to graph files (e.g. BED files) for the called peaks.

| | |
|---|---|
| Data access links<br>*May remain private before publication.* | n/a |
| Files in database submission | n/a |
| Genome browser session<br>(e.g. UCSC) | n/a |

## Methodology

| | |
|---|---|
| Replicates | n/a |
| Sequencing depth | n/a |
| Antibodies | n/a |
| Peak calling parameters | n/a |
| Data quality | n/a |
| Software | n/a |

## Flow Cytometry

### Plots

Confirm that:

- [ ] The axis labels state the marker and fluorochrome used (e.g. CD4-FITC).
- [ ] The axis scales are clearly visible. Include numbers along axes only for bottom left plot of group (a 'group' is an analysis of identical markers).
- [ ] All plots are contour plots with outliers or pseudocolor plots.
- [ ] A numerical value for number of cells or percentage (with statistics) is provided.

### Methodology

| | |
|---|---|
| Sample preparation | n/a |
| Instrument | n/a |
| Software | n/a |
| Cell population abundance | n/a |
| Gating strategy | n/a |

- [ ] Tick this box to confirm that a figure exemplifying the gating strategy is provided in the Supplementary Information.

## Magnetic resonance imaging

### Experimental design

| | |
|---|---|
| Design type | n/a |
| Design specifications | n/a |
| Behavioral performance measures | n/a |

### Acquisition

| | |
|---|---|
| Imaging type(s) | n/a |
| Field strength | n/a |
| Sequence & imaging parameters | n/a |
| Area of acquisition | n/a |

Diffusion MRI ☐ Used ☐ Not used

### Preprocessing

| | |
|---|---|
| Preprocessing software | n/a |
| Normalization | n/a |
| Normalization template | n/a |
| Noise and artifact removal | n/a |
| Volume censoring | n/a |

### Statistical modeling & inference

| | |
|---|---|
| Model type and settings | n/a |
| Effect(s) tested | n/a |

Specify type of analysis: ☐ Whole brain ☐ ROI-based ☐ Both

Statistic type for inference
(See Eklund et al. 2016)

n/a

Correction

n/a

## Models & analysis

| n/a | Involved in the study |
|---|---|
| ☒ | ☐ Functional and/or effective connectivity |
| ☒ | ☐ Graph analysis |
| ☒ | ☐ Multivariate modeling or predictive analysis |
